# Parasite traits, host traits, and environment as determinants of dark diversity affinity in flea and gamasid mite assemblages from the Palearctic

**DOI:** 10.1007/s00436-024-08408-6

**Published:** 2024-11-26

**Authors:** Boris R. Krasnov, Maxim V. Vinarski, Natalia P. Korallo-Vinarskaya, Irina S. Khokhlova, Vasily I. Grabovsky

**Affiliations:** 1 Mitrani Department of Desert Ecology, Swiss Institute for Dryland Environmental and Energy Research, Jacob Blaustein Institutes for Desert Research, Ben-Gurion University of the Negev, Sede Boqer Campus, 84990 Midreshet Ben-Gurion, Israel; 2 Laboratory of Macroecology and Biogeography of Invertebrates, Saint-Petersburg State University, Saint-Petersburg, Russian Federation; 3 Laboratory of Parasitology, Zoological Institute of Russian Academy of Sciences, Saint-Petersburg, Russian Federation; 4 French Associates Institute for Agriculture and Biotechnology of Drylands, Jacob Blaustein Institutes for Desert Research, Ben-Gurion University of the Negev, Sede Boqer Campus, Midreshet Ben-Gurion, Israel

**Keywords:** Biogeography, Component community, Compound community, Ectoparasites, Metacommunity, Species pool

## Abstract

A species set in a site comprises species that are present (realized diversity) and species that could inhabit this site but are absent (dark diversity; DD). DD can be both species-driven (a species’ traits preclude its presence, independently of site features) and site-driven (site features preclude the species’ presence, independently of its traits). DD affinity (DDA) is a measure of species’ tendencies to be absent from sites that they could inhabit or of sites’ tendencies to lack species that could be present. Decomposition of DDA into DDA for species (*dda*_*sp*_) and for sites (*dda*_*site*_) allows (a) disentangling these two mechanisms and (b) detecting species traits and site features contributing to their DDA. The species-site unified model is a Bayesian statistical model aimed at simultaneously estimating *dda*_*sp*_ and *dda*_*site*_. We applied it to flea and mite assemblages (a) within a host species across regions (component metacommunities; CtM; *dda*_*site*_ = *dda*_*region*_) and (b) within a region across host species (compound metacommunities; CdM, *dda*_*site*_ = *dda*_*host*_). In CtMs, *dda*_*sp*_ and *dda*_*region*_ equally contributed to DD, whereas the relative contributions of *dda*_*sp*_ and *dda*_*host*_ to DD in CdMs varied from the former being higher than the latter and vice versa. In CtM and CdM, *dda*_*sp*_ increased in low-abundance ectoparasites exploiting a restricted number of hosts. In CtMs, *dda*_*region*_ was associated with the regional environment, but we failed to find host traits affecting *dda*_*host*_ in CdMs. We conclude that ectoparasite species and either regions in CtMs or host species in CdMs independently contribute to DD.

**Supplementary Information:**

The online version contains supplementary material available at 10.1007/s00436-024-08408-6.

## Introduction

A regional “habitat-specific species pool” is determined as a set of species occurring in a region that can potentially inhabit any within-region locality because of appropriate ecological conditions for a given taxon (Cornell and Harrison 2014; de Bello et al. 2016; Zobel 2016). The differences in the processes determining species richness and composition between a regional species pool and a local species set lead to (a) the latter being always smaller than the former and (b) differences in species richness between localities within a region. This is because a regional species pool results from large-scale evolutionary, historical, and biogeographic processes, whereas a local species assemblage is determined by small-scale ecological, demographic, and stochastic processes (Pärtel et al. 1996, 2011, 2013; Fløjgaard et al. 2020). Therefore, the species set in a given locality is represented by (a) species that are actually present in the locality and (b) species from the regional pool that could potentially inhabit this locality (ecological conditions are suitable) but that are actually absent. This latter portion of local species diversity is defined as dark diversity (Pärtel et al. 2011), which, obviously, cannot be observed and measured but can be estimated.

The dark diversity concept was originally proposed for conservation purposes, aiming to identify the areas requiring special attention because of substantial differences between the local and regional diversity (i.e., high dark diversity) (e.g., Lewis et al. 2017; Brown et al. 2018; Morel et al. 2022). In addition, the dark diversity approach has led to a better understanding of the historical dynamics of biodiversity (Trindade et al. 2020) and the ecology of commonness and rarity (Riva and Mammola 2021). Dark diversity estimations have been applied to a variety of free-living fungal, plant, and animal taxa (Riibak et al. 2015; Lewis et al. 2017; Moeslund et al. 2017; Boussarie et al. 2018; Estrada et al. 2018; Pärtel et al. 2017, 2019; Fernandes et al. 2019; Fløjgaard et al. 2020), as well as to parasite-host associations (Krasnov et al. 2022a, b, c; Junker et al. 2023). The main aims of using dark diversity for parasites and their hosts were (a) to compare a parasite’s dark diversity between localities or host species and (b) to identify missing links in parasite-host networks. Estimations of dark diversity in parasite-host associations would identify parasites that are expected in a region or host species but have not been detected and, thus, would allow better predictions of parasitic disease outbreaks.

Krasnov et al. (2022a) studied the dark diversity of flea assemblages and found that the dark diversity of fleas parasitic on the same host species, across regions, was mainly affected by the amount of green vegetation in a region, whereas the dark diversity of flea assemblages of different host species, within a region, was mainly affected by the degree of host sociality and its shelter structure. This study, however, did not consider traits of individual flea species but rather focused on “site”-related drivers of flea dark diversity (a region or a host species was considered as a site). Therefore, it remained unclear whether flea dark diversity might be driven by their traits and which flea species are highly likely to be a part of dark diversity. In fact, a species may be absent from a site because its traits somehow prevent its presence in this site, regardless of the site features.

To disentangle the species- and site-driven mechanisms of dark diversity, Fujinuma and Pärtel (2023) have advanced the dark diversity approach and proposed a novel metric, dark diversity affinity (DDA). DDA measures the tendencies of (a) species to be absent from sites that they could potentially inhabit and (b) sites to lack species that could potentially be present there. Therefore, DDA can be further decomposed into individual species DDA (*dda*_*sp*_) and individual site DDA (*dda*_*site*_). Fujinuma and Pärtel (2023) developed a Bayesian model that encompasses a presence-absence species × site matrix, a species × site matrix of suitability (see below), species traits, and site attributes. Application of this model (a so-called “species-site unified model”) allows relating species traits and site attributes (e.g., environmental variables) to DDA and, thus, distinguishing between the species-driven and the site-driven processes determining dark diversity.

Here, we applied the concept of DDA and its decomposition into *dda*_*sp*_ and *dda*_*site*_ to assemblages of ectoparasites (fleas and gamasid mites) harboured by small mammalian hosts in the Palearctic (approximately the same data as in Krasnov et al. 2022a, c). We applied the species-site unified model to parasite assemblages within a host species across regions (= species-region unified model) and to parasite assemblages harboured by different host species within a region (= species-host unified model). In parasitological terminology, the former represents a set of parasite component communities (= ensembles of all parasite species exploiting a host population), whereas the latter represents a parasite compound community (= an ensemble of all parasites exploiting a host community) (Holmes and Price 1986). Given the definition of a metacommunity as a set of ecological communities at different sites potentially, but not necessarily, linked by dispersal (Leibold and Mikkelson 2002), a set of component communities of the same host species across locations can thus be termed a component metacommunity. If a compound community is considered as a set of component communities of different host species, then it can be termed a compound metacommunity (Dallas and Presley 2014). For a component metacommunity, all flea or mite species exploiting a given host across its geographic range represent a host-specific species pool because all of them are able to exploit this host. For a compound metacommunity, all flea or mite species exploiting all hosts in a given region represent a region-specific species pool because all of them are able to persist in this region. Consequently, “sites” for component metacommunities in our study are represented by different regions, whereas “sites” for compound communities are represented by different host species.

The aims of this study were threefold. First, we asked whether the dark diversity of ectoparasite component and compound metacommunities is mainly species- or site-driven (site = region for component metacommunities, and site = host species for compound metacommunities). Second, we asked whether the relative importance of species-related and site-related processes determining dark diversity affinity differs between component and compound metacommunities. Finally, we asked which flea and mite species traits, region characteristics, and host species traits determine their DDA.

## Materials and methods

### Data on flea and mite distributions

Data on flea and gamasid mite (obligatory or facultatively haematophagous species only) distributions were taken from published surveys that reported ectoparasite counts recorded on a given number of individuals of each small mammal species (Rodentia, Eulipotyphla, and Ochotonidae) in 47 (fleas) and 29 (mites) regions of the Palearctic (see maps in Supplementary Figures S1–S2 and the lists of regions in Supplementary Tables S1–S2). The details on sampling procedures can be found in the respective publications (see references in Krasnov et al. 2009, 2015). The input data for the DDA model (see below) is a presence-absence species × site matrix (Fujinuma and Pärtel 2023). Consequently, we selected for the analyses those host species within a region for which at least 15 individuals were parasitologically examined. Using a lower number of host individuals would underestimate parasite presences and overestimate parasite absences because of the aggregated distribution of parasites among host individuals (e.g., Shaw and Dobson 1995). Then, for the component metacommunity analyses, we selected host species that occurred in at least 15 (for fleas) and 12 (for mites) regions. This resulted in the selection of seven host species for fleas [*Apodemus agrarius* (Pallas, 1771), *Apodemus uralensis* (Pallas, 1811), *Cricetulus migratorius* (Pallas, 1773), *Microtus arvalis* (Pallas, 1778), *Microtus oeconomus* (Pallas, 1776), *Myodes rutilus* (Pallas, 1779), and *Sorex araneus* L.] and five host species for mites [*Craseomys rufocanus* (Sundevall. 1846), *M. oeconomus*, *Myodes glareolus* (Schreber, 1780), *M. rutilus*, and *S. araneus*] (see details in Supplementary Table S3). For the compound metacommunity analyses, we selected regions where fleas and mites were recorded on at least 14 (for fleas) and 11 (for mites) host species. This resulted in the selection of eight regions for fleas (Altai Mountains, Armenia, Dzungarian Alatau, Kurgan, Poland, Tomsk-Tyumen, Turkmenistan, and Tatarstan) and six regions for mites (Chulym River, Krasnodar, Northern Russian Far East, Novosibirsk, Omsk Forest-Steppe Zone, and Southern Russian Far East) (see maps in Supplementary Figures S1–S2, descriptions in Supplementary Tables S1–S2, and details in Supplementary Table S3). The difference in data selection criteria between fleas and mites was merely because our database for fleas was twice as large as that for mites.

## Parasite species traits

Fleas and mites were characterized by five quantitative and either two (fleas) or one (mites) categorical traits. Quantitative traits were: (a) characteristic mean abundance on the principal host; (b-c) the degree of host specificity in terms of the numbers and phylogenetic diversity of hosts exploited by a parasite across its geographic range; (d) body size; and (e) the degree of sexual dimorphism (see explanations and references in Supplementary Text S1). Categorical trait variables for fleas included (a) microhabitat preference (spending most of their time either on the host’s body or in its burrow/nest, or no clear preference) and (b) the occurrence and/or number of sclerotized combs (ctenidia) that allow a flea to anchor itself in a host’s hair (either no combs or one or two combs) (see Krasnov 2008). Mite species were characterized by their feeding mode as being either (a) obligatory exclusively haematophageous (feeding solely on the host’s blood), (b) obligatory non-exclusively haematophageous (feeding on both the host’s blood and small nidicolous arthropods), or (c) facultatively haematophageous (see Radovsky 1985).

## Host species traits

Small mammals were characterized by 13 traits presumably affecting the patterns of parasitism by nidicolous arthropod ectoparasites such as fleas and gamasid mites (e.g., Krasnov et al. 2016). These were four quantitative and nine categorical traits. Quantitative traits were (a) average body mass; (b) relative brain mass; (c) dispersal range (the distance between the birth and the breeding locations); and (d) geographic range size. Categorical traits were (a) nest location (on, above, or below ground); (b) life style (ground-dwelling, fossorial, arboreal, or semi-aquatic); (c) diel activity (diurnal, cathemeral, or nocturnal); (d) feeding habits (omnivorous, folivorous, granivorous, insectivorous, granivorous-folivorous, or granivorous-insectivorous); (e) occurrence of hibernation or torpor; (f) sociality (solitary or social); (g) habitat breadth (one to six, according to level 1 IUCN habitats); (h) shelter depth (shallow, intermediate, or deep); and (i) shelter complexity (simple, intermediate, or complex). The rationale behind the selection of these traits and sources of information can be found in Supplementary Text S2 and elsewhere (Krasnov et al. 2016, 2019).

## Regional environment (environmental variables)

Each region was characterized by two climatic variables (air temperature and precipitation), one vegetation variable (Normalized Difference Vegetation Index; NDVI), one geomorphological variable (mean altitude), regional area, and the species richness of available host species. Data on climatic and vegetation variables, as well as on available host species richness, were taken from our earlier study (Krasnov et al. 2022b). In brief, we applied principal component analyses (PCA) to data on mean, maximal, and minimal air temperature, seasonal precipitation, and NDVI (separately for regions where fleas and mites were surveyed), and we substituted the original values with the scores of the first principal components (PCs) produced by the PCA of each environmental category (see details in Krasnov et al. 2022b). All resulting PCs correlated positively with the original variables. Data on the species richness of available hosts were controlled for unequal sampling effort and sampling area (see Krasnov et al. 2022b). Mean altitude and regional area were calculated using ArcGIS 10.6.

## Model

In this section, we closely follow the terminology of Fujinuma and Pärtel (2023). As mentioned above, they introduced a novel metric, dark diversity affinity (DDA), that simultaneously measures the tendency of species to belong to dark diversity (to be absent from suitable sites) and the tendency of sites to support dark diversity (to lack suitable species). Suitability (*suit* as in Fujinuma and Pärtel 2023) estimates the ecological suitability of a given site for a given species, independently of whether this species is present in or absent from this site. Suitability is calculated as a probability of species occurrence in a site based on pairwise co-occurrence data by comparing the realized co-occurrence pattern of each species pair to that expected if there is no association between these species (calculated as the mean value of the hypergeometric distribution), and the extent of the departure of the observed co-occurrence between the species pair from a random association is used as the indicator value for this pair (see details in Carmona and Pärtel 2021). Consequently, *suit* may take values from 0 to 1.

DDA also ranges from zero to unity, with a value of 0.5 being the threshold; thus, DDA > 0.5 indicates that a species is likely to be absent from a site, whereas DDA < 0.5 indicates that a species is likely to be present in a site. As mentioned above, DDA, for each species-site combination, is further decomposed into the dark diversity affinity of a species (*dda*_*sp*_) and the dark diversity affinity of a site (*dda*_*site*_), which follow the same direction as DDA. In other words, a species having high *dda*_*sp*_ likely belongs to dark diversity (is mostly absent from suitable sites), while a site having high *dda*_*site*_ likely supports dark diversity (i.e., suitable species are mostly absent from it). Assuming that the values of *dda*_*sp*_ and *dda*_*site*_ are associated with species traits and site characteristics (e.g., environmental variables), respectively, they can be modelled as logistic regressions (with the response binary variable being presence-absence expectation) for each species-site combination, that is

$$\mathrm{logit}({{dda}}_{{sp}})=\mathrm a+{\mathrm b}_{1.{sp}}{}^\ast\mathrm T1+{\mathrm b}_{2.{sp}}{}^\ast\mathrm T2+{\mathrm b}_{3.{sp}(\mathrm{level}1)}{}^\ast\mathrm T3+{\mathrm b}_{3.{sp}(\mathrm{level}2)}{}^\ast\mathrm T3\;\mathrm{and}\;\mathrm{logit}({{dda}}_{{site}})=\mathrm a+{\mathrm b}_{1.{site}}{}^\ast\mathrm E1+{\mathrm b}_{2.{site}}{}^\ast\mathrm E2+{\mathrm b}_{3.{site}(\mathrm{level}1)}{}^\ast\mathrm E3+{\mathrm b}_{3.{site}(\mathrm{level}2)}{}^\ast\mathrm E3$$

respectively. Here, *a* and *b* are coefficients of the model, and T1-T3 and E1-E3 are independent variables (i.e., species traits and site characteristics, respectively). For quantitative independent variables (T1-T2 and E1-E2), a positive value of *b* would indicate an increase in *dda* (i.e., an increase in the probability of this species to be absent from this site or in the probability of this site to lack this species). For categorical variables (T3 and E3 with, say, two levels), *b* is the deviation from the intercept of a model for each level. Then, *dda*_*sp*_ and *dda*_*site*_ are joined into a unified *DDA* (in *italics* as in Fujinuma and Pärtel 2023) that represents site-specific dark diversity affinity for the presence or absence of a given species and is calculated for each species-site combination as the mean of these two metrics, namely

$$\mathrm{logit}\;({DDA})=\lbrack\mathrm{logit}\;({{dda}}_{{sp}})+\mathrm{logit}\;({{dda}}_{{site}})/2\rbrack.\;$$

The logit functions result in the three metrics (*DDA*, *dda*_*sp*_ and *dda*_*site*_) being in the range of 0–1.

The presence likelihood (*p*) of each species in each site is predicted via site-specific suitability adjusted by *DDA* as

$$\mathrm{logit}\;(p)=\mathrm{logit}\;\lbrack(1-{DDA}\rbrack^\ast\mathrm{suit}\rbrack+\mathrm\delta,$$

where δ is the constant unique for each metacommunity used to balance the level of *p* to the observed presence/absence pattern (*prab*). δ is obtained as

$$\mathrm{logit}\lbrack\mathrm{average}(prab)\rbrack-\mathrm{logit}\lbrack0.5^\ast\mathrm{average}(suit)\rbrack$$

Here, *prab* is merely a vector of observed presences (1) and absences (0) for a metacommunity matrix, and 0.5 (for *DDA* and *dda*) is established as a threshold at a given suitability below which a species is expected to be more present in and above which a species is expected to be more absent from sites with a given suitability.

Finally, the observed presence/absence pattern (*prab*) is linked to the presence likelihood (*p*), assuming a Bernoulli distribution of the latter as [*prab* ~ Bern(*p*)] for each species-site combination. This allows linking species and site characteristics to an unexpected presence/absence pattern at a given suitability (see Fujinuma and Pärtel 2023 for details).

## Data analyses

We calculated suitability (*suit*), δ, and presence likelihood (*p*) from the input data. Suitability was calculated using the “DarkDiv” function of the “DarkDiv” package (Carmona and Pärtel 2020), implemented in the R Statistical Environment (R Core Team 2024). Presence likelihood and δ were calculated using the equations taken from Fujinuma and Pärtel (2023), specified in the previous subsection. Then, the parameters of the species-site unified models for each component and compound metacommunity were estimated using the Bayesian model developed by Fujinuma and Pärtel (2023) and applying their R code. Specifically, we estimated *DDA* (unified), *dda* for species and sites, and *a* and *b* coefficients of the logistic models. In our application, *dda*_*sp*_ values were left as they are in the approach of Fujinuma and Pärtel (2023), whereas the label of Fujinuma and Pärtel’s (2023) *dda*_*site*_ was modified to *dda*_*region*_ for component metacommunities and *dda*_*host*_ for compound metacommunities. The prior distributions were established following Fujinuma and Pärtel (2023), namely setting 0.5 as a default to *a* parameters, 2.5 to *b* parameters, and 0 as the mean of all prior distributions (see rationale and explanations in Fujinuma and Pärtel 2023). Before running the models, all quantitative variables (ectoparasite and host traits, as well as environmental variables) were standardized to a mean of 0 and a standard deviation of 0.5.

The models were fitted using Gibbs sampler JAGS 4.3.1 implemented in the R package “rjags” (Plummer 2024). Following Fujinuma and Pärtel (2023), the number of Markov Chain Monte Carlo (MCMC) chains was set at 3, and the number of posterior samplings per chain was set at 333 for each metacommunity. After each run, we estimated convergence via the Gelman–Rubin statistic *R-hat* ≤ 1.1 (Gelman and Rubin 1992), using the R function developed by Fujinuma and Pärtel (2023). If the run of a model returned *R-hat* > 1.1, we adjusted burn-in and sampling iterations and the thinning interval of posterior samplings whenever necessary. For all component metacommunities, the number of burn-in and sampling iterations was established at 4000, with a thinning interval of 12. For compound metacommunities, the numbers of burn-in and sampling iterations varied from 4000 to 25000, with the thinning intervals ranging from 12 to 75.

Partly following Fujinuma and Pärtel (2023), we divided each component or compound metacommunity matrix into two species-site sets, namely those that were characterized by absence (further referred to as absent subsets) and those characterized by presence (further referred to as present subsets). Then, we tested for differences in the probability likelihood, suitability, *DDA*, *dda*_*sp*_, and *dda*_*region*_ (for component metacommunities) or *dda*_*host*_ (for compound metacommunities) between the two subsets using Kruskal–Wallis ANOVAs.

## Results

**Fig. 1:**
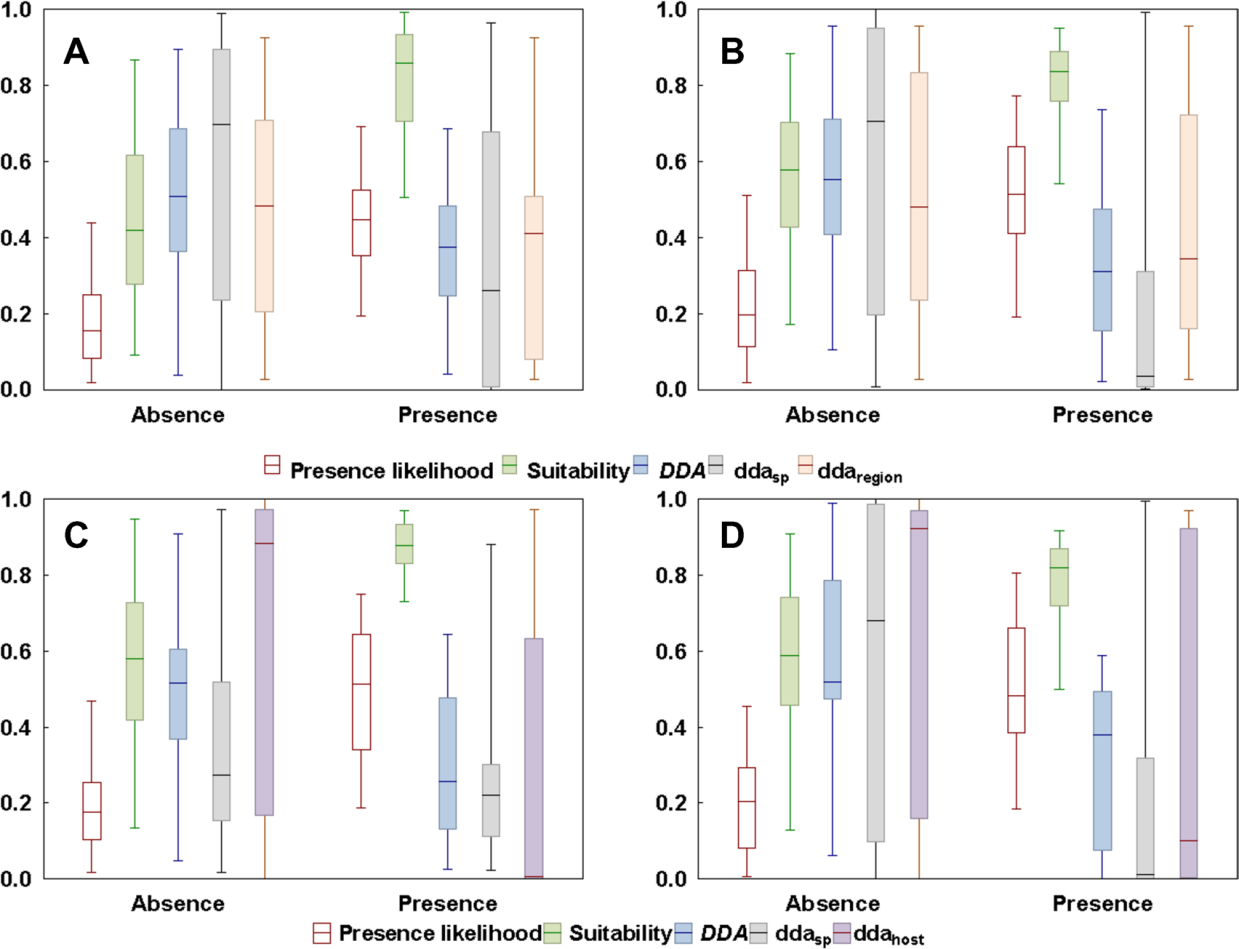
Estimated parameter distributions of the species-site (i.e., species-region for component metacommunities and species-host for compound metacommunities) unified model in the absent and the present subsets of region-species (for component metacommunities) or host-species (for compound metacommunities). Whiskers: 2.5% and 97.5% percentiles; box: 25% and 75% percentiles; middle line: median. Each estimate was taken as the median value of 999 Bayesian posterior samplings. **A**: flea component metacommunity of *Myodes rutilus*, **B**: mite component metacommunity of *Myodes rutilus*, **C**: flea compound community in Armenia, **D**: mite compound community in the Northern Russian Far East

In all component and compound metacommunities of both fleas and mites, the absent subsets were characterized by significantly higher DDA and, concomitantly, lower presence likelihood and suitability than the present subsets (Tables 1–2). In the majority of component metacommunities, the contributions of *dda*_*sp*_ to the unified *DDA* of the absent subsets were either higher than or almost equal to that of *dda*_*region*_ (Table 1). On the contrary, the contributions of *dda*_*sp*_ to the unified *DDA* in the present subsets were mostly lower than those of *dda*_*region*_ (Table 1). In the absent subsets of compound metacommunities, the relative contributions of *dda*_*sp*_ and *dda*_*host*_ to the unified *DDA* varied, with the former being higher than the latter in five of eight regions for fleas and three of six regions for mites, whereas the opposite was true in the remaining regions (Table 2). Illustrative examples of the relationships between model parameters for component and compound metacommunities of fleas and mites are presented in Fig. 1.

**Table 1:** The results of Kuskal-Wallis ANOVAs for differences in presence likelihood (*p*), suitability (suit), *DDA*, *dda*_*sp*_, and *dda*_*region*_ between absent (AS) and present (PS) species-site subsets (see text for explanations) in flea and mite component metacommunities. *DDA*, *dda*_*sp*_, and *dda*_*region*_ each represents 999 Bayesian posterior samplings. All differences are significant (*p* < 0.05)

Parasite	Host species	Parameter	Median		*H*
			AS	PS	
Fleas	*Apodemus agrarius*	Presence likelihood	0.12	0.44	295.35
		Suitability	0.35	0.90	325.80
		*DDA*	0.49	0.39	50.73
		*dda*_*sp*_	0.47	0.14	50.80
		*dda*_*region*_	0.49	0.48	5.46
	*Apodemus uralensis*	Presence likelihood	0.11	0.38	332.59
		Suitability	0.42	0.92	364.58
		*DDA*	0.52	0.40	75.79
		*dda*_*sp*_	0.68	0.21	43.11
		*dda*_*region*_	0.55	0.27	23.52
	*Cricetulus migratorius*	Presence likelihood	0.11	0.40	328.68
		Suitability	0.38	0.95	362.50
		*DDA*	0.45	0.34	36.03
		*dda*_*sp*_	0.36	0.23	15.38
		*dda*_*region*_	0.40	0.23	24.22
	*Microtus arvalis*	Presence likelihood	0.13	0.44	397.28
		Suitability	0.43	0.85	388.38
		*DDA*	0.54	0.33	121.95
		*dda*_*sp*_	0.81	0.03	121.71
		*dda*_*region*_	0.49	0.46	11.79
	*Microtus oeconomus*	Presence likelihood	0.18	0.54	184.53
		Suitability	0.40	0.82	184.30
		*DDA*	0.49	0.30	48.99
		*dda*_*sp*_	0.56	0.15	33.72
		*dda*_*region*_	0.44	0.26	14.44
	*Myodes rutilus*	Presence likelihood	0.15	0.45	394.33
		Suitability	0.42	0.86	368.12
		*DDA*	0.51	0.37	75.84
		*dda*_*sp*_	0.70	0.26	61.38
		*dda*_*region*_	0.48	0.41	11.58
	*Sorex araneus*	Presence likelihood	0.14	0.43	261.22
		Suitability	0.55	0.89	300.19
		*DDA*	0.54	0.35	113.40
		*dda*_*sp*_	0.67	0.12	101.40
		*dda*_*region*_	0.59	0.46	36.05
	*Craseomys rufocanus*	Presence likelihood	0.21	0.46	106.63
		Suitability	0.65	0.69	14.99
		*DDA*	0.61	0.37	95.06
		*dda*_*sp*_	0.91	0.01	88.01
		*dda*_*region*_	0.75	0.38	20.97
	*Microtus oeconomus*	Presence likelihood	0.27	0.47	137.57
		Suitability	0.58	0.61	15.58
		*DDA*	0.59	0.34	112.93
		*dda*_*sp*_	0.88	0.04	93.70
		*dda*_*region*_	0.60	0.36	11.33
	*Myodes glareolus*	Presence likelihood	0.24	0.47	109.73
		Suitability	0.51	0.77	67.82
		*DDA*	0.54	0.32	68.49
		*dda*_*sp*_	0.67	0.03	51.14
		*dda*_*region*_	0.49	0.32	8.30
	*Myodes rutilus*	Presence likelihood	0.20	0.51	246.75
		Suitability	0.58	0.84	212.16
		*DDA*	0.55	0.31	113.01
		*dda*_*sp*_	0.71	0.03	115.01
		*dda*_*region*_	0.48	0.34	14.91
	*Sorex araneus*	Presence likelihood	0.14	0.40	230.93
		Suitability	0.56	0.80	203.69
		*DDA*	0.65	0.38	134.80
		*dda*_*sp*_	0.81	0.02	137.60
		*dda*_*region*_	0.72	0.56	17.71

**Table 2:** The results of Kuskal-Wallis ANOVAs for differences in presence likelihood (*p*), suitability (suit), *DDA*, *dda*_*sp*_, and *dda*_*host*_ between absent (AS) and present (PS) species-site subsets (see text for explanations) in flea and mite compound metacommunities. *DDA*, *dda*_*sp*_, and *dda*_*host*_ each represents 999 Bayesian posterior samplings. All differences, except those denoted by *, are significant (*p* < 0.05)

Parasite	Region	Parameter	Median		*H*
			AS	PS	
Fleas	Altai Mountains	Presence likelihood	0.67	0.83	31.31
		Suitability	0.71	0.75	0.003*
		*DDA*	0.58	0.42	52.50
		*dda*_*sp*_	1.00	0.39	27.07
		*dda*_*host*_	0.42	0.001	29.93
	Armenia	Presence likelihood	0.18	0.51	208.60
		Suitability	0.58	0.88	195.71
		*DDA*	0.52	0.26	72.74
		*dda*_*sp*_	0.27	0.22	8.80
		*dda*_*host*_	0.88	0.01	61.48
	Dzungarian Alatau	Presence likelihood	0.24	0.53	149.91
		Suitability	0.56	0.89	99.39
		*DDA*	0.50	0.37	37.77
		*dda*_*sp*_	0.36	0.31	2.01*
		*dda*_*host*_	0.51	0.03	51.19
	Kurgan	Presence likelihood	0.37	0.57	93.49
		Suitability	0.61	0.67	12.94
		*DDA*	0.51	0.31	64.86
		*dda*_*sp*_	0.94	0.01	52.37
		*dda*_*host*_	0.70	0.02	18.31
	Poland	Presence likelihood	0.40	0.71	167.34
		Suitability	0.82	0.94	90.45
		*DDA*	0.57	0.28	148.99
		*dda*_*sp*_	0.77	0.21	45.20
		*dda*_*host*_	0.95	0.06	104.42
	Tomsk-Tyumen	Presence likelihood	0.26	0.50	218.64
		Suitability	0.71	0.85	98.39
		*DDA*	0.54	0.29	138.44
		*dda*_*sp*_	0.91	0.02	85.76
		*dda*_*host*_	0.84	0.09	53.10
	Turkmenistan	Presence likelihood	0.14	0.57	244.14
		Suitability	0.58	0.97	194.98
		*DDA*	0.61	0.42	110.69
		*dda*_*sp*_	0.79	0.76	7.04
		*dda*_*host*_	0.73	0.01	110.70
	Tatarstan	Presence likelihood	0.18	0.66	279.24
		Suitability	0.68	0.97	258.57
		*DDA*	0.54	0.39	107.67
		*dda*_*sp*_	0.73	0.08	80.12
		*dda*_*host*_	0.71	0.08	19.90
Mites	Chulym River	Presence likelihood	0.25	0.57	81.42
		Suitability	0.57	0.77	34.14
		*DDA*	0.50	0.26	44.85
		*dda*_*sp*_	0.87	0.16	21.69
		*dda*_*host*_	0.77	0.001	22.01
	Krasnodar	Presence likelihood	0.18	0.42	128.35
		Suitability	0.57	0.87	131.10
		*DDA*	0.50	0.43	62.85
		*dda*_*sp*_	0.99	0.24	39.69
		*dda*_*host*_	0.03	0.001	16.26
	Northern Russian Far East	Presence likelihood	0.20	0.48	78.12
		Suitability	0.59	0.82	55.46
		*DDA*	0.52	0.38	44.93
		*dda*_*sp*_	0.68	0.01	28.82
		*dda*_*host*_	0.92	0.10	19.62
	Novosibirsk	Presence likelihood	0.30	0.64	168.08
		Suitability	0.71	0.75	17.81
		*DDA*	0.74	0.40	174.50
		*dda*_*sp*_	0.96	0.01	131.89
		*dda*_*host*_	0.91	0.30	40.19
	Omsk Forest-Steppe Zone	Presence likelihood	0.32	0.58	172.52
		Suitability	0.69	0.78	28.66
		*DDA*	0.60	0.38	143.43
		*dda*_*sp*_	0.71	0.03	94.99
		*dda*_*host*_	0.90	0.55	50.72
	Southern Russian Far East	Presence likelihood	0.23	0.59	172.18
		Suitability	0.64	0.89	164.05
		*DDA*	0.56	0.31	108.81
		*dda*_*sp*_	0.56	0.08	49.79
		*dda*_*host*_	0.94	0.47	54.39

**Fig. 2:**
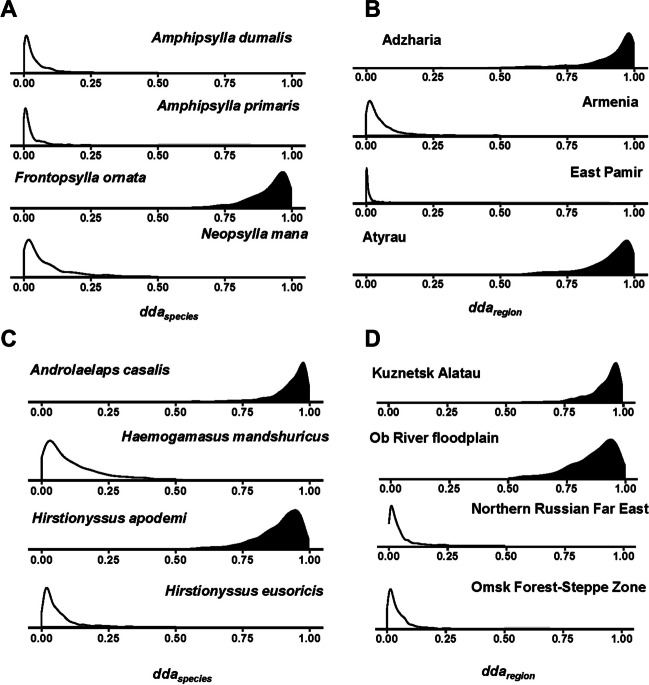
Posterior distributions of individual dark diversity affinity for species (*dda*_*sp*_) and regions (*dda*_*region*_) estimated by the species-region unified model for the flea component metacommunity of *C. migratorius* (**A**, **B**) and the mite component metacommunity of *M. rutilus* (**C**, **D**). Black: density distribution is significantly larger than 0.5; white: density distribution is significantly smaller than 0.5 (based on a 95% credible interval)

In flea component metacommunities, the proportion of species demonstrating a tendency to either absence or presence varied from 0.16 in *C. migratorius* to 0.66 in *M. arvalis*, with the proportions of fleas tending to be absent varying from 0.01 to 0.34 in these hosts, respectively (see detailed results in Supplementary Tables S4–S10). In two of seven flea component metacommunities (*A. agrarius* and *M. oeconomus*), none of the regions demonstrated a tendency to lack suitable species, whereas the number of regions with their *dda* indicating dark diversity varied from one to four in the remaining metacommunities (see detailed results in Supplementary Table S11). Illustrative examples of the density distributions of *dda*_*sp*_ and *dda*_*region*_ for the component metacommunity of *C. migratorius* are presented in Fig. 2A, B.

The proportion of mite species in component metacommunities with a significant absence/presence tendency was higher than that of fleas (0.50–0.81), with an almost equal number of species tending to be absent or present (see detailed results in Supplementary Tables S12–S13). No region lacking suitable mites exploiting *M. glareolus* was detected, whereas one to two regions that lacked suitable species were found in the remaining component metacommunities (see detailed results in Supplementary Table S14 and an illustrative example of *dda*_*sp*_ and *dda*_*region*_ density distributions in Fig. 2C, D for the component metacommunity of *M. rutilus*).

**Fig. 3:**
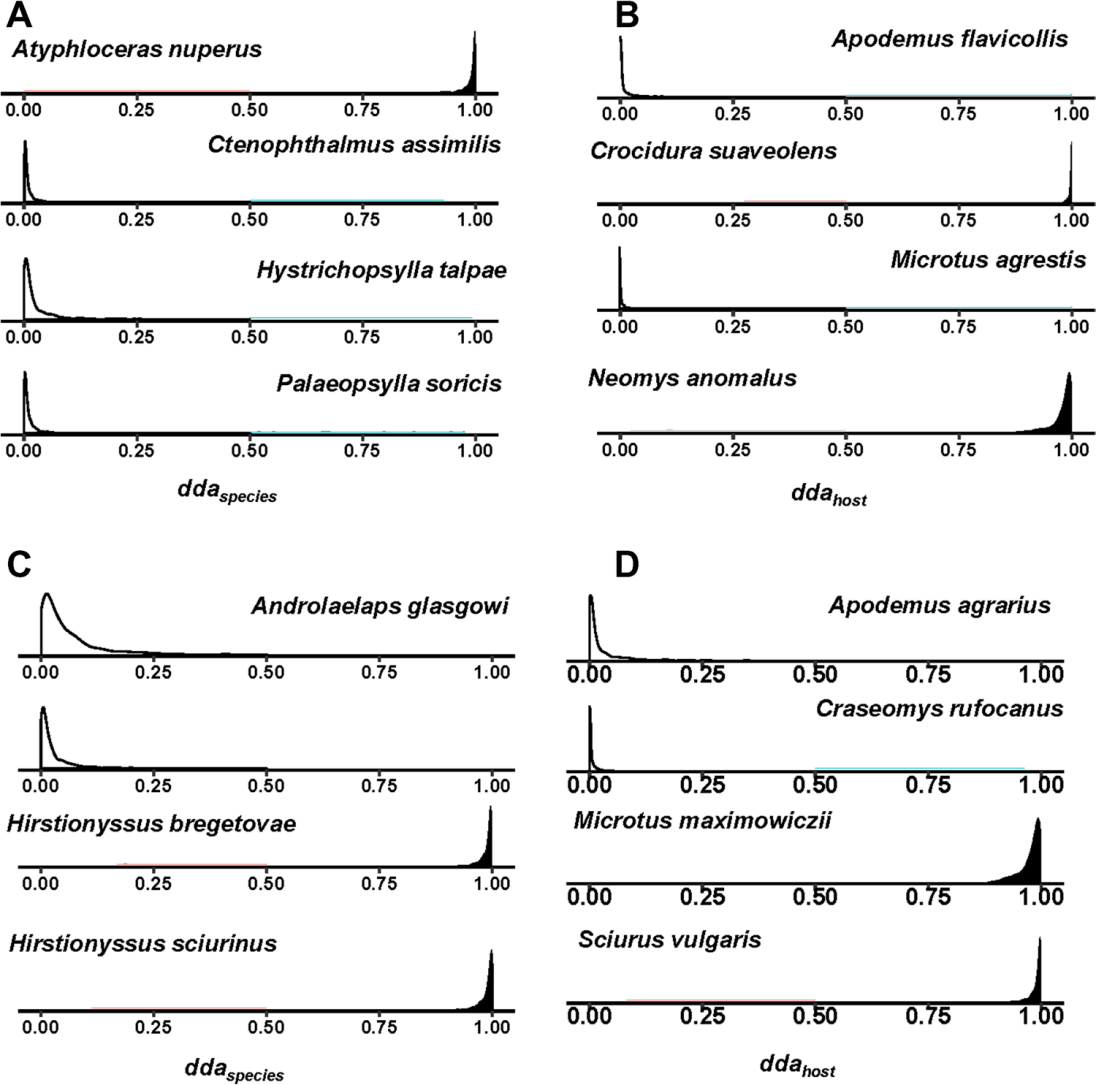
Posterior distributions of individual dark diversity affinity for species (*dda*_*sp*_) and hosts (*dda*_*host*_) estimated by the species-host unified model for a flea compound metacommunity in Poland (**A**, **B**) and a mite compound metacommunity in the Southern Russian Far East (**C**, **D**). Black: density distribution is significantly larger than 0.5; white: density distribution is significantly smaller than 0.5 (based on a 95% credible interval)

In compound metacommunities, the relative numbers of both flea and mite species that demonstrated significant density distributions of their *dda*_*sp*_ varied from 0 to 0.52 for fleas and from 0 to 0.71 for mites (see detailed results in Supplementary Tables S15–16 and S18–19, respectively). The proportions of fleas and mites tending to be absent from suitable hosts varied from 0 to 0.32 and 0.33, respectively. The proportion of host species that fleas and mites could potentially exploit but, in fact, did not (i.e., having *dda*_*host*_ significantly > 0.5) varied from 0 to 0.27 for fleas and from 0.06 to 0.21 for mites (see detailed results in Supplementary Tables S17 for fleas and S20 for mites). Illustrative examples of the density distributions of *dda*_*sp*_ and *dda*_*host*_ for a flea compound metacommunity in Poland and a mite compound metacommunity in the Southern Russian Far East are presented in Fig. 3.

Species traits and region characteristics determining *dda*_*sp*_ and *dda*_*region*_, respectively, in component metacommunities are presented in Table 3. *dda*_*sp*_ was mostly associated with a characteristic abundance of parasite species and their host specificity, in terms of the number of host species exploited or their phylogenetic diversity, although the latter factor mostly affected the *dda*_*sp*_ of mites and not of fleas. In general, the probability to belong to dark diversity increased in low-abundance species exploiting a restricted number of hosts (see detailed results in Supplementary Table S21). The correlation of *dda*_*sp*_ with the phylogenetic diversity of a host spectrum was mostly positive in mite component metacommunities. However, fleas of *M. rutilus* demonstrated somewhat different trends, with their *dda*_*sp*_ increasing with an increase in abundance and host phylogenetic diversity but still decreasing with an increase in the size of a host spectrum. The remaining ectoparasite traits played minor roles in determining *dda*_*sp*_. All regional characteristics (except area) were found to affect the *dda*_*region*_ of component metacommunities (see detailed results in Supplementary Table S22). On the contrary, the effect of the number of available host species on *dda*_*region*_ was consistent among metacommunities. Whenever this effect was found, it indicated an increase in dark diversity with a decrease in available host species richness. In addition, no significant relationships between environmental variables and *dda*_*region*_ were found for flea metacommunities of *M. oeconomus* and mite metacommunities of *M. glareolus*, likely because in each of these metacommunities, significant *dda*_*region*_ was detected for one region only.

**Table 3:** Summary of logistic regression models for *dda*_*sp*_ and *dda*_*region*_ in the species-site unified model for flea and mite component metacommunities. Each coefficient represents the mean value of 999 Bayesian posterior samplings. Only significant coefficients are shown. Significance was based on a 95% credible interval (2.5%–97.5%). Species traits for *dda*_*sp*_ are as follows. Ab: characteristic abundance; HN: number of host species across geographic range; PD: phylogenetic diversity of these hosts; BS: body size; SD: sexual size dimorphism; MHpref (for fleas): microhabitat preference ([1]: host’s hair; [2]: no clear preference); Combs: possession and number of sclerotized combs (for fleas) ([2]: one comb; [3]: two combs); Feed (for mites): feeding mode (for mites) ([2]). Environmental variables for *dda*_*region*_ are as follows. Alt: mean altitude; T: air temperature (the first principal component; see text for explanation); P: precipitation (the first principal component; see text for explanation); NDVI: normalized difference vegetation index (the first principal component; see text for explanation); Area: area of a region; HSR: number of available host species

Parasite	Host species	*dda*	Equation: logit (*dda*) =
Fleas	*Apodemus agrarius*	*dda*_*sp*_	-4.7*HN + 1.26*Combs[2]
		*dda*_*region*_	4.94*NDVI
	*Apodemus uralensis*	*dda*_*sp*_	- 4.92*HN
		*dda*_*region*_	-3.09*Alt + 2 .57*T—2.81*P
	*Cricetulus migratirius*	*dda*_*sp*_	-2.11*Ab—1.81*SD + 1.49*MHpref[1]
		*dda*_*region*_	-7.73*Alt—4.41*T—3.83*HSR
	*Microtus arvalis*	*dda*_*sp*_	-2.12*Ab—6.41*HN—3.08*BS—1.42*SD
		*dda*_*region*_	-3.31*T—2.76*P -5.34*NDVI
	*Microtus oeconomus*	*dda*_*sp*_	-3.19*HN
		*dda*_*region*_	No significant coefficients
	*Myodes rutilus*	*dda*_*sp*_	2.02*Ab—7.27*HN—1.22*PD—1.46*MHpref[2] + 1.08*Combs[2]—1.08*Combs[3]
		*dda*_*region*_	-2.30*Alt + 2.12*P + 2.14*NDVI
	*Sorex araneus*	*dda*_*sp*_	-1.76*Ab—4.96*HN
		*dda*_*region*_	-3.53*HSR
Mites	*Craseomys rufocanus*	*dda*_*sp*_	-7.70*HN + 2.85*PD
		*dda*_*region*_	-2.62*Area—3.55*HSR
	*Microtus oeconomus*	*dda*_*sp*_	-6.99*HN + 3.32*PD + 3.18*Feed[2]—1.76*Feed[3]
		*dda*_*region*_	-2.24*HSR
	*Myodes glareolus*	*dda*_*sp*_	-7.68*Ab—4.01*HN
		*dda*_*region*_	No significant coefficients
	*Myodes rutilus*	*dda*_*sp*_	-5.89*HN + 1.61*Feed[2]
		*dda*_*region*_	-3.31*HSR
	*Sorex araneus*	*dda*_*sp*_	-5.89*HN + 3.75*PD
		*dda*_*region*_	-2.52*Alt—5.48*P + 3.72*NDVI

In compound metacommunities, *dda*_*sp*_ values were associated with approximately the same ectoparasite traits as in component metacommunities (Table 4; see detailed results in Supplementary Table S23). In contrast to mite component metacommunities, the correlation of flea *dda*_*sp*_ with the phylogenetic diversity of a host spectrum in compound metacommunities was mostly negative. In a single flea compound metacommunity (Altai Mountains), no significant coefficients in the model for *dda*_*sp*_ was found, probably due to the extremely low flea species richness in this region (eight species only). In five of eight compound metacommunities of fleas and four of six compound metacommunities of mites, no effect of host traits on *dda*_*host*_ was detected (Table 4, see detailed results in Supplementary Tables S24–S30). In three of the remaining six metacommunities, the role of hosts’ habitat breadth was revealed, with moderate habitat-generalist hosts and habitat-specialist hosts being characterized by relatively lower and relatively higher dark diversity affinity, respectively (Table 4).

**Table 4:** Summary of logistic regression models for *dda*_*sp*_ and *dda*_*host*_ in the species-site unified model for flea and mite compound metacommunities. Each coefficient represents the mean value of 999 Bayesian posterior samplings. Only significant coefficients are shown. Significance was based on based a 95% credible interval (2.5%–97.5%). Species traits for *dda*_*sp*_ are as follows. Ab: characteristic abundance; HN: number of host species across geographic range; PD: phylogenetic diversity of these hosts; BS: body size; MHpref (for fleas): microhabitat preference ([1]: host’s hair; [3]: host’s nest); Combs (for fleas): possession and number of sclerotized combs (for fleas) ([1]: no combs, [2]: one comb; [3]: two combs); Feed (for mites): feeding mode (for mites) ([1]: facultative haematophage, [2]: obligatory exclusive haematophages). Host traits for *dda*_*host*_ are as follows. DISP: dispersal range; HB: habitat breadth ([2] or [3]); ACT: diel activity ([3]: diurnal); SDEP: shelter depth ([2]: intermediate)

Parasite	Region	*dda*	Equation: logit (*dda*) =
Fleas	Altai Mountains	*dda*_*sp*_	No significant coefficients
		*dda*_*host*_	12.12*DISP
	Armenia	*dda*_*sp*_	-2.35*Combs[2]
		*dda*_*host*_	-8.26*HB[3]
	Dzungarian Alatau	*dda*_*sp*_	-9.96*Ab—5.86*PD
		*dda*_*host*_	No significant coefficients
	Kurgan	*dda*_*sp*_	-10.95*Ab—4.61*HN—2.53*MHpref[1]—4.42*Combs[1]
		*dda*_*host*_	2.14*HB[2]
	Poland	*dda*_*sp*_	-4.58*HN
		*dda*_*host*_	No significant coefficients
	Tomsk-Tyumen	*dda*_*sp*_	-5.98*Ab—4.30*PD—2.45*BS—2.83*MHoref[1]—4.13*MHpref[3] + 2.73*Combs[2]—2.73*Combs[3]
		*dda*_*host*_	No significant coefficients
	Turkmenistan	*dda*_*sp*_	-2.20*Ab
		*dda*_*host*_	No significant coefficients
	Tatarstan	*dda*_*sp*_	-7.08*HN—1.77*PD
		*dda*_*host*_	No significant coefficients
Mites	Chulym River	*dda*_*sp*_	-7.45*HN—7.53*BS
		*dda*_*host*_	No significant coefficients
	Krasnodar	*dda*_*sp*_	-9.75*Ab – 6.65*Feed[1]
		*dda*_*host*_	No significant coefficients
	Northern Russian Far East	*dda*_*sp*_	-8.44*HN
		*dda*_*host*_	No significant coefficients
	Novosibirsk	*dda*_*sp*_	-2.96*Ab—12.66*HN—3.89*BS – 2.31*Feed[2]
		*dda*_*host*_	No significant coefficients
	Omsk Forest-Steppe Zone	*dda*_*sp*_	-3.07*Ab – 9.86*HN
		*dda*_*host*_	-7.47*ACT[3] + 4.66*SDEP[2]
	Southern Russian Far East	*dda*_*sp*_	-5.39*HN
		*dda*_*host*_	3.40*HB[2]

## Discussion

In general, the probability of a parasite species to be absent from a locality or a host species (i.e., to belong to dark diversity) is likely higher than that of free-living species due to, at least, two reasons and apart from various random factors. First, parasite individuals are aggregated among host individuals, so the largest proportion of hosts is uninfested (Shaw and Dobson 1995), and the chances to detect parasites in a field study are, thus, relatively low. Second, when an investigator samples parasites, s/he is a second-order sampler because s/he actually samples parasites via sampling hosts, which, in turn, are the real (i.e., first-order) parasite samplers. Third, infestation of a small mammal by ectoparasites, such as fleas, may vary on a daily basis, with a high probability of an individual host to change its infestation status from being infested on one day to being uninfested on the next day and vice versa (Krasnov et al. 2006a).

Our results demonstrated that ectoparasite species and either regions in component metacommunities or host species in compound metacommunities contributed independently to DDA (and, thus, dark diversity), supporting the conclusions of Fujinuma and Pärtel (2023). Fujinuma and Pärtel (2023) proved the independent roles of species DDA and individual site DDA in shaping the dark diversity of nine metacommunities that represented a variety of taxa (from plants to mammals) in a variety of regions (from central Europe to New Zealand and from eastern North America to central South America). In component ectoparasite metacommunities, the contributions of *dda*_*sp*_ and *dda*_*region*_ depended on species traits and the regional environment, respectively. In compound ectoparasite metacommunities, the effect of ectoparasite species traits, *dda*_*sp*_, appeared to be important as well, but we failed to identify host species traits affecting *dda*_*host*_ in many of these metacommunities.

The relative contributions of *dda*_*sp*_ to unified *DDA* were higher than those of *dda*_*region*_ in the majority of component metacommunities. This suggests that dark diversity in these metacommunities was more dependent on *dda*_*sp*_ than on *dda*_*region*_, although both were important. The relative contributions of *dd*_*sp*_ and *dda*_*host*_ in compound metacommunities varied substantially between communities. In some compound metacommunities, unified DDA was mainly regulated by *dda*_*sp*_, whereas in other compound metacommunities, *dda*_*host*_ played a more important role. These differences might be associated with an environmental mediation of parasite-host relationships when the distribution of the same parasites among the same hosts can differ between sites/regions, depending on the regional/local environment (Carney and Dick 2000; Calvete et al. 2004; Krasnov et al. 1998, 2006b; Dallas and Presley 2014).

Abundance and host specificity were among the most important flea and mite species traits determining whether these species belonged to dark diversity. In general, species characterized by relatively low abundance are more likely be part of dark diversity than highly abundant species due to obvious reasons. Indeed, lower parasite abundance often results in lower prevalence, that is, a higher proportion of host individuals that do not harbour parasites (e.g., Poulin and Mouillot 2004). Although parasite abundance is a true parasite species property, it varies to some, albeit low, degree between localities and host species (Arneberg et al. 1997; Krasnov et al. 2006c; Poulin 2006). This variation depends on local conditions being affected by both environmental factors (see below) and host species ecology. For example, parasite abundance often decreases with an increase in host density due to the dilution effect (Cǒté and Poulin 1995; Buck and Lutterschmidt 2017). Parasite abundance may also vary depending on host population composition (e.g., relative numbers of resident and transient individuals; Krasnov et al. 2002).

A positive association between the probability to belong to dark diversity and the degree of host specificity can be expected because host-specific parasites (those with a narrow host range) are usually characterized by low abundance (e.g., Krasnov et al. 2004). Furthermore, host-specific parasites can be absent (a) in component metacommunities, from regions where the abundance of their preferred hosts is low and (b) in compound metacommunities, from non-preferred (albeit suitable to some extent) host species. Interestingly, the effect of host species’ phylogenetic diversity on *dda*_*sp*_ was detected mostly for mites rather than for fleas in component metacommunities and mostly for fleas rather than for mites in compound metacommunities, being positive in the former and negative in the latter. In other words, some determinants of dark diversity affinity are taxon- and scale-dependent. In particular, the phylogenetic host specificity of fleas did not influence their probability of being absent from or present in a given region (i.e., in component metacommunities), but the probability of absence from a certain host species within a region was lower in phylogenetic host specialists than in phylogenetic host opportunists (i.e., in compound metacommunities) because, for example, of this host’s unsuitability. On the contrary, mites exploiting phylogenetically diverse hosts often belonged to regional, but not host-associated, dark diversity. This pattern is difficult to explain. It may be associated with the fact that a proxy of the phylogenetic diversity (taxonomic distinctness) of mite host spectra has been shown to be relatively high in regions with relatively low taxonomic diversity of the mite fauna (Korallo-Vinarskaya et al. 2009). In a mite assemblage with many taxonomically close species, some mites could start to exploit more phylogenetically distant hosts, possibly compensating for the negative effects of interspecific competition. As a result, positive relationships between mite *dda*_*sp*_ and their host spectra’s phylogenetic diversity was detected in component metacommunities of host species in which *dda*_*region*_ was relatively high (*C. rufocanus*, *M. oeconomus*, and *S. araneus*).

Although ectoparasite species traits were found to affect their dark diversity affinity, the values of *dda*_*sp*_ and, consequently, the probability to belong to the dark diversity of either a region or a host species could differ in the same flea or mite species between the component or compound metacommunities in which these species occurred. For example, a flea, *Ctenopthalmus orientalis*, Wagner, 1898, and a mite, *Myonyssus ingricus*, Bregetova, 1956, occurred in the component metacommunities of four and five host species, respectively, but the values of their *dda*_*sp*_ > 0.5 were detected in only two and four of these metacommunities, respectively (Supplementary Tables S4–S7, S12–S13). Similarly, the values of *dda*_*sp*_ > 0.5 were found in a flea, *Rhadinopsylla integella*, Jordan et Rothschild, 1921, and a mite, *Laelaps micromydis*, Zakhvatkin 1948, in two of five and three of four compound metacommunities where they occurred, respectively (Supplementary Tables S15–S16, S18–S19). This suggests that the probability of an ectoparasite species to belong to the dark diversity of a region (in component metacommunities) or a host species (in compound metacommunities) depends not only on its traits but also on regional conditions and/or host species traits. In other words, the dark diversity affinity of an ectoparasite species is realized as an interplay between ectoparasite traits, regional characteristics, and host species traits.

The effects of regional environment and the number of available host species on the probability of a region to lack suitable ectoparasites could be expected. This is because of the sensitivity of both fleas and mites to environmental factors such as air temperature and relative humidity (which strongly depends on precipitation) (see reviews in Radovsky 1985; Marshall 1981; Krasnov 2008). These factors affect the feeding rate (Kozlova 1982; Gong et al. 2004), reproduction (Kozlova 1983; Krasnov et al. 2001a), and survival (Kozlova 1983; Krasnov et al. 2001b) in both taxa. NDVI measures the amount of green vegetation, which likely affects the microclimate in and around the burrows where the majority of flea and mite species reside and reproduce (Radovsky 1985; Krasnov 2008). Moreover, different flea and mite species are characterized by different preferred ranges of temperature and humidity (e.g., Krasnov et al. 2001a). This may explain why the signs of coefficients of environmental variables suggested that the higher probability of a region to lack suitable ectoparasite species could be associated with either higher or lower values of air temperature, precipitation, or NDVI. These signs could depend on the flea and mite species composition of a component metacommunity and the preferred environmental conditions of these species, a mediating role of the environment in ectoparasite-host relationship (e.g., Krasnov et al. 1998), and the degree of environmental heterogeneity within a region.

In the majority of compound communities, we failed to find traits that affected the hosts’ dark diversity affinity (i.e., *dda*_*host*_). This does not, however, mean that *dda*_*host*_ is not associated with host traits, merely that the traits that may be important in determining host dark diversity were not considered in our study. For example, we did not consider host density because (a) the density of small mammals is highly variable, both spatially and temporally, and (b) these data were unavailable. Another host trait that may potentially affect the dark diversity of their parasite assemblages is immunocompetence (the ability to cope with parasitism), which often varies interspecifically (e.g., Klein and Nelson 1998; Goüy de Bellocq et al. 2006), but these data are, again, unavailable.

A comparison of this study’s results with the results of our earlier studies on ectoparasite dark diversity (Krasnov et al. 2022a, c) suggests that the species-site unified model produces more informative results than studies that separately consider the effects of either sites or hosts on ectoparasite dark diversity. Krasnov et al. (2022a) calculated parasite dark diversity size for a region or a host species as the sum of the probabilities of all parasite species absent from the region or the host, respectively, to belong to dark diversity. Krasnov et al. (2022c) applied the dark diversity concept to a parasite’s host spectrum, defined it as dark host specificity, and, subsequently, calculated the dark diversity of a host species as the probability of each host species that is absent from an ectoparasite species’ regional host spectrum to belong or not to belong to this ectoparasite’s dark host specificity. Both these studies lacked an important component, namely, they did not consider the ectoparasite species characteristics that determine their dark diversity affinities, whereas the species-site unified model allowed identifying these characteristics. Nevertheless, taken together, the results of the earlier two studies and this study allows us to conclude that the dark diversity of ectoparasite component and compound metacommunities is a combination of the effects of ectoparasite species traits, the regional abiotic and biotic environment, and host species traits. This combination seems to be the main reason for the dark diversity affinity of an ectoparasite species or a region (for component metacommunities) or a host species (for compound metacommunities) to vary substantially between different metacommunities.

## Supplementary Information

Below is the link to the electronic supplementary material.

Supplementary file1 (DOCX 974 KB)

## Data Availability

The data on fleas can be found in Mendeley data Mendeley Data, V2, doi: 10.17632/97 × 46ggb2k.2, data on mites can be obtained from corresponding author upon reasonable request.
