# Supplementary material for: Parasite traits, host traits, and environment as determinants of dark diversity affinity in flea and gamasid mite assemblages from the Palearctic
Source: Parasitol Res. 2024 Nov 26;123(11):396. doi: 10.1007/s00436-024-08408-6 (PMC11599333; doi:10.1007/s00436-024-08408-6)
Supplement: Supplementary file 1 — Supplementary file1 (DOCX 974 KB) [file 436_2024_8408_MOESM1_ESM.docx]

**Supplementary Figures**

**Supplementary Figure S1.** Palearctic regions where surveys of fleas and their small mammalian hosts were carried out. The number near the marker corresponds to the number in Supplementary Table S1.


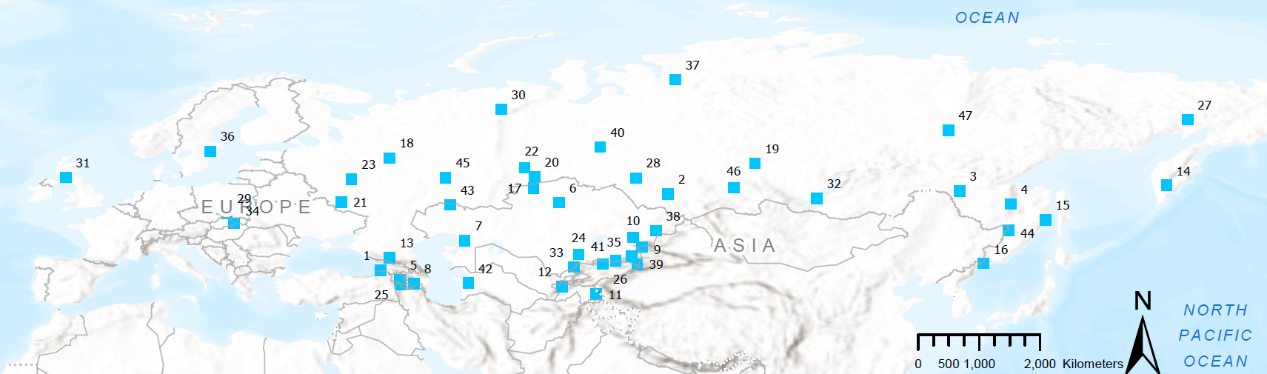


**Supplementary Figure S2.** Palearctic regions where surveys of gamasid mites and their small mammalian hosts were carried out. The number near the marker corresponds to the number in Supplementary Table S2.


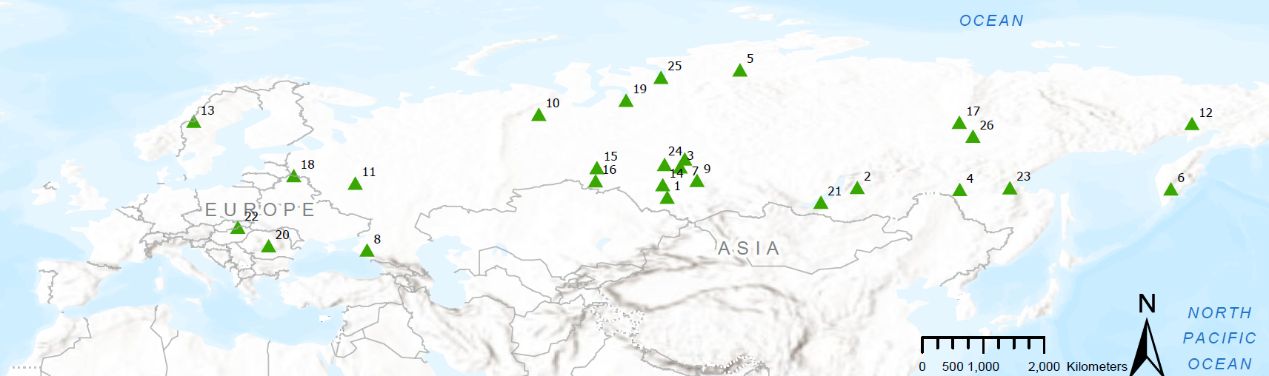


**Supplementary Tables**

**Supplementary Table S1.** Regions for which data on fleas were used in the analyses. Region: name of a region, Description: some description of regional components (when needed). Asterisks denote regions selected for the analyses of compound communities (see text for explanation).

| Region | Description | Number on map |
| --- | --- | --- |
| Adzharia | Lesser Caucasus | 1 |
| Altai Mountains* |  | 2 |
| Amur | Central Amur River Valley | 3 |
| Amur-Bureya | Left tributary of the Amur River | 4 |
| Armenia* |  | 5 |
| Astana | North-central Kazakhstan | 6 |
| Atyrau | Northeastern Caspian shore | 7 |
| Azerbaijan | Southwestern Azerbaijan | 8 |
| Dzungarian Alatau* | Northern Tian Shan | 9 |
| East Balkhash | Eastern shore of the Balkhash Lake | 10 |
| East Pamir | Eastern Pamir Mountains | 11 |
| Gissar | Western Tajikistan | 12 |
| Kabarda | North Caucasus | 13 |
| Kamchatka | Kamchatka Peninsula | 14 |
| Khabarovsk | Confluence of the Amur and Ussury Rivers | 15 |
| Khasan | Southern Russian Far East | 16 |
| Kostanay | Northern Kazakhstan | 17 |
| Kostroma | Western European Russia | 18 |
| Krasnojarsk | Central Siberia | 19 |
| Kurgan* | Southern Ural Mountains | 20 |
| Kursk | Southern slopes of the middle-Russian plateau | 21 |
| Middle Ural | Central Ural Mountains | 22 |
| Moscow | Central European Russia | 23 |
| Moiunkum | Desert in southern Kazakhstan | 24 |
| Nakhichevan | Southeastern Transcaucasus plateau | 25 |
| North Kyrgyzstan |  | 26 |
| Northern Russian Far East |  | 27 |
| Novosibirsk | Southwestern Siberia | 28 |
| Poland* |  | 29 |
| Polar Ural | Northernmost Ural Mountains | 30 |
| Scotland |  | 31 |
| Selenga | Southern Siberia | 32 |
| Shymkent | Southernmost Kazakhstan | 33 |
| Slovakia |  | 34 |
| Sweden |  | 35 |
| Syugaty | Eastern Kazakhstan | 36 |
| Taimyr | Taimyr Peninsula | 37 |
| Tarbagatai | Eastern Kazakhstan | 38 |
| Terskey-Alatau | Eastern Kyrgyzstan | 39 |
| Tomsk-Tyumen* | Southeastern Siberia | 40 |
| Trans-Ili_Alatau | Northernmost Tian Shan | 41 |
| Turkmenistan* |  | 42 |
| Ural Valley | Ural River Valley | 43 |
| Ussury | Ussury River Valley | 44 |
| Tatarstan (Volga-Kama)* | Eastern European Russia | 45 |
| Western Sayan | Southern Siberia | 46 |
| Yakutia |  | 47 |

**Supplementary Table S2.** Regions for which data on gamasid mites were used in the analyses. Region: name of a region, Description: some description of regional components (when needed). Asterisks denote regions selected for the analyses of compound communities (see text for explanation).

| Region | Description | Number on map |
| --- | --- | --- |
| Altai Mountains |  | 1 |
| Buryatia | Southeastern Siberia | 2 |
| Chulym River* | Khakassia (southern Siberia) | 3 |
| East BAM | Eastern Siberia | 4 |
| East Taimyr | Eastern Taimyr Peninsula | 5 |
| Kamchatka | Kamchatka Peninsula | 6 |
| Ket River | Right tributary of the Ob River | 7 |
| Krasnodar* | Southwestern Russia | 8 |
| Kuznetsk Alatau | Southern Siberia | 9 |
| Ob River Floodplain | Lower Ob River Floodplain | 10 |
| Moscow | Central European Russia | 11 |
| Northern Russian Far East* |  | 12 |
| Norway |  | 13 |
| Novosibirsk* | Southwestern Siberia | 14 |
| Omsk Forest Zone | Southwestern Siberia | 15 |
| Omsk Forest-Steppe Zone* | Southwestern Siberia | 16 |
| Predverkhoyanie | Eastern Yakutia | 17 |
| Pskov | Northern European Russia | 18 |
| Pur River | Northwestern Siberia | 19 |
| Romania |  | 20 |
| Selenga | Southern Siberia | 21 |
| Slovakia |  | 22 |
| Southern Russian Far East* |  | 23 |
| Tomsk | Southwest Siberia | 24 |
| West Taimyr | Western Taimyr Peninsula | 25 |
| Yakutia | Central Yakutia | 26 |

**Supplementary Table S3.** Datasets used in the analyses of component metacommunities (i.e., within a host species between regions) and compound metacommunities (i.e., within a region between host species. N_rh_: number of regions (for component metacommunities) or host species (for compound metacommunities). N_sp_: number of flea or mite species. PR: presence rate (number of presences divided by matrix size).

| Parasite | Metacommunity | Host species/Region | N_rh_ | N_sp_ | PR |
| --- | --- | --- | --- | --- | --- |
| Fleas | Component | *Apodemus agrarius* | 15 | 54 | 0.19 |
|  |  | *Apodemus uralensis* | 18 | 61 | 0.16 |
|  |  | *Cricetulus migratorius* | 15 | 76 | 0.13 |
|  |  | *Microtus arvalis* | 18 | 59 | 0.19 |
|  |  | *Microtus oeconomus* | 15 | 28 | 0.23 |
|  |  | *Myodes rutilus* | 19 | 55 | 0.21 |
|  |  | *Sorex araneus* | 20 | 42 | 0.21 |
|  | Compound | Altai Mountains | 15 | 8 | 0.73 |
|  |  | Armenia | 16 | 38 | 0.20 |
|  |  | Dzungarian Alatau | 14 | 22 | 0.31 |
|  |  | Kurgan | 14 | 16 | 0.44 |
|  |  | Poland | 15 | 25 | 0.49 |
|  |  | Tomsk-Tyumen | 20 | 25 | 0.35 |
|  |  | Turkmenistan | 15 | 33 | 0.35 |
|  |  | Tatarstan | 17 | 30 | 0.40 |
| Mites | Component | *Craseomys rufocanus* | 13 | 26 | 0.31 |
|  |  | *Microtus oeconomus* | 18 | 26 | 0.35 |
|  |  | *Myodes glareolus* | 12 | 28 | 0.29 |
|  |  | *Myodes rutilus* | 19 | 31 | 0.28 |
|  |  | *Sorex araneus* | 18 | 37 | 0.24 |
|  | Compound | Chulym River | 12 | 14 | 0.35 |
|  |  | Krasnodar | 14 | 24 | 0.23 |
|  |  | Northern Russian Far East | 11 | 18 | 0.27 |
|  |  | Novosibirsk | 21 | 17 | 0.51 |
|  |  | Omsk Forest-Steppe Zone | 18 | 23 | 0.47 |
|  |  | Southern Russian Far East | 14 | 29 | 0.33 |

**Supplementary Table S4.** Percentiles (2.5% and 97.5%) of posterior distributions of *dda_sp_* for flea species estimated by the species-region unified model for the component metacommunity of *Apodemus agrarius*. *dda_sp_* values significantly (based on a 95% credible interval) larger than 0.5 indicate an absence tendency (bold blue font), whereas *dda_sp_* values significantly (based on a 95% credible interval) smaller than 0.5 indicate a presence tendency (bold red font).

| Flea species | Percentile | |
| --- | --- | --- |
|  | 2.5% | 97.5% |
| *Amalaraeus penicilliger* | **0.00** | **0.04** |
| *Amphipsylla rossica* | **0.00** | **0.09** |
| *Amphipsylla sibirica* | 0.01 | 0.71 |
| *Amphipsylla vinogradovi* | 0.17 | 0.97 |
| *Ceratophyllus anisus* | **0.52** | **1.00** |
| *Ceratophyllus indages* | 0.01 | 0.78 |
| *Corrodopsylla birulai* | **0.01** | **0.49** |
| *Ctenophthalmus agyrtes* | **0.00** | **0.39** |
| *Ctenophthalmus assimilis* | **0.00** | **0.10** |
| *Ctenophthalmus bisoctodentatus* | **0.70** | **1.00** |
| *Ctenophthalmus congener* | 0.26 | 0.98 |
| *Ctenophthalmus congeneroides* | 0.19 | 0.93 |
| *Ctenophthalmus orientalis* | **0.53** | **0.99** |
| *Ctenophthalmus proximus* | 0.01 | 0.52 |
| *Ctenophthalmus secundus* | 0.12 | 0.86 |
| *Ctenophthalmus solutus* | **0.62** | **0.99** |
| *Ctenophthalmus uncinatus* | **0.02** | **0.48** |
| *Ctenophthalmus wagneri* | 0.07 | 0.73 |
| *Doratopsylla dasycnema* | **0.00** | **0.40** |
| *Frontopsylla elata* | **0.00** | **0.27** |
| *Frontopsylla luculenta* | 0.15 | 0.98 |
| *Frontopsylla protera* | 0.32 | 0.99 |
| *Hystrichopsylla microti* | 0.14 | 0.99 |
| *Hystrichopsylla orientalis* | 0.00 | 0.80 |
| *Hystrichopsylla talpae* | **0.00** | **0.02** |
| *Leptopsylla segnis* | 0.02 | 0.88 |
| *Leptopsylla taschenbergi* | 0.09 | 0.88 |
| *Megabothris advenarius* | 0.40 | 0.99 |
| *Megabothris calcarifer* | 0.03 | 0.79 |
| *Megabothris rectangulatus* | **0.00** | **0.13** |
| *Megabothris turbidus* | **0.00** | **0.06** |
| *Megabothris walkeri* | 0.04 | 0.82 |
| *Neopsylla acanthina* | **0.54** | **0.98** |
| *Neopsylla bidentatiformis* | **0.59** | **0.98** |
| *Neopsylla mana* | **0.00** | **0.45** |
| *Neopsylla pleskei* | **0.00** | **0.07** |
| *Nosopsyllus consimilis* | 0.02 | 0.95 |
| *Nosopsyllus fasciatus* | 0.01 | 0.78 |
| *Nosopsyllus fidus* | 0.09 | 0.93 |
| *Nosopsyllus mokrzeckyi* | 0.03 | 0.79 |
| *Palaeopsylla soricis* | **0.00** | **0.26** |
| *Paradoxopsyllus scorodumovi* | 0.06 | 0.99 |
| *Pectinoctenus nemorosa* | 0.46 | 0.99 |
| *Pectinoctenus pectiniceps* | **0.69** | **1.00** |
| *Peromyscopsylla bidentata* | 0.03 | 0.70 |
| *Peromyscopsylla fallax* | **0.73** | **1.00** |
| *Peromyscopsylla ostsibirica* | **0.66** | **1.00** |
| *Peromyscopsylla silvatica* | 0.04 | 0.74 |
| *Rhadinopsylla cedestis* | 0.38 | 0.99 |
| *Rhadinopsylla insolita* | **0.80** | **1.00** |
| *Rhadinopsylla integella* | 0.08 | 0.89 |
| *Rhadinopsylla li* | 0.12 | 0.98 |
| *Rhadinopsylla rothschildi* | **0.89** | **1.00** |
| *Stenoponia sidimi* | 0.41 | 0.99 |

**Supplementary Table S5.** Percentiles (2.5% and 97.5%) of posterior distributions of *dda_sp_* for flea species estimated by the species-region unified model for the component metacommunity of *Apodemus uralensis*. *dda_sp_* values significantly (based on a 95% credible interval) larger than 0.5 indicate an absence tendency (bold blue font), whereas *dda_sp_* values significantly (based on a 95% credible interval) smaller than 0.5 indicate a presence tendency (bold red font).

| Flea species | Percentile | |
| --- | --- | --- |
|  | 2.5% | 97.5% |
| *Amalaraeus penicilliger* | **0.00** | **0.11** |
| *Amphipsylla anceps* | **0.77** | **0.99** |
| *Amphipsylla asiatica* | 0.44 | 0.98 |
| *Amphipsylla dumalis* | **0.51** | **0.99** |
| *Amphipsylla kuznetzovi* | **0.54** | **0.98** |
| *Amphipsylla montana* | **0.84** | **1.00** |
| *Amphipsylla phaiomydis* | 0.42 | 0.96 |
| *Amphipsylla primaris* | 0.12 | 0.95 |
| *Amphipsylla rossica* | **0.00** | **0.10** |
| *Amphipsylla sibirica* | 0.11 | 0.82 |
| *Amphipsylla vinogradovi* | **0.62** | **0.98** |
| *Callopsylla caspia* | 0.29 | 0.93 |
| *Ceratophyllus sciurorum* | 0.14 | 0.85 |
| *Ceratophyllus sinicus* | **0.58** | **0.99** |
| *Citellophilus lebedewi* | 0.02 | 0.67 |
| *Citellophilus tesquorum* | **0.01** | **0.45** |
| *Corrodopsylla birulai* | **0.00** | **0.25** |
| *Ctenophthalmus agyrtes* | **0.01** | **0.22** |
| *Ctenophthalmus arvalis* | 0.08 | 0.75 |
| *Ctenophthalmus assimilis* | **0.00** | **0.07** |
| *Ctenophthalmus breviatus* | 0.06 | 0.68 |
| *Ctenophthalmus dilatatus* | **0.54** | **0.99** |
| *Ctenophthalmus golovi* | **0.52** | **0.97** |
| *Ctenophthalmus orientalis* | 0.43 | 0.97 |
| *Ctenophthalmus solutus* | **0.58** | **0.98** |
| *Ctenophthalmus uncinatus* | **0.01** | **0.31** |
| *Ctenophthalmus wagneri* | 0.06 | 0.59 |
| *Doratopsylla dasycnema* | **0.01** | **0.44** |
| *Frontopsylla ambigua* | **0.68** | **0.99** |
| *Frontopsylla elata* | **0.01** | **0.38** |
| *Frontopsylla ornata* | **0.59** | **0.99** |
| *Frontopsylla protera* | 0.34 | 0.98 |
| *Frontopsylla semura* | **0.63** | **0.98** |
| *Hystrichopsylla orientalis* | 0.04 | 0.98 |
| *Hystrichopsylla talpae* | **0.00** | **0.22** |
| *Leptopsylla nana* | 0.41 | 0.97 |
| *Leptopsylla segnis* | 0.03 | 0.80 |
| *Leptopsylla sicistae* | **0.52** | **0.99** |
| *Leptopsylla taschenbergi* | **0.02** | **0.42** |
| *Megabothris rectangulatus* | **0.00** | **0.16** |
| *Megabothris turbidus* | **0.00** | **0.07** |
| *Megabothris walkeri* | 0.09 | 0.79 |
| *Neopsylla mana* | 0.03 | 0.60 |
| *Neopsylla pleskei* | **0.00** | **0.09** |
| *Neopsylla teratura* | **0.66** | **0.99** |
| *Nosopsyllus consimilis* | 0.40 | 0.98 |
| *Nosopsyllus fidus* | 0.35 | 0.95 |
| *Nosopsyllus mokrzeckyi* | 0.21 | 0.91 |
| *Ophthalmopsylla praefecta* | **0.64** | **1.00** |
| *Oropsylla silantiewi* | 0.15 | 0.97 |
| *Palaeopsylla soricis* | **0.00** | **0.03** |
| *Paradoxopsyllus scorodumovi* | 0.28 | 0.99 |
| *Paraneopsylla ioffi* | 0.25 | 0.91 |
| *Pectinoctenus lauta* | 0.32 | 0.95 |
| *Pectinoctenus nemorosa* | 0.22 | 0.88 |
| *Pectinoctenus pectiniceps* | 0.30 | 0.94 |
| *Peromyscopsylla bidentata* | **0.02** | **0.37** |
| *Peromyscopsylla silvatica* | **0.01** | **0.35** |
| *Rhadinopsylla altifrons* | 0.34 | 0.99 |
| *Rhadinopsylla integella* | 0.07 | 0.77 |
| *Rhadinopsylla li* | 0.42 | 0.98 |

**Supplementary Table S6.** Percentiles (2.5% and 97.5%) of posterior distributions of *dda_sp_* for flea species estimated by the species-region unified model for the component metacommunity of *Cricetulus migratorius*. *dda_sp_* values significantly (based on a 95% credible interval) larger than 0.5 indicate an absence tendency (bold blue font), whereas *dda_sp_* values significantly (based on a 95% credible interval) smaller than 0.5 indicate a presence tendency (bold red font).

| Flea species | Percentile | |
| --- | --- | --- |
|  | 2.5% | 97.5% |
| *Amalaraeus penicilliger* | 0.01 | 0.72 |
| *Amphipsylla anceps* | 0.01 | 0.80 |
| *Amphipsylla asiatica* | 0.00 | 0.78 |
| *Amphipsylla dumalis* | **0.00** | **0.25** |
| *Amphipsylla georgica* | 0.01 | 0.86 |
| *Amphipsylla kuznetzovi* | 0.00 | 0.80 |
| *Amphipsylla montana* | 0.01 | 0.88 |
| *Amphipsylla phaiomydis* | 0.00 | 0.63 |
| *Amphipsylla primaris* | **0.00** | **0.22** |
| *Amphipsylla rossica* | **0.00** | **0.10** |
| *Amphipsylla schelkovnikovi* | **0.00** | **0.17** |
| *Callopsylla caspia* | 0.10 | 0.91 |
| *Citellophilus lebedewi* | 0.00 | 0.27 |
| *Citellophilus transcaucasicus* | 0.08 | 0.94 |
| *Citellophilus trispinus* | 0.07 | 0.80 |
| *Coptopsylla lamellifer* | 0.01 | 0.97 |
| *Ctenophthalmus arvalis* | 0.01 | 0.62 |
| *Ctenophthalmus assimilis* | 0.00 | 0.54 |
| *Ctenophthalmus dilatatus* | 0.00 | 0.84 |
| *Ctenophthalmus dolichus* | 0.03 | 0.82 |
| *Ctenophthalmus hypanis* | 0.19 | 0.96 |
| *Ctenophthalmus inornatus* | 0.04 | 0.84 |
| *Ctenophthalmus orientalis* | 0.13 | 0.95 |
| *Ctenophthalmus proximus* | 0.02 | 0.73 |
| *Ctenophthalmus secundus* | 0.07 | 0.84 |
| *Ctenophthalmus wagneri* | 0.04 | 0.79 |
| *Echidnophaga oschanini* | 0.07 | 1.00 |
| *Frontopsylla elata* | 0.03 | 0.85 |
| *Frontopsylla elatoides* | 0.31 | 0.97 |
| *Frontopsylla ornata* | **0.56** | **1.00** |
| *Frontopsylla protera* | 0.40 | 0.99 |
| *Hystrichopsylla talpae* | 0.00 | 0.52 |
| *Leptopsylla nana* | 0.11 | 0.93 |
| *Leptopsylla segnis* | 0.03 | 0.95 |
| *Leptopsylla taschenbergi* | 0.14 | 0.96 |
| *Megabothris rectangulatus* | **0.00** | **0.36** |
| *Megabothris turbidus* | **0.00** | **0.31** |
| *Mesopsylla hebes* | 0.01 | 0.83 |
| *Neopsylla democratica* | 0.23 | 0.98 |
| *Neopsylla mana* | **0.00** | **0.43** |
| *Neopsylla meridiana* | 0.01 | 0.70 |
| *Neopsylla pleskei* | **0.00** | **0.23** |
| *Neopsylla setosa* | **0.00** | **0.32** |
| *Neopsylla teratura* | 0.02 | 0.81 |
| *Nosopsyllus aralis* | 0.12 | 0.91 |
| *Nosopsyllus consimilis* | 0.00 | 0.54 |
| *Nosopsyllus fasciatus* | 0.02 | 0.95 |
| *Nosopsyllus fidus* | 0.09 | 0.87 |
| *Nosopsyllus iranus* | 0.07 | 0.84 |
| *Nosopsyllus laeviceps* | 0.01 | 0.51 |
| *Nosopsyllus mokrzeckyi* | 0.15 | 0.96 |
| *Nosopsyllus philippovi* | 0.32 | 0.98 |
| *Nosopsyllus turkmenicus* | 0.32 | 0.99 |
| *Ophthalmopsylla praefecta* | 0.05 | 0.98 |
| *Ophthalmopsylla volgensis* | 0.01 | 0.69 |
| *Oropsylla ilovaiskii* | 0.01 | 0.85 |
| *Paradoxopsyllus hesperius* | 0.17 | 0.95 |
| *Paradoxopsyllus naryni* | 0.48 | 0.99 |
| *Paraneopsylla ioffi* | 0.19 | 0.98 |
| *Paraneopsylla tiflovi* | 0.22 | 0.98 |
| *Pectinoctenus lauta* | 0.16 | 0.97 |
| *Pectinoctenus nemorosa* | 0.07 | 0.92 |
| *Pectinoctenus pamirensis* | 0.61 | 1.00 |
| *Rhadinopsylla altifrons* | 0.02 | 0.88 |
| *Rhadinopsylla bivirgis* | 0.11 | 0.96 |
| *Rhadinopsylla cedestis* | 0.04 | 0.93 |
| *Rhadinopsylla dahurica* | 0.00 | 0.75 |
| *Rhadinopsylla li* | 0.00 | 0.52 |
| *Rhadinopsylla ucrainica* | 0.05 | 0.94 |
| *Rostropsylla daca* | 0.14 | 0.96 |
| *Wagnerina schelkovnikovi* | 0.01 | 0.88 |
| *Xenopsylla cheopis* | 0.00 | 0.69 |
| *Xenopsylla conformis* | 0.00 | 0.34 |
| *Xenopsylla gerbilli* | 0.05 | 0.97 |
| *Xenopsylla nuttalli* | 0.07 | 0.98 |
| *Xenopsylla persica* | 0.15 | 0.99 |

**Supplementary Table S7.** Percentiles (2.5% and 97.5%) of posterior distributions of *dda_sp_* for flea species estimated by the species-region unified model for the component metacommunity of *Microtus arvalis*. *dda_sp_* values significantly (based on a 95% credible interval) larger than 0.5 indicate an absence tendency (bold blue font), whereas *dda_sp_* values significantly (based on a 95% credible interval) smaller than 0.5 indicate a presence tendency (bold red font).

| Flea species | Percentile | |
| --- | --- | --- |
|  | 2.5% | 97.5% |
| *Amalaraeus arvicolae* | 0.18 | 0.98 |
| *Amalaraeus penicilliger* | **0.00** | **0.04** |
| *Amphipsylla kuznetzovi* | **0.50** | **0.98** |
| *Amphipsylla rossica* | **0.00** | **0.02** |
| *Amphipsylla schelkovnikovi* | 0.03 | 0.88 |
| *Amphipsylla sibirica* | 0.10 | 0.87 |
| *Amphipsylla vinogradovi* | **0.62** | **0.99** |
| *Callopsylla caspia* | **0.55** | **0.98** |
| *Callopsylla saxatilis* | **0.82** | **1.00** |
| *Catallagia dacenkoi* | 0.45 | 0.99 |
| *Ceratophyllus sciurorum* | 0.06 | 0.76 |
| *Citellophilus tesquorum* | **0.00** | **0.07** |
| *Citellophilus transcaucasicus* | **0.57** | **0.99** |
| *Corrodopsylla birulai* | **0.00** | **0.06** |
| *Ctenophthalmus agyrtes* | **0.00** | **0.12** |
| *Ctenophthalmus arvalis* | 0.33 | 0.97 |
| *Ctenophthalmus assimilis* | **0.00** | **0.05** |
| *Ctenophthalmus bisoctodentatus* | **0.66** | **0.99** |
| *Ctenophthalmus bogatschevi* | **0.91** | **1.00** |
| *Ctenophthalmus breviatus* | 0.19 | 0.93 |
| *Ctenophthalmus congener* | **0.84** | **1.00** |
| *Ctenophthalmus golovi* | **0.67** | **0.98** |
| *Ctenophthalmus hypanis* | **0.95** | **1.00** |
| *Ctenophthalmus inornatus* | **0.69** | **0.99** |
| *Ctenophthalmus orientalis* | **0.94** | **1.00** |
| *Ctenophthalmus proximus* | 0.03 | 0.58 |
| *Ctenophthalmus shovi* | **0.93** | **1.00** |
| *Ctenophthalmus solutus* | **0.92** | **1.00** |
| *Ctenophthalmus teres* | **0.81** | **0.99** |
| *Ctenophthalmus uncinatus* | **0.02** | **0.40** |
| *Ctenophthalmus wagneri* | 0.11 | 0.76 |
| *Ctenophthalmus wladimiri* | **0.92** | **1.00** |
| *Doratopsylla dasycnema* | 0.04 | 0.75 |
| *Frontopsylla elata* | **0.00** | **0.34** |
| *Frontopsylla elatoides* | **0.73** | **1.00** |
| *Frontopsylla ornata* | **0.98** | **1.00** |
| *Frontopsylla protera* | **0.84** | **1.00** |
| *Hystrichopsylla orientalis* | **0.00** | **0.24** |
| *Hystrichopsylla talpae* | **0.00** | **0.001** |
| *Leptopsylla segnis* | 0.13 | 0.94 |
| *Leptopsylla taschenbergi* | 0.09 | 0.87 |
| *Megabothris rectangulatus* | **0.00** | **0.04** |
| *Megabothris turbidus* | **0.00** | **0.01** |
| *Megabothris walkeri* | **0.01** | **0.46** |
| *Neopsylla acanthina* | 0.73 | 0.99 |
| *Neopsylla mana* | **0.00** | **0.42** |
| *Neopsylla pleskei* | **0.00** | **0.02** |
| *Neopsylla teratura* | **0.61** | **0.99** |
| *Nosopsyllus consimilis* | 0.13 | 0.95 |
| *Nosopsyllus fasciatus* | 0.35 | 0.98 |
| *Nosopsyllus mokrzeckyi* | **0.71** | **0.99** |
| *Palaeopsylla soricis* | **0.00** | **0.02** |
| *Paradoxopsyllus scorodumovi* | 0.23 | 0.99 |
| *Pectinoctenus nemorosa* | 0.42 | 0.99 |
| *Peromyscopsylla bidentata* | **0.00** | **0.17** |
| *Peromyscopsylla silvatica* | **0.01** | **0.32** |
| *Rhadinopsylla integella* | 0.21 | 0.94 |
| *Rhadinopsylla li* | 0.02 | 0.89 |
| *Stenoponia ivanovi* | 0.03 | 0.97 |

**Supplementary Table S8.** Percentiles (2.5% and 97.5%) of posterior distributions of *dda_sp_* for flea species estimated by the species-region unified model for the component metacommunity of *Microtus oeconomus*. *dda_sp_* values significantly (based on a 95% credible interval) larger than 0.5 indicate an absence tendency (bold blue font), whereas *dda_sp_* values significantly (based on a 95% credible interval) smaller than 0.5 indicate a presence tendency (bold red font).

| Flea species | Percentile | |
| --- | --- | --- |
|  | 2.5% | 97.5% |
| *Amalaraeus penicilliger* | **0.00** | **0.14** |
| *Amphipsylla marikovskii* | 0.05 | 0.98 |
| *Amphipsylla rossica* | 0.00 | 0.56 |
| *Amphipsylla sibirica* | 0.01 | 0.89 |
| *Catallagia dacenkoi* | 0.00 | 0.85 |
| *Catallagia ioffi* | 0.05 | 0.93 |
| *Corrodopsylla birulai* | 0.01 | 0.77 |
| *Ctenophthalmus agyrtes* | 0.45 | 1.00 |
| *Ctenophthalmus assimilis* | 0.01 | 0.88 |
| *Ctenophthalmus breviatus* | **0.67** | **1.00** |
| *Ctenophthalmus uncinatus* | 0.24 | 0.98 |
| *Ctenophthalmus wagneri* | 0.33 | 0.99 |
| *Frontopsylla elata* | 0.00 | 0.58 |
| *Hystrichopsylla talpae* | 0.00 | 0.93 |
| *Megabothris advenarius* | 0.04 | 0.95 |
| *Megabothris calcarifer* | 0.01 | 0.67 |
| *Megabothris rectangulatus* | **0.00** | **0.14** |
| *Megabothris turbidus* | **0.00** | **0.24** |
| *Megabothris walkeri* | 0.01 | 0.68 |
| *Neopsylla acanthina* | 0.96 | 1.00 |
| *Neopsylla mana* | 0.10 | 0.99 |
| *Neopsylla pleskei* | 0.18 | 0.98 |
| *Palaeopsylla soricis* | 0.00 | 0.48 |
| *Peromyscopsylla bidentata* | 0.02 | 0.89 |
| *Peromyscopsylla ostsibirica* | 0.05 | 0.99 |
| *Peromyscopsylla silvatica* | 0.02 | 0.87 |
| *Rhadinopsylla integella* | **0.89** | **1.00** |
| *Rhadinopsylla li* | **0.68** | **1.00** |

**Supplementary Table S9.** Percentiles (2.5% and 97.5%) of posterior distributions of *dda_sp_* for flea species estimated by the species-region unified model for the component metacommunity of *Myodes rutilus*. *dda_sp_* values significantly (based on a 95% credible interval) larger than 0.5 indicate an absence tendency (bold blue font), whereas *dda_sp_* values significantly (based on a 95% credible interval) smaller than 0.5 indicate a presence tendency (bold red font).

| Flea species | Percentile | |
| --- | --- | --- |
|  | 2.5% | 97.5% |
| *Amalaraeus dissimilis* | **0.80** | **1.00** |
| *Amalaraeus ioffi* | **0.59** | **0.99** |
| *Amalaraeus penicilliger* | **0.00** | **0.07** |
| *Amphipsylla kuznetzovi* | 0.33 | 0.95 |
| *Amphipsylla marikovskii* | **0.59** | **0.98** |
| *Amphipsylla primaris* | 0.42 | 0.99 |
| *Amphipsylla sibirica* | 0.07 | 0.78 |
| *Amphipsylla vinogradovi* | 0.74 | 1.00 |
| *Catallagia dacenkoi* | 0.04 | 0.73 |
| *Catallagia fetisovi* | 0.34 | 0.96 |
| *Catallagia ioffi* | 0.12 | 0.82 |
| *Catallagia striata* | **0.54** | **0.98** |
| *Ceratophyllus anisus* | **0.56** | **1.00** |
| *Ceratophyllus indages* | **0.64** | **0.99** |
| *Ceratophyllus paradoxus* | 0.31 | 0.97 |
| *Citellophilus tesquorum* | 0.10 | 0.90 |
| *Corrodopsylla birulai* | 0.01 | 0.51 |
| *Ctenophthalmus agyrtes* | **0.00** | **0.44** |
| *Ctenophthalmus arvalis* | 0.25 | 0.94 |
| *Ctenophthalmus assimilis* | 0.00 | 0.04 |
| *Ctenophthalmus congeneroides* | 0.09 | 0.84 |
| *Ctenophthalmus pisticus* | 0.21 | 0.93 |
| *Ctenophthalmus uncinatus* | **0.00** | **0.21** |
| *Ctenophthalmus wagneri* | **0.00** | **0.32** |
| *Ctenophyllus armatus* | 0.46 | 0.98 |
| *Doratopsylla dasycnema* | 0.02 | 0.71 |
| *Frontopsylla elata* | **0.00** | **0.19** |
| *Frontopsylla luculenta* | **0.75** | **1.00** |
| *Hystrichopsylla microti* | 0.06 | 0.97 |
| *Hystrichopsylla talpae* | **0.00** | **0.01** |
| *Leptopsylla segnis* | 0.23 | 0.98 |
| *Megabothris advenarius* | 0.10 | 0.82 |
| *Megabothris asio* | 0.30 | 0.96 |
| *Megabothris bispinosa* | 0.24 | 0.90 |
| *Megabothris calcarifer* | 0.05 | 0.60 |
| *Megabothris rectangulatus* | **0.00** | **0.01** |
| *Megabothris turbidus* | **0.00** | **0.00** |
| *Megabothris walkeri* | 0.01 | 0.50 |
| *Neopsylla acanthina* | 0.65 | 0.99 |
| *Neopsylla mana* | 0.16 | 0.97 |
| *Neopsylla pleskei* | **0.00** | **0.05** |
| *Nosopsyllus fasciatus* | **0.85** | **1.00** |
| *Palaeopsylla soricis* | **0.00** | **0.01** |
| *Pectinoctenus pectiniceps* | 0.03 | 0.80 |
| *Peromyscopsylla bidentata* | **0.00** | **0.19** |
| *Peromyscopsylla ostsibirica* | 0.07 | 0.92 |
| *Peromyscopsylla silvatica* | **0.00** | **0.15** |
| *Rhadinopsylla altaica* | 0.37 | 0.97 |
| *Rhadinopsylla dahurica* | **0.92** | **1.00** |
| *Rhadinopsylla integella* | 0.04 | 0.70 |
| *Rhadinopsylla li* | **0.95** | **1.00** |
| *Rhadinopsylla pseudodahurica* | **0.57** | **0.98** |
| *Rhadinopsylla rothschildi* | **0.77** | **1.00** |
| *Stenoponia montana* | 0.04 | 0.86 |

**Supplementary Table S10.** Percentiles (2.5% and 97.5%) of posterior distributions of *dda_sp_* for flea species estimated by the species-region unified model for the component metacommunity of *Sorex araneus*. *dda_sp_* values significantly (based on a 95% credible interval) larger than 0.5 indicate an absence tendency (bold blue font), whereas *dda_sp_* values significantly (based on a 95% credible interval) smaller than 0.5 indicate a presence tendency (bold red font).

| Flea species | Percentile | |
| --- | --- | --- |
|  | 2.5% | 97.5% |
| *Amalaraeus penicilliger* | **0.00** | **0.11** |
| *Amphipsylla rossica* | **0.02** | **0.40** |
| *Amphipsylla sibirica* | **0.74** | **0.99** |
| *Amphipsylla vinogradovi* | **0.94** | **1.00** |
| *Catallagia dacenkoi* | **0.84** | **1.00** |
| *Ceratophyllus indages* | 0.03 | 0.74 |
| *Citellophilus tesquorum* | 0.02 | 0.50 |
| *Corrodopsylla birulai* | **0.00** | **0.17** |
| *Ctenophthalmus agyrtes* | 0.03 | 0.51 |
| *Ctenophthalmus arvalis* | 0.38 | 0.94 |
| *Ctenophthalmus assimilis* | **0.00** | **0.10** |
| *Ctenophthalmus bisoctodentatus* | **0.60** | **0.98** |
| *Ctenophthalmus breviatus* | 0.20 | 0.84 |
| *Ctenophthalmus congener* | 0.71 | 0.99 |
| *Ctenophthalmus proximus* | 0.23 | 0.90 |
| *Ctenophthalmus shovi* | **0.92** | **1.00** |
| *Ctenophthalmus solutus* | **0.91** | **1.00** |
| *Ctenophthalmus uncinatus* | 0.06 | 0.54 |
| *Ctenophthalmus wagneri* | 0.29 | 0.85 |
| *Doratopsylla dasycnema* | 0.04 | 0.60 |
| *Frontopsylla elata* | 0.02 | 0.52 |
| *Frontopsylla protera* | 0.23 | 0.97 |
| *Hystrichopsylla orientalis* | 0.37 | 0.99 |
| *Hystrichopsylla talpae* | **0.00** | **0.19** |
| *Leptopsylla segnis* | 0.16 | 0.95 |
| *Leptopsylla sicistae* | 0.24 | 0.96 |
| *Leptopsylla taschenbergi* | 0.06 | 0.69 |
| *Megabothris calcarifer* | **0.75** | **0.99** |
| *Megabothris rectangulatus* | **0.02** | **0.42** |
| *Megabothris turbidus* | **0.01** | **0.32** |
| *Megabothris walkeri* | 0.24 | 0.90 |
| *Neopsylla mana* | 0.10 | 0.87 |
| *Neopsylla pleskei* | **0.03** | **0.44** |
| *Nosopsyllus consimilis* | 0.35 | 0.98 |
| *Oropsylla alaskensis* | **0.69** | **1.00** |
| *Palaeopsylla kohauti* | 0.22 | 0.96 |
| *Palaeopsylla soricis* | **0.00** | **0.02** |
| *Paradoxopsyllus scorodumovi* | 0.30 | 0.98 |
| *Peromyscopsylla bidentata* | **0.02** | **0.41** |
| *Peromyscopsylla silvatica* | **0.03** | **0.44** |
| *Rhadinopsylla integella* | 0.55 | 0.98 |
| *Rhadinopsylla li* | 0.30 | 0.98 |

**Supplementary Table S11.** Percentiles (2.5% and 97.5%) of posterior distributions of *dda_region_* for regions estimated by the species-region unified models for flea component metacommunities. *dda_region_* values significantly (based on a 95% credible interval) larger than 0.5 indicate an absence tendency (bold blue font), whereas *dda_region_* values significantly (based on a 95% credible interval) smaller than 0.5 indicate a presence tendency (bold red font).

| Host species | Region | Percentile | |
| --- | --- | --- | --- |
|  |  | 2.5% | 97.5% |
| *Apodemus agrarius* | Altai Mountains | 0.13 | 0.88 |
|  | Dzungarian Alatau | 0.12 | 0.98 |
|  | Kabarda | 0.03 | 0.80 |
|  | Khasan | 0.02 | 0.60 |
|  | Kostroma | 0.04 | 0.66 |
|  | Kurgan | 0.30 | 0.98 |
|  | Kursk | 0.06 | 0.92 |
|  | Moscow | 0.18 | 0.98 |
|  | North Kyrgyzstan | 0.11 | 0.95 |
|  | Novosibirsk | 0.38 | 0.96 |
|  | Poland | **0.00** | **0.45** |
|  | Slovakia | **0.00** | **0.31** |
|  | Tomsk-Tyumen | 0.12 | 0.87 |
|  | Ussury | **0.00** | **0.39** |
| *Apodemus uralensis* | Dzungarian Alatau | **0.00** | **0.11** |
|  | Gissar | **0.00** | **0.14** |
|  | Kostroma | 0.17 | 0.86 |
|  | Kurgan | 0.47 | 0.97 |
|  | Kursk | 0.26 | 0.93 |
|  | Kostanay | 0.11 | 0.78 |
|  | Moscow | 0.38 | 0.97 |
|  | North Kyrgyzstan | 0.07 | 0.63 |
|  | Poland | 0.23 | 0.94 |
|  | Shymkent | **0.03** | **0.40** |
|  | Slovakia | 0.45 | 0.97 |
|  | Syugaty | **0.96** | **1.00** |
|  | Tarbagatai | 0.04 | 0.58 |
|  | Terskey-Alatau | **0.00** | **0.16** |
|  | Trans-Ili_Alatau | 0.04 | 0.58 |
|  | Turkmenistan | **0.51** | **0.99** |
|  | Ural Valley | 0.38 | 0.93 |
|  | Tatarstan | 0.05 | 0.72 |
| *Cricetulus migratorius* | Adzharia | **0.51** | **1.00** |
|  | Armenia | **0.00** | **0.23** |
|  | Azerbaijan | 0.23 | 0.96 |
|  | Dzungarian Alatau | 0.08 | 0.83 |
|  | East Balkhash | 0.08 | 0.88 |
|  | East Pamir | **0.00** | **0.09** |
|  | Gissar | 0.03 | 0.88 |
|  | Atyrau | **0.53** | **1.00** |
|  | Kabarda | 0.06 | 0.82 |
|  | Moiunkum | 0.46 | 0.98 |
|  | North Kyrgyzstan | 0.03 | 0.64 |
|  | Shymkent | 0.26 | 0.94 |
|  | Tarbagatai | 0.05 | 0.85 |
|  | Trans-Ili_Alatau | 0.01 | 0.69 |
|  | Turkmenistan | **0.00** | **0.47** |
| *Microtus arvalis* | Adzharia | 0.00 | 0.67 |
|  | Altai Mountains | 0.26 | 0.90 |
|  | Armenia | **0.00** | **0.15** |
|  | Azerbaijan | 0.15 | 0.83 |
|  | Dzungarian Alatau | **0.00** | **0.35** |
|  | Kabarda | 0.14 | 0.92 |
|  | Kostroma | 0.36 | 0.92 |
|  | Kurgan | 0.45 | 0.99 |
|  | Kursk | 0.08 | 0.85 |
|  | Kostanay | 0.18 | 0.85 |
|  | Middle Ural | **0.88** | **1.00** |
|  | Moscow | 0.09 | 0.87 |
|  | Nakhichevan | 0.01 | 0.61 |
|  | Poland | 0.11 | 0.79 |
|  | Slovakia | 0.47 | 0.97 |
|  | Tomsk-Tyumen | 0.12 | 0.88 |
|  | Ural Valley | 0.14 | 0.82 |
|  | Tatarstan | **0.01** | **0.41** |
| *Microtus oeconomus* | Altai Mountains | 0.01 | 0.57 |
|  | Kamchatka | 0.29 | 1.00 |
|  | Kostroma | 0.02 | 0.85 |
|  | Krasnojarsk | 0.02 | 0.94 |
|  | Kurgan | 0.05 | 0.87 |
|  | Kostanay | 0.10 | 0.81 |
|  | Northern Russian Far East | 0.01 | 0.85 |
|  | Novosibirsk | 0.01 | 0.42 |
|  | Polar Ural | 0.19 | 0.99 |
|  | Selenga | 0.23 | 0.99 |
|  | Taimyr | 0.09 | 0.97 |
|  | Tomsk-Tyumen | **0.00** | **0.25** |
|  | Ural Valley | 0.18 | 0.96 |
|  | Tatarstan | 0.00 | 0.54 |
|  | Western Sayan | 0.01 | 0.86 |
| *Myodes rutilus* | Altai Mountains | 0.15 | 0.80 |
|  | Amur | **0.00** | **0.16** |
|  | Amur-Bureya | **0.00** | **0.17** |
|  | Kamchatka | 0.24 | 0.95 |
|  | Khabarovsk | 0.08 | 0.77 |
|  | Kostroma | 0.50 | 0.99 |
|  | Krasnojarsk | 0.03 | 0.56 |
|  | Kurgan | 0.12 | 0.86 |
|  | Middle Ural | 0.10 | 0.84 |
|  | Northern Russian Far East | 0.11 | 0.86 |
|  | Novosibirsk | 0.12 | 0.83 |
|  | Polar Ural | **0.60** | **0.99** |
|  | Selenga | **0.00** | **0.42** |
|  | Taimyr | 0.37 | 0.97 |
|  | Tomsk-Tyumen | 0.24 | 0.95 |
|  | Ussury River Valley | 0.03 | 0.63 |
|  | Tatarstan | 0.28 | 0.94 |
|  | Western Sayan | **0.01** | **0.43** |
|  | Yakutia | **0.00** | **0.48** |
| *Sorex araneus* | Adzharia | 0.01 | 0.82 |
|  | Altai Mountains | **0.05** | **0.41** |
|  | Astana | 0.26 | 0.82 |
|  | Dzungarian Alatau | 0.36 | 0.99 |
|  | Kostroma | 0.21 | 0.75 |
|  | Kurgan | 0.30 | 0.91 |
|  | Kursk | 0.45 | 0.95 |
|  | Middle Ural | **0.71** | **0.98** |
|  | Moscow | 0.15 | 0.86 |
|  | Novosibirsk | **0.01** | **0.25** |
|  | Poland | **0.03** | **0.37** |
|  | Polar Ural | 0.49 | 0.98 |
|  | Scotland | **0.72** | **0.99** |
|  | Slovakia | 0.10 | 0.61 |
|  | Sweden | **0.74** | **0.99** |
|  | Tomsk-Tyumen | **0.01** | **0.17** |
|  | Ural Valley | 0.46 | 0.92 |
|  | Tatarstan | **0.00** | **0.13** |
|  | Western Sayan | 0.21 | 0.89 |
|  | Yakutia | **0.71** | **1.00** |

**Supplementary Table S12.** Percentiles (2.5% and 97.5%) of posterior distributions of *dda_sp_* for mite species estimated by the species-region unified models for component metacommunities of *Craseomys rufocanus*, *Microtus oeconomus*, and *Myodes glareolus*. *dda_sp_* values significantly (based on a 95% credible interval) larger than 0.5 indicate an absence tendency (bold blue font), whereas *dda_sp_* values significantly (based on a 95% credible interval) smaller than 0.5 indicate a presence tendency (bold red font).

| Host species | Mite species | Percentile | |
| --- | --- | --- | --- |
|  |  | 2.5% | 97.5% |
| *Craseomys rufocanus* | *Androlaelaps casalis* | **0.83** | **1.00** |
|  | *Androlaelaps glasgowi* | **0.00** | **0.17** |
|  | *Androlaelaps pavlovskii* | 0.31 | 1.00 |
|  | *Androlaelaps razumovae* | **0.96** | **1.00** |
|  | *Eulaelaps stabularis* | **0.00** | **0.03** |
|  | *Haemogamasus ambulans* | **0.00** | **0.05** |
|  | *Haemogamasus dauricus* | **0.77** | **1.00** |
|  | *Haemogamasus ivanovi* | 0.11 | 0.95 |
|  | *Haemogamasus liponyssoides* | 0.06 | 0.91 |
|  | *Haemogamasus mandshuricus* | 0.00 | 0.56 |
|  | *Haemogamasus nidi* | **0.00** | **0.18** |
|  | *Haemogamasus nidiformes* | 0.01 | 0.58 |
|  | *Haemogamasus serdjukovae* | **0.71** | **1.00** |
|  | *Hirstionyssus apodemi* | 0.03 | 0.98 |
|  | *Hirstionyssus bregetovae* | **1.00** | **1.00** |
|  | *Hirstionyssus eusoricis* | **0.00** | **0.20** |
|  | *Hirstionyssus isabellinus* | **0.00** | **0.08** |
|  | *Hyperlaelaps arvalis* | 0.01 | 0.57 |
|  | *Laelaps clethrionomydis* | **0.00** | **0.04** |
|  | *Laelaps hilaris* | 0.00 | 0.77 |
|  | *Laelaps lemmi* | **0.92** | **1.00** |
|  | *Laelaps multispinosus* | 0.37 | 1.00 |
|  | *Laelaps nuttalli* | **0.54** | **1.00** |
|  | *Laelaps pavlovskyi* | 0.11 | 0.84 |
|  | *Myonyssus dubinini* | **0.82** | **1.00** |
|  | *Myonyssus ingricus* | **0.79** | **1.00** |
| *Microtus oeconomus* | *Androlaelaps casalis* | 0.30 | 0.98 |
|  | *Androlaelaps glasgowi* | **0.00** | **0.03** |
|  | *Eulaelaps cricetuli* | **0.80** | **1.00** |
|  | *Eulaelaps stabularis* | **0.00** | **0.01** |
|  | *Haemogamasus ambulans* | **0.00** | **0.05** |
|  | *Haemogamasus dauricus* | **0.81** | **1.00** |
|  | *Haemogamasus liponyssoides* | **0.80** | **1.00** |
|  | *Haemogamasus mandshuricus* | 0.01 | 0.55 |
|  | *Haemogamasus nidi* | **0.00** | **0.08** |
|  | *Haemogamasus nidiformes* | **0.00** | **0.32** |
|  | *Hirstionyssus apodemi* | **0.54** | **0.98** |
|  | *Hirstionyssus criceti* | **1.00** | **1.00** |
|  | *Hirstionyssus eusoricis* | **0.01** | **0.40** |
|  | *Hirstionyssus isabellinus* | **0.01** | **0.23** |
|  | *Hirstionyssus transiliensis* | 0.07 | 0.89 |
|  | *Hyperlaelaps amphibius* | **0.97** | **1.00** |
|  | *Hyperlaelaps arvalis* | **0.00** | **0.21** |
|  | *Hyperlaelaps microti* | **0.00** | **0.08** |
|  | *Laelaps alaskensis* | **0.90** | **1.00** |
|  | *Laelaps clethrionomydis* | **0.00** | **0.05** |
|  | *Laelaps hilaris* | **0.00** | **0.24** |
|  | *Laelaps multispinosus* | **0.63** | **0.99** |
|  | *Laelaps muris* | 0.22 | 0.97 |
|  | *Laelaps nuttalli* | **0.54** | **0.99** |
|  | *Laelaps pavlovskyi* | 0.06 | 0.68 |
|  | *Myonyssus ingricus* | **0.97** | **1.00** |
| *Myodes glareolus* | *Androlaelaps casalis* | **0.91** | **1.00** |
|  | *Androlaelaps dogieli* | 0.00 | 0.96 |
|  | *Androlaelaps glasgowi* | **0.00** | **0.08** |
|  | *Eulaelaps stabularis* | **0.00** | **0.21** |
|  | *Haemogamasus ambulans* | **0.00** | **0.13** |
|  | *Haemogamasus hirsutosimilis* | **0.74** | **1.00** |
|  | *Haemogamasus hirsutus* | **0.83** | **1.00** |
|  | *Haemogamasus horridus* | 0.03 | 0.99 |
|  | *Haemogamasus liponyssoides* | 0.12 | 0.98 |
|  | *Haemogamasus mandshuricus* | 0.02 | 1.00 |
|  | *Haemogamasus nidi* | **0.00** | **0.31** |
|  | *Haemogamasus nidiformes* | 0.13 | 0.98 |
|  | *Haemogamasus serdjukovae* | **0.84** | **1.00** |
|  | *Hirstionyssus apodemi* | 0.04 | 0.96 |
|  | *Hirstionyssus carnifex* | **0.93** | **1.00** |
|  | *Hirstionyssus eusoricis* | 0.00 | 0.62 |
|  | *Hirstionyssus isabellinus* | **0.00** | **0.46** |
|  | *Hirstionyssus latiscutatus* | **0.94** | **1.00** |
|  | *Hyperlaelaps arvalis* | 0.07 | 0.95 |
|  | *Hyperlaelaps microti* | 0.03 | 0.98 |
|  | *Laelaps agilis* | 0.00 | 0.69 |
|  | *Laelaps clethrionomydis* | **0.00** | **0.04** |
|  | *Laelaps hilaris* | 0.00 | 0.52 |
|  | *Laelaps muris* | 0.00 | 0.69 |
|  | *Laelaps pavlovskyi* | 0.02 | 0.70 |
|  | *Myonyssus gigas* | 0.45 | 1.00 |
|  | *Myonyssus ingricus* | **0.78** | **1.00** |
|  | *Myonyssus rossicus* | **0.57** | **1.00** |

**Supplementary Table S13.** Percentiles (2.5% and 97.5%) of posterior distributions of *dda_sp_* for mite species estimated by the species-region unified models for the mite component metacommunities of *Myodes rutilus* and *Sorex araneus*. *dda_sp_* values significantly (based on a 95% credible interval) larger than 0.5 indicate an absence tendency (bold blue font), whereas *dda_sp_* values significantly (based on a 95% credible interval) smaller than 0.5 indicate a presence tendency (bold red font).

| Host species | Mite species | Percentile | |
| --- | --- | --- | --- |
|  |  | 2.5% | 97.5% |
| *Myodes rutilus* | *Androlaelaps casalis* | **0.72** | **0.99** |
|  | *Androlaelaps glasgowi* | **0.00** | **0.11** |
|  | *Androlaelaps pavlovskii* | 0.12 | 0.93 |
|  | *Eulaelaps kolpakovae* | 0.33 | 0.95 |
|  | *Eulaelaps stabularis* | **0.00** | **0.01** |
|  | *Haemogamasus ambulans* | **0.00** | **0.05** |
|  | *Haemogamasus dauricus* | **0.87** | **1.00** |
|  | *Haemogamasus ivanovi* | 0.18 | 0.90 |
|  | *Haemogamasus liponyssoides* | 0.16 | 0.97 |
|  | *Haemogamasus mandshuricus* | **0.00** | **0.39** |
|  | *Haemogamasus nidi* | **0.01** | **0.15** |
|  | *Haemogamasus nidiformes* | 0.05 | 0.61 |
|  | *Hirstionyssus apodemi* | **0.64** | **0.98** |
|  | *Hirstionyssus criceti* | **0.88** | **1.00** |
|  | *Hirstionyssus eusoricis* | **0.01** | **0.21** |
|  | *Hirstionyssus gudauricus* | **0.67** | **0.98** |
|  | *Hirstionyssus isabellinus* | **0.00** | **0.12** |
|  | *Hirstionyssus latiscutatus* | **1.00** | **1.00** |
|  | *Hirstionyssus pavlovskyi* | **0.99** | **1.00** |
|  | *Hirstionyssus transiliensis* | 0.31 | 0.98 |
|  | *Hyperlaelaps amphibius* | 0.93 | 1.00 |
|  | *Hyperlaelaps arvalis* | 0.08 | 0.81 |
|  | *Hyperlaelaps microti* | **0.00** | **0.28** |
|  | *Laelaps alaskensis* | **0.54** | **0.99** |
|  | *Laelaps clethrionomydis* | **0.00** | **0.04** |
|  | *Laelaps hilaris* | 0.03 | 0.69 |
|  | *Laelaps multispinosus* | 0.01 | 0.61 |
|  | *Laelaps muris* | 0.07 | 0.90 |
|  | *Laelaps nuttalli* | 0.01 | 0.62 |
|  | *Laelaps pavlovskyi* | **0.04** | **0.50** |
|  | *Myonyssus ingricus* | **0.84** | **1.00** |
| *Sorex araneus* | *Androlaelaps casalis* | **0.57** | **0.99** |
|  | *Androlaelaps dogieli* | 0.07 | 0.95 |
|  | *Androlaelaps glasgowi* | **0.00** | **0.04** |
|  | *Eulaelaps cricetuli* | **0.51** | **0.98** |
|  | *Eulaelaps stabularis* | **0.00** | **0.02** |
|  | *Haemogamasus ambulans* | **0.00** | **0.10** |
|  | *Haemogamasus citelli* | **1.00** | **1.00** |
|  | *Haemogamasus dauricus* | **0.80** | **1.00** |
|  | *Haemogamasus hirsutosimilis* | **0.51** | **0.99** |
|  | *Haemogamasus hirsutus* | **0.56** | **0.99** |
|  | *Haemogamasus horridus* | 0.01 | 0.91 |
|  | *Haemogamasus kusumotoi* | **0.91** | **1.00** |
|  | *Haemogamasus liponyssoides* | 0.05 | 0.67 |
|  | *Haemogamasus mandshuricus* | 0.04 | 0.76 |
|  | *Haemogamasus nidi* | **0.00** | **0.05** |
|  | *Haemogamasus nidiformes* | **0.01** | **0.29** |
|  | *Haemogamasus serdjukovae* | **0.62** | **0.98** |
|  | *Hirstionyssus apodemi* | **0.01** | **0.45** |
|  | *Hirstionyssus criceti* | 0.37 | 0.98 |
|  | *Hirstionyssus eusoricis* | **0.00** | **0.04** |
|  | *Hirstionyssus isabellinus* | **0.00** | **0.02** |
|  | *Hirstionyssus latiscutatus* | **0.96** | **1.00** |
|  | *Hyperlaelaps amphibius* | **0.92** | **1.00** |
|  | *Hyperlaelaps arvalis* | 0.02 | 0.55 |
|  | *Hyperlaelaps microti* | **0.00** | **0.33** |
|  | *Laelaps agilis* | 0.47 | 0.97 |
|  | *Laelaps clethrionomydis* | **0.00** | **0.14** |
|  | *Laelaps hilaris* | **0.00** | **0.25** |
|  | *Laelaps lemmi* | 0.48 | 0.98 |
|  | *Laelaps micromydis* | **1.00** | **1.00** |
|  | *Laelaps multispinosus* | 0.20 | 0.94 |
|  | *Laelaps muris* | 0.03 | 0.69 |
|  | *Laelaps nuttalli* | 0.28 | 0.96 |
|  | *Laelaps pavlovskyi* | 0.18 | 0.80 |
|  | *Myonyssus dubinini* | **0.55** | **0.98** |
|  | *Myonyssus gigas* | **0.89** | **1.00** |
|  | *Myonyssus ingricus* | 0.21 | 0.93 |

**Supplementary Table S14.** Percentiles (2.5% and 97.5%) of posterior distributions of *dda_region_* estimated by the species-region unified models for mite component metacommunities. *dda_region_* values significantly (based on a 95% credible interval) larger than 0.5 indicate an absence tendency (bold blue font), whereas *dda_region_* values significantly (based on a 95% credible interval) smaller than 0.5 indicate a presence tendency (bold red font).

| Host species | Region | Percentile | |
| --- | --- | --- | --- |
|  |  | 2.5% | 97.5% |
| *Craseomys rufocanus* | Buryatia | **0.00** | **0.29** |
|  | Chulym River | 0.36 | 0.94 |
|  | East BAM | 0.05 | 0.56 |
|  | Kamchatka | **0.68** | **1.00** |
|  | Ket River | **0.75** | **1.00** |
|  | Kuznetsk Alatau | 0.11 | 0.99 |
|  | Northern Russian Far East | 0.00 | 0.53 |
|  | Norway | 0.38 | 1.00 |
|  | Novosibirsk | 0.18 | 0.98 |
|  | Omsk Forest Zone | 0.09 | 0.83 |
|  | Omsk Forest-Steppe Zone | 0.03 | 0.88 |
|  | Predverkhoyanie | 0.39 | 1.00 |
|  | Southern Russian Far East | **0.00** | **0.21** |
| *Microtus oeconomus* | Altai Mountains | 0.38 | 0.94 |
|  | Buryatia | 0.01 | 0.71 |
|  | Chulym River | 0.34 | 0.93 |
|  | East Taimyr | 0.04 | 0.77 |
|  | Kamchatka | **0.74** | **0.99** |
|  | Ket River | **0.64** | **0.98** |
|  | Kuznetsk Alatau | **0.53** | **0.99** |
|  | Ob River Floodplain | 0.20 | 0.93 |
|  | Northern Russian Far East | **0.00** | **0.30** |
|  | Norway | 0.34 | 0.99 |
|  | Novosibirsk | 0.02 | 0.73 |
|  | Omsk Forest Zone | 0.30 | 0.95 |
|  | Omsk Forest-Steppe Zone | 0.06 | 0.83 |
|  | Predverkhoyanie | 0.02 | 0.58 |
|  | Pur River | 0.18 | 0.86 |
|  | Selenga | 0.04 | 0.74 |
|  | Tomsk | 0.23 | 0.85 |
|  | West taimyr | 0.04 | 0.66 |
| *Myodes glareolus* | Chulym River | 0.22 | 0.94 |
|  | Ket River | 0.12 | 0.97 |
|  | Kuznetsk Alatau | 0.01 | 0.97 |
|  | Moscow | 0.01 | 0.63 |
|  | Norway | **0.00** | **0.45** |
|  | Novosibirsk | 0.02 | 0.94 |
|  | Omsk Forest Zone | 0.39 | 0.99 |
|  | Omsk Forest-Steppe Zone | 0.32 | 1.00 |
|  | Pskov | 0.00 | 0.55 |
|  | Romania | 0.02 | 0.85 |
|  | Slovakia | 0.01 | 0.68 |
|  | Tomsk | 0.08 | 0.88 |
| *Myodes rutilus* | Buryatia | 0.02 | 0.66 |
|  | Chulym River | 0.11 | 0.74 |
|  | East BAM | 0.15 | 0.75 |
|  | East Taimyr | 0.06 | 0.79 |
|  | Kamchatka | **0.78** | **1.00** |
|  | Ket River | 0.49 | 0.96 |
|  | Kuznetsk Alatau | **0.66** | **0.99** |
|  | Ob River Floodplain | **0.54** | **0.99** |
|  | Northern Russian Far East | **0.00** | **0.22** |
|  | Norway | 0.17 | 0.97 |
|  | Novosibirsk | 0.01 | 0.66 |
|  | Omsk Forest Zone | 0.05 | 0.65 |
|  | Omsk Forest-Steppe Zone | **0.00** | **0.24** |
|  | Predverkhoyanie | 0.12 | 0.85 |
|  | Pur River | 0.36 | 0.94 |
|  | Selenga | 0.07 | 0.78 |
|  | Southern Russian Far East | 0.03 | 0.51 |
|  | West Taimyr | 0.14 | 0.83 |
|  | Yakutia | **0.51** | **0.97** |
| *Sorex araneus* | Altai Mountains | **0.56** | **0.97** |
|  | Buryatia | 0.00 | 0.54 |
|  | Chulym River | 0.45 | 0.95 |
|  | Ket River | **0.52** | **0.96** |
|  | Kuznetsk Alatau | **0.57** | **0.99** |
|  | Ob River Floodplain | 0.10 | 0.85 |
|  | Moscow | 0.20 | 0.87 |
|  | Norway | **0.00** | **0.02** |
|  | Novosibirsk | 0.14 | 0.92 |
|  | Omsk Forest Zone | 0.50 | 0.98 |
|  | Omsk Forest-Steppe Zone | 0.22 | 0.95 |
|  | Pskov | 0.22 | 0.91 |
|  | Pur River | 0.28 | 0.94 |
|  | Selenga | 0.31 | 0.99 |
|  | Slovakia | 0.09 | 0.83 |
|  | Southern Russian Far East | **0.00** | **0.34** |
|  | Tomsk | 0.21 | 0.81 |
|  | Yakutia | **0.71** | **1.00** |

**Supplementary Table S15.** Percentiles (2.5% and 97.5%) of posterior distributions of *dda_sp_* for flea species estimated by the species-host unified models for compound metacommunities in the Altai Mountains, Armenia, Dzungarian Alatau, and Kurgan (Southern Ural Mountains). *dda_sp_* values significantly (based on a 95% credible interval) larger than 0.5 indicate an absence tendency (bold blue font), whereas *dda_sp_* values significantly (based on a 95% credible interval) smaller than 0.5 indicate a presence tendency (bold red font).

| Region | Flea species | Percentile | |
| --- | --- | --- | --- |
|  |  | 2.5% | 97.5% |
| Altai Mountains | *Amalaraeus penicilliger* | 0.00 | 1.00 |
|  | *Ctenophthalmus assimilis* | 0.00 | 0.00 |
|  | *Frontopsylla elata* | 0.04 | 1.00 |
|  | *Hystrichopsylla talpae* | 0.00 | 1.00 |
|  | *Megabothris rectangulatus* | 0.00 | 1.00 |
|  | *Megabothris turbidus* | 0.07 | 1.00 |
|  | *Neopsylla mana* | 0.00 | 0.01 |
|  | *Palaeopsylla soricis* | 0.00 | 0.72 |
| Armenia | *Amphipsylla rossica* | 0.00 | 0.91 |
|  | *Amphipsylla schelkovnikovi* | 0.00 | 0.84 |
|  | *Callopsylla caspia* | 0.02 | 0.94 |
|  | *Callopsylla saxatilis* | 0.01 | 0.96 |
|  | *Ceratophyllus sciurorum* | 0.00 | 0.81 |
|  | *Citellophilus transcaucasicus* | 0.00 | 0.77 |
|  | *Coptopsylla arax* | 0.04 | 1.00 |
|  | *Ctenophthalmus acuminatus* | 0.02 | 0.96 |
|  | *Ctenophthalmus bogatschevi* | 0.03 | 0.98 |
|  | *Ctenophthalmus golovi* | 0.06 | 0.99 |
|  | *Ctenophthalmus proximus* | 0.03 | 0.98 |
|  | *Ctenophthalmus secundus* | 0.02 | 0.97 |
|  | *Ctenophthalmus teres* | 0.02 | 0.97 |
|  | *Frontopsylla elata* | 0.01 | 0.97 |
|  | *Hystrichopsylla talpae* | 0.08 | 1.00 |
|  | *Leptopsylla segnis* | 0.08 | 1.00 |
|  | *Megabothris turbidus* | 0.02 | 0.98 |
|  | *Megabothris walkeri* | 0.01 | 0.95 |
|  | *Mesopsylla apscheronica* | 0.02 | 1.00 |
|  | *Mesopsylla tuschkan* | 0.01 | 0.99 |
|  | *Myoxopsylla jordani* | 0.00 | 0.84 |
|  | *Neopsylla pleskei* | 0.01 | 0.98 |
|  | *Neopsylla setosa* | 0.01 | 0.92 |
|  | *Nosopsyllus consimilis* | 0.00 | 0.71 |
|  | *Nosopsyllus fasciatus* | 0.03 | 0.99 |
|  | *Nosopsyllus iranus* | 0.01 | 0.91 |
|  | *Nosopsyllus mokrzeckyi* | 0.01 | 0.95 |
|  | *Ophthalmopsylla volgensis* | 0.00 | 0.63 |
|  | *Oropsylla ilovaiskii* | 0.00 | 0.92 |
|  | *Paradoxopsyllus hesperius* | 0.01 | 0.95 |
|  | *Rhadinopsylla cedestis* | 0.01 | 0.97 |
|  | *Rhadinopsylla integella* | 0.01 | 0.96 |
|  | *Rhadinopsylla ucrainica* | 0.02 | 0.97 |
|  | *Stenoponia ivanovi* | 0.15 | 1.00 |
|  | *Stenoponia tripectinata* | 0.28 | 1.00 |
|  | *Wagnerina schelkovnikovi* | 0.00 | 0.97 |
|  | *Xenopsylla cheopis* | 0.14 | 1.00 |
|  | *Xenopsylla conformis* | 0.01 | 0.99 |
| Dzungarian Alatau | *Amalaraeus penicilliger* | 0.01 | 0.91 |
|  | *Amphipsylla primaris* | **0.00** | **0.08** |
|  | *Amphipsylla rossica* | 0.44 | 1.00 |
|  | *Amphipsylla sibirica* | **0.98** | **1.00** |
|  | *Ceratophyllus sciurorum* | **0.00** | **0.45** |
|  | *Citellophilus tesquorum* | 0.01 | 0.85 |
|  | *Corrodopsylla birulai* | 0.00 | 0.80 |
|  | *Ctenophthalmus arvalis* | 0.14 | 1.00 |
|  | *Ctenophthalmus assimilis* | **0.00** | **0.14** |
|  | *Frontopsylla elata* | 0.32 | 1.00 |
|  | *Frontopsylla elatoides* | 0.23 | 0.99 |
|  | *Frontopsylla ornata* | **0.85** | **1.00** |
|  | *Frontopsylla protera* | 0.05 | 0.98 |
|  | *Hystrichopsylla talpae* | 0.00 | 0.60 |
|  | *Megabothris rectangulatus* | 0.02 | 0.91 |
|  | *Neopsylla mana* | **0.00** | **0.38** |
|  | *Neopsylla teratura* | 0.01 | 0.92 |
|  | *Nosopsyllus consimilis* | 0.00 | 0.96 |
|  | *Oropsylla silantiewi* | 0.00 | 0.35 |
|  | *Palaeopsylla soricis* | 0.00 | 0.99 |
|  | *Pectinoctenus nemorosa* | 0.32 | 1.00 |
|  | *Rhadinopsylla angusta* | 0.06 | 1.00 |
| Kurgan | *Amalaraeus penicilliger* | **0.00** | **0.01** |
|  | *Amphipsylla kuznetzovi* | **0.93** | **1.00** |
|  | *Amphipsylla sibirica* | 0.05 | 1.00 |
|  | *Corrodopsylla birulai* | **0.00** | **0.32** |
|  | *Ctenophthalmus assimilis* | **0.00** | **0.32** |
|  | *Ctenophthalmus breviatus* | 0.00 | 1.00 |
|  | *Frontopsylla elata* | 0.00 | 0.91 |
|  | *Hystrichopsylla talpae* | 0.00 | 0.98 |
|  | *Megabothris rectangulatus* | 0.00 | 0.84 |
|  | *Megabothris turbidus* | 0.00 | 1.00 |
|  | *Megabothris walkeri* | **0.00** | **0.43** |
|  | *Neopsylla pleskei* | 0.05 | 1.00 |
|  | *Palaeopsylla soricis* | 0.00 | 0.62 |
|  | *Peromyscopsylla bidentata* | 0.02 | 1.00 |
|  | *Peromyscopsylla silvatica* | 0.08 | 1.00 |
|  | *Rhadinopsylla integella* | **0.98** | **1.00** |

**Supplementary Table S16.** Percentiles (2.5% and 97.5%) of posterior distributions of *dda_sp_* for flea species estimated by the species-host unified models for compound metacommunities in Poland, Tomsk-Tyumen (Southeastern Siberia), Turkmenistan, and Tatarstan. *dda_sp_* values significantly (based on a 95% credible interval) larger than 0.5 indicate an absence tendency (bold blue font), whereas *dda_sp_* values significantly (based on a 95% credible interval) smaller than 0.5 indicate a presence tendency (bold red font).

| Region | Flea species | Percentile | |
| --- | --- | --- | --- |
|  |  | 2.5% | 97.5% |
| Poland | *Amalaraeus arvicolae* | 0.15 | 0.99 |
|  | *Amalaraeus penicilliger* | 0.00 | 0.53 |
|  | *Amphipsylla rossica* | 0.01 | 0.85 |
|  | *Atyphloceras nuperus* | **0.86** | **1.00** |
|  | *Ceratophyllus sciurorum* | 0.02 | 0.97 |
|  | *Ctenophthalmus agyrtes* | 0.00 | 0.62 |
|  | *Ctenophthalmus assimilis* | **0.00** | **0.13** |
|  | *Ctenophthalmus bisoctodentatus* | 0.07 | 0.99 |
|  | *Ctenophthalmus congener* | 0.02 | 0.96 |
|  | *Ctenophthalmus obtusus* | 0.20 | 1.00 |
|  | *Ctenophthalmus solutus* | 0.12 | 0.98 |
|  | *Ctenophthalmus uncinatus* | 0.00 | 0.59 |
|  | *Doratopsylla dasycnema* | 0.01 | 0.93 |
|  | *Hystrichopsylla orientalis* | 0.02 | 0.96 |
|  | *Hystrichopsylla talpae* | **0.00** | **0.31** |
|  | *Leptopsylla segnis* | 0.01 | 0.88 |
|  | *Megabothris turbidus* | 0.01 | 0.87 |
|  | *Nosopsyllus fasciatus* | 0.13 | 0.99 |
|  | *Palaeopsylla kohauti* | 0.13 | 1.00 |
|  | *Palaeopsylla soricis* | **0.00** | **0.17** |
|  | *Peromyscopsylla bidentata* | 0.01 | 0.79 |
|  | *Peromyscopsylla fallax* | 0.26 | 1.00 |
|  | *Peromyscopsylla silvatica* | 0.01 | 0.79 |
|  | *Rhadinopsylla integella* | 0.02 | 0.93 |
|  | *Rhadinopsylla pentacantha* | 0.12 | 0.99 |
| Tomsk-Tyumen | *Amalaraeus penicilliger* | 0.02 | 0.86 |
|  | *Amphipsylla sibirica* | **0.98** | **1.00** |
|  | *Catallagia dacenkoi* | **0.98** | **1.00** |
|  | *Catallagia ioffi* | **0.92** | **1.00** |
|  | *Ceratophyllus indages* | **0.00** | **0.10** |
|  | *Corrodopsylla birulai* | **0.00** | **0.01** |
|  | *Ctenophthalmus assimilis* | 0.00 | 0.52 |
|  | *Ctenophthalmus pisticus* | 0.02 | 1.00 |
|  | *Ctenophthalmus uncinatus* | 0.02 | 0.89 |
|  | *Doratopsylla dasycnema* | 0.50 | 1.00 |
|  | *Frontopsylla elata* | 0.23 | 0.99 |
|  | *Hystrichopsylla talpae* | 0.00 | 0.52 |
|  | *Leptopsylla segnis* | 0.01 | 0.95 |
|  | *Megabothris calcarifer* | **0.85** | **1.00** |
|  | *Megabothris rectangulatus* | 0.06 | 0.94 |
|  | *Megabothris turbidus* | **0.63** | **1.00** |
|  | *Megabothris walkeri* | 0.01 | 0.81 |
|  | *Neopsylla acanthina* | **0.89** | **1.00** |
|  | *Neopsylla mana* | 0.00 | 0.82 |
|  | *Neopsylla pleskei* | 0.31 | 1.00 |
|  | *Nosopsyllus fasciatus* | **0.99** | **1.00** |
|  | *Palaeopsylla soricis* | **0.00** | **0.02** |
|  | *Peromyscopsylla silvatica* | **0.00** | **0.40** |
|  | *Rhadinopsylla integella* | **0.97** | **1.00** |
|  | *Rhadinopsylla li* | **0.00** | **0.02** |
| Turkmenistan | *Amphipsylla parthiana* | 0.12 | 1.00 |
|  | *Amphipsylla rossica* | 0.00 | 0.97 |
|  | *Amphipsylla schelkovnikovi* | 0.01 | 0.98 |
|  | *Citellophilus trispinus* | 0.28 | 1.00 |
|  | *Coptopsylla bairamalensis* | 0.34 | 1.00 |
|  | *Coptopsylla lamellifer* | 0.10 | 0.99 |
|  | *Coptopsylla olgae* | 0.45 | 1.00 |
|  | *Ctenophthalmus dolichus* | 0.18 | 1.00 |
|  | *Desertopsylla rothschildi* | 0.06 | 1.00 |
|  | *Echidnophaga oschanini* | 0.02 | 0.98 |
|  | *Frontopsylla macrophthalma* | **0.66** | **1.00** |
|  | *Leptopsylla taschenbergi* | 0.00 | 0.71 |
|  | *Mesopsylla eucta* | 0.00 | 0.74 |
|  | *Neopsylla setosa* | 0.02 | 0.96 |
|  | *Nosopsyllus consimilis* | 0.07 | 0.99 |
|  | *Nosopsyllus fidus* | 0.26 | 1.00 |
|  | *Nosopsyllus laeviceps* | 0.04 | 0.98 |
|  | *Nosopsyllus philippovi* | **0.55** | **1.00** |
|  | *Nosopsyllus tersus* | **0.82** | **1.00** |
|  | *Nosopsyllus turkmenicus* | 0.18 | 1.00 |
|  | *Ophthalmopsylla karakum* | **0.72** | **1.00** |
|  | *Ophthalmopsylla volgensis* | 0.16 | 1.00 |
|  | *Paradoxopsyllus repandus* | **0.62** | **1.00** |
|  | *Paradoxopsyllus teretifrons* | **0.58** | **1.00** |
|  | *Rhadinopsylla cedestis* | 0.22 | 1.00 |
|  | *Rostropsylla daca* | **0.52** | **1.00** |
|  | *Stenoponia vlasovi* | 0.00 | 0.94 |
|  | *Wagnerina schelkovnikovi* | **0.84** | **1.00** |
|  | *Xenopsylla conformis* | 0.00 | 0.09 |
|  | *Xenopsylla gerbilli* | 0.00 | 0.68 |
|  | *Xenopsylla hirtipes* | 0.05 | 0.99 |
|  | *Xenopsylla nuttalli* | 0.07 | 0.98 |
|  | *Xenopsylla persica* | 0.04 | 0.99 |
| Tatarstan | *Amalaraeus penicilliger* | **0.00** | **0.33** |
|  | *Amphipsylla rossica* | **0.00** | **0.44** |
|  | *Ceratophyllus sciurorum* | 0.44 | 0.99 |
|  | *Citellophilus tesquorum* | 0.06 | 0.93 |
|  | *Corrodopsylla birulai* | 0.04 | 0.90 |
|  | *Ctenophthalmus agyrtes* | **0.00** | **0.38** |
|  | *Ctenophthalmus assimilis* | **0.00** | **0.08** |
|  | *Ctenophthalmus bisoctodentatus* | 0.25 | 0.99 |
|  | *Ctenophthalmus breviatus* | 0.08 | 0.93 |
|  | *Ctenophthalmus orientalis* | **0.63** | **1.00** |
|  | *Ctenophthalmus uncinatus* | **0.01** | **0.49** |
|  | *Ctenophthalmus wagneri* | 0.04 | 0.81 |
|  | *Doratopsylla dasycnema* | 0.02 | 0.89 |
|  | *Frontopsylla semura* | **0.85** | **1.00** |
|  | *Hystrichopsylla talpae* | **0.00** | **0.09** |
|  | *Leptopsylla segnis* | **0.79** | **1.00** |
|  | *Leptopsylla taschenbergi* | 0.09 | 0.96 |
|  | *Megabothris rectangulatus* | **0.00** | **0.39** |
|  | *Megabothris turbidus* | **0.00** | **0.17** |
|  | *Megabothris walkeri* | 0.16 | 0.97 |
|  | *Neopsylla pleskei* | **0.00** | **0.21** |
|  | *Neopsylla setosa* | 0.24 | 0.98 |
|  | *Nosopsyllus consimilis* | **0.88** | **1.00** |
|  | *Nosopsyllus fasciatus* | **0.91** | **1.00** |
|  | *Oropsylla ilovaiskii* | **0.64** | **1.00** |
|  | *Palaeopsylla kohauti* | 0.39 | 1.00 |
|  | *Palaeopsylla soricis* | 0.00 | 0.12 |
|  | *Peromyscopsylla bidentata* | 0.05 | 0.91 |
|  | *Peromyscopsylla silvatica* | 0.06 | 0.91 |
|  | *Rhadinopsylla integella* | 0.09 | 0.96 |

**Supplementary Table S17**. Percentiles (2.5% and 97.5%) of posterior distributions of *dda_host_* for host species estimated by the species-host unified models for flea compound metacommunities. *dda_host_* values significantly (based on a 95% credible interval) larger than 0.5 indicate an absence tendency (bold blue font), whereas *dda_host_* values significantly (based on a 95% credible interval) smaller than 0.5 indicate a presence tendency (bold red font).

| Region | Host species | Percentile | |
| --- | --- | --- | --- |
|  |  | 2.5% | 97.5% |
| Altai Mountains | *Apodemus agrarius* | **0.00** | **0.01** |
|  | *Apodemus speciosus* | **0.00** | **0.02** |
|  | *Arvicola amphibius* | 0.00 | 1.00 |
|  | *Microtus agrestis* | 0.00 | 1.00 |
|  | *Microtus arvalis* | 0.00 | 0.40 |
|  | *Microtus gregalis* | 0.00 | 1.00 |
|  | *Microtus oeconomus* | 0.00 | 1.00 |
|  | *Myodes glareolus* | 0.00 | 0.16 |
|  | *Myodes rutilus* | 0.00 | 0.03 |
|  | *Neomys fodiens* | 0.00 | 1.00 |
|  | *Sicista subtilis* | 0.00 | 1.00 |
|  | *Sorex araneus* | 0.00 | 1.00 |
|  | *Sorex arcticus* | 0.01 | 1.00 |
|  | *Sorex caecutiens* | 0.03 | 1.00 |
|  | *Sorex minutus* | 0.00 | 1.00 |
| Armenia | *Apodemus agrarius* | 0.23 | 1.00 |
|  | *Apodemus witherbyi* | **0.00** | **0.23** |
|  | *Arvicola amphibius* | 0.00 | 0.91 |
|  | *Chionomys nivalis* | 0.17 | 1.00 |
|  | *Cricetulus migratorius* | **0.00** | **0.30** |
|  | *Crocidura suaveolens* | 0.05 | 1.00 |
|  | *Dryomys nitedula* | 0.21 | 1.00 |
|  | *Meriones persicus* | **0.00** | **0.07** |
|  | *Meriones tristrami* | 0.00 | 0.98 |
|  | *Meriones vinogradovi* | 0.01 | 0.99 |
|  | *Mesocricetus brandti* | 0.00 | 0.61 |
|  | *Microtus arvalis* | **0.00** | **0.39** |
|  | *Microtus schidlovskii* | **0.77** | **1.00** |
|  | *Microtus socialis* | 0.12 | 1.00 |
|  | *Neomys teres* | **0.92** | **1.00** |
|  | *Spermophilus xanthoprymnus* | 0.02 | 0.99 |
| Dzungarian Alatau | *Apodemus agrarius* | 0.00 | 0.70 |
|  | *Apodemus uralensis* | **0.00** | **0.11** |
|  | *Arvicola amphibius* | 0.01 | 0.98 |
|  | *Cricetulus migratorius* | 0.12 | 1.00 |
|  | *Cricetus cricetus* | 0.49 | 1.00 |
|  | *Dryomys nitedula* | 0.00 | 0.95 |
|  | *Marmota baibacina* | 0.00 | 1.00 |
|  | *Microtus arvalis* | **0.00** | **0.01** |
|  | *Myodes centralis* | **0.00** | **0.02** |
|  | *Neomys fodiens* | 0.00 | 0.99 |
|  | *Sicista tianshanica* | 0.00 | 0.77 |
|  | *Sorex araneus* | 0.01 | 0.99 |
|  | *Spermophilus erythrogenys* | **0.77** | **1.00** |
|  | *Urocitellus undulatus* | 0.01 | 0.96 |
| Kurgan | *Apodemus agrarius* | 0.01 | 1.00 |
|  | *Apodemus uralensis* | 0.00 | 0.97 |
|  | *Craseomys rufocanus* | 0.00 | 0.73 |
|  | *Micromys minutus* | 0.04 | 1.00 |
|  | *Microtus agrestis* | 0.00 | 1.00 |
|  | *Microtus arvalis* | 0.00 | 0.61 |
|  | *Microtus gregalis* | 0.00 | 0.17 |
|  | *Microtus oeconomus* | 0.00 | 0.96 |
|  | *Neomys fodiens* | 0.05 | 1.00 |
|  | *Sicista betulina* | 0.00 | 1.00 |
|  | *Sorex araneus* | **0.00** | **0.35** |
|  | *Sorex caecutiens* | 0.06 | 1.00 |
|  | *Sorex minutus* | 0.04 | 1.00 |
|  | *Sorex tundrensis* | 0.05 | 1.00 |
| Poland | *Apodemus agrarius* | 0.00 | 0.92 |
|  | *Apodemus flavicollis* | **0.00** | **0.30** |
|  | *Apodemus sylvaticus* | 0.01 | 0.95 |
|  | *Apodemus uralensis* | 0.31 | 1.00 |
|  | *Crocidura suaveolens* | **0.93** | **1.00** |
|  | *Microtus agrestis* | **0.00** | **0.19** |
|  | *Microtus arvalis* | 0.00 | 0.73 |
|  | *Microtus subterraneus* | 0.00 | 0.54 |
|  | *Muscardinus avellanarius* | **0.92** | **1.00** |
|  | *Myodes glareolus* | **0.00** | **0.01** |
|  | *Neomys anomalus* | **0.65** | **1.00** |
|  | *Neomys fodiens* | 0.07 | 0.99 |
|  | *Sorex alpinus* | 0.33 | 1.00 |
|  | *Sorex araneus* | 0.00 | 0.85 |
|  | *Sorex minutus* | **0.82** | **1.00** |
| Tomsk-Tyumen | *Apodemus agrarius* | **0.00** | **0.18** |
|  | *Apodemus peninsulae* | 0.01 | 0.94 |
|  | *Arvicola amphibius* | 0.00 | 0.90 |
|  | *Craseomys rufocanus* | 0.00 | 0.79 |
|  | *Cricetus cricetus* | 0.42 | 1.00 |
|  | *Micromys minutus* | 0.11 | 1.00 |
|  | *Microtus agrestis* | **0.00** | **0.36** |
|  | *Microtus arvalis* | **0.00** | **0.07** |
|  | *Microtus oeconomus* | **0.00** | **0.49** |
|  | *Myodes glareolus* | **0.00** | **0.02** |
|  | *Myodes rutilus* | **0.00** | **0.09** |
|  | *Neomys fodiens* | 0.35 | 1.00 |
|  | *Sicista betulina* | 0.23 | 1.00 |
|  | *Sorex araneus* | 0.00 | 0.73 |
|  | *Sorex arcticus* | 0.08 | 1.00 |
|  | *Sorex caecutiens* | 0.00 | 0.91 |
|  | *Sorex isodon* | 0.20 | 0.99 |
|  | *Sorex minutus* | 0.24 | 1.00 |
|  | *Sorex roboratus* | 0.29 | 1.00 |
|  | *Tamias sibiricus* | 0.04 | 1.00 |
| Turkmenistan | *Allactaga severtzovi* | 0.42 | 1.00 |
|  | *Apodemus uralensis* | 0.14 | 1.00 |
|  | *Blanfordimys afghanus* | 0.00 | 0.74 |
|  | *Cricetulus migratorius* | 0.00 | 0.34 |
|  | *Dipus sagitta* | **0.00** | **0.20** |
|  | *Eremodipus lichtensteini* | **0.54** | **1.00** |
|  | *Meriones libycus* | **0.00** | **0.02** |
|  | *Meriones meridianus* | **0.00** | **0.01** |
|  | *Microtus socialis* | 0.02 | 0.99 |
|  | *Microtus transcaspicus* | 0.22 | 1.00 |
|  | *Nesokia indica* | 0.04 | 0.98 |
|  | *Pygeretmus pumilio* | 0.27 | 1.00 |
|  | *Rhombomys opimus* | **0.00** | **0.10** |
|  | *Spermophilopsis leptodactylus* | **0.00** | **0.07** |
|  | *Spermophilus fulvus* | **0.00** | **0.19** |
| Tatarstan | *Apodemus agrarius* | 0.14 | 0.99 |
|  | *Apodemus flavicollis* | **0.00** | **0.69** |
|  | *Apodemus uralensis* | **0.00** | **0.36** |
|  | *Arvicola amphibius* | 0.10 | 0.98 |
|  | *Dryomys nitedula* | **0.99** | **1.00** |
|  | *Microtus agrestis* | 0.09 | 0.99 |
|  | *Microtus arvalis* | **0.00** | **0.50** |
|  | *Microtus oeconomus* | 0.21 | 0.99 |
|  | *Myodes glareolus* | **0.00** | **0.01** |
|  | *Myodes rutilus* | 0.02 | 0.96 |
|  | *Neomys fodiens* | **0.60** | **1.00** |
|  | *Sicista betulina* | 0.14 | 0.99 |
|  | *Sorex araneus* | **0.00** | **0.34** |
|  | *Sorex minutus* | 0.16 | 0.99 |
|  | *Spermophilus major* | **0.00** | **0.06** |
|  | *Spermophilus pygmaeus* | **0.00** | **0.18** |
|  | *Talpa europaea* | 0.03 | 0.95 |

**Supplementary Table S18.** Percentiles (2.5% and 97.5%) of posterior distributions of *dda_sp_* for mite species estimated by the species-host unified models for compound metacommunities in the Chulym River (Khakassia), Krasnodar (Southwestern Russia), and Northern Russian Far East. *dda_sp_* values significantly (based on a 95% credible interval) larger than 0.5 indicate an absence tendency (bold blue font), whereas *dda_sp_* values significantly (based on a 95% credible interval) smaller than 0.5 indicate a presence tendency (bold red font).

| Region | Mite species | Percentiles | |
| --- | --- | --- | --- |
|  |  | 2.5% | 97.5% |
| Chulym River | *Androlaelaps casalis* | 0.04 | 1.00 |
|  | *Androlaelaps glasgowi* | 0.00 | 0.99 |
|  | *Eulaelaps stabularis* | **0.00** | **0.06** |
|  | *Haemogamasus ambulans* | **0.00** | **0.06** |
|  | *Haemogamasus nidi* | 0.00 | 0.97 |
|  | *Haemogamasus nidiformes* | 0.02 | 1.00 |
|  | *Hirstionyssus apodemi* | 0.13 | 1.00 |
|  | *Hirstionyssus eusoricis* | 0.00 | 0.97 |
|  | *Hirstionyssus isabellinus* | **0.00** | **0.33** |
|  | *Hyperlaelaps arvalis* | 0.42 | 1.00 |
|  | *Laelaps clethrionomydis* | 0.00 | 1.00 |
|  | *Laelaps hilaris* | 0.02 | 1.00 |
|  | *Laelaps muris* | 0.01 | 1.00 |
|  | *Laelaps pavlovskyi* | 0.00 | 0.99 |
| Krasnodar | *Androlaelaps glasgowi* | 0.00 | 0.98 |
|  | *Androlaelaps karawajevi* | **0.96** | **1.00** |
|  | *Androlaelaps sardous* | **0.97** | **1.00** |
|  | *Androlaelaps semidesertus* | **0.97** | **1.00** |
|  | *Eulaelaps stabularis* | 0.00 | 0.00 |
|  | *Haemogamasus hirsutosimilis* | 0.00 | 1.00 |
|  | *Haemogamasus hirsutus* | 0.01 | 1.00 |
|  | *Haemogamasus nidi* | **0.00** | **0.05** |
|  | *Hirstionyssus apodemi* | 0.03 | 1.00 |
|  | *Hirstionyssus carnifex* | **0.85** | **1.00** |
|  | *Hirstionyssus criceti* | 0.00 | 0.86 |
|  | *Hirstionyssus eusoricis* | 0.02 | 1.00 |
|  | *Hirstionyssus isabellinus* | 0.01 | 1.00 |
|  | *Hirstionyssus talpae* | 0.44 | 1.00 |
|  | *Hyperlaelaps arvalis* | **0.63** | **1.00** |
|  | *Laelaps agilis* | 0.03 | 1.00 |
|  | *Laelaps algericus* | 0.40 | 1.00 |
|  | *Laelaps hilaris* | 0.00 | 0.99 |
|  | *Laelaps jettmari* | **0.94** | **1.00** |
|  | *Laelaps micromydis* | **0.99** | **1.00** |
|  | *Laelaps muris* | 0.00 | 0.94 |
|  | *Laelaps pavlovskyi* | 0.49 | 1.00 |
|  | *Laelaps pitymidis* | **0.89** | **1.00** |
|  | *Myonyssus rossicus* | 0.01 | 1.00 |
| Northern Russian Far East | *Androlaelaps casalis* | 0.33 | 1.00 |
|  | *Androlaelaps pavlovskii* | 0.06 | 1.00 |
|  | *Eulaelaps stabularis* | 0.00 | 0.96 |
|  | *Haemogamasus ambulans* | 0.00 | 0.21 |
|  | *Haemogamasus dauricus* | **0.62** | **1.00** |
|  | *Haemogamasus liponyssoides* | 0.00 | 0.68 |
|  | *Haemogamasus mandshuricus* | 0.00 | 0.98 |
|  | *Haemogamasus nidi* | 0.00 | 0.89 |
|  | *Haemogamasus serdjukovae* | 0.01 | 1.00 |
|  | *Hirstionyssus criceti* | 0.00 | 0.99 |
|  | *Hirstionyssus eusoricis* | 0.00 | 0.03 |
|  | *Hirstionyssus isabellinus* | 0.00 | 0.01 |
|  | *Hirstionyssus latiscutatus* | 0.45 | 1.00 |
|  | *Hirstionyssus pavlovskyi* | **0.55** | **1.00** |
|  | *Hyperlaelaps arvalis* | 0.00 | 0.98 |
|  | *Laelaps clethrionomydis* | 0.00 | 0.51 |
|  | *Laelaps lemmi* | 0.00 | 1.00 |
|  | *Laelaps semitectus* | **0.90** | **1.00** |

**Supplementary Table S19**. Percentiles (2.5% and 97.5%) of posterior distributions of *dda_sp_* for mite species estimated by the species-host unified model for compound metacommunities in Novosibirsk (Southwestern Siberia), Omsk Forest-Steppe Zone (Southwestern Siberia), and Southern Russian Far East. *dda_sp_* values significantly (based on a 95% credible interval) larger than 0.5 indicate an absence tendency (bold blue font), whereas *dda_sp_* values significantly (based on a 95% credible interval) smaller than 0.5 indicate a presence tendency (bold red font).

| Region | Mite species | Percentile | |
| --- | --- | --- | --- |
|  |  | 2.5% | 97.5% |
| Novosibirsk | *Androlaelaps glasgowi* | **0.00** | **0.28** |
|  | *Eulaelaps stabularis* | **0.00** | **0.01** |
|  | *Haemogamasus ambulans* | **0.00** | **0.00** |
|  | *Haemogamasus mandshuricus* | **0.00** | **0.03** |
|  | *Haemogamasus nidi* | 0.06 | 0.96 |
|  | *Haemogamasus nidiformes* | **0.80** | **1.00** |
|  | *Hirstionyssus apodemi* | **0.73** | **1.00** |
|  | *Hirstionyssus criceti* | **0.89** | **1.00** |
|  | *Hirstionyssus eusoricis* | **0.00** | **0.11** |
|  | *Hirstionyssus isabellinus* | **0.00** | **0.01** |
|  | *Hyperlaelaps arvalis* | **0.52** | **1.00** |
|  | *Laelaps clethrionomydis* | **0.00** | **0.04** |
|  | *Laelaps hilaris* | 0.11 | 0.98 |
|  | *Laelaps micromydis* | **0.97** | **1.00** |
|  | *Laelaps muris* | 0.20 | 0.99 |
|  | *Laelaps pavlovskyi* | 0.02 | 0.81 |
|  | *Myonyssus ingricus* | 0.03 | 0.98 |
| Omsk Forest-Steppe Zone | *Androlaelaps casalis* | **0.82** | **1.00** |
|  | *Androlaelaps glasgowi* | **0.00** | **0.01** |
|  | *Eulaelaps stabularis* | **0.00** | **0.09** |
|  | *Haemogamasus ambulans* | **0.00** | **0.25** |
|  | *Haemogamasus mandshuricus* | 0.06 | 0.99 |
|  | *Haemogamasus nidi* | 0.03 | 0.95 |
|  | *Haemogamasus nidiformes* | 0.28 | 1.00 |
|  | *Hirstionyssus apodemi* | 0.02 | 0.89 |
|  | *Hirstionyssus criceti* | 0.32 | 1.00 |
|  | *Hirstionyssus eusoricis* | **0.00** | **0.04** |
|  | *Hirstionyssus isabellinus* | **0.00** | **0.05** |
|  | *Hirstionyssus latiscutatus* | **0.97** | **1.00** |
|  | *Hirstionyssus transiliensis* | 0.01 | 0.86 |
|  | *Hyperlaelaps amphibius* | 0.74 | 1.00 |
|  | *Hyperlaelaps arvalis* | **0.00** | **0.41** |
|  | *Laelaps agilis* | 0.00 | 0.81 |
|  | *Laelaps algericus* | 0.06 | 0.98 |
|  | *Laelaps clethrionomydis* | **0.00** | **0.01** |
|  | *Laelaps hilaris* | **0.00** | **0.33** |
|  | *Laelaps micromydis* | **0.98** | **1.00** |
|  | *Laelaps multispinosus* | **0.00** | **0.46** |
|  | *Laelaps muris* | **0.00** | **0.49** |
|  | *Laelaps pavlovskyi* | 0.02 | 0.83 |
| Northern Russian Far East | *Androlaelaps casalis* | 0.07 | 0.95 |
|  | *Androlaelaps glasgowi* | **0.00** | **0.40** |
|  | *Androlaelaps razumovae* | 0.20 | 0.99 |
|  | *Eulaelaps cricetuli* | 0.23 | 0.99 |
|  | *Eulaelaps kolpakovae* | 0.21 | 0.99 |
|  | *Eulaelaps stabularis* | 0.00 | 0.60 |
|  | *Haemogamasus ambulans* | **0.00** | **0.07** |
|  | *Haemogamasus dauricus* | 0.07 | 0.95 |
|  | *Haemogamasus horridus* | 0.02 | 0.86 |
|  | *Haemogamasus liponyssoides* | 0.00 | 0.53 |
|  | *Haemogamasus mandshuricus* | **0.00** | **0.24** |
|  | *Haemogamasus nidi* | 0.02 | 0.88 |
|  | *Haemogamasus nidiformes* | 0.08 | 0.96 |
|  | *Haemogamasus serdjukovae* | 0.05 | 0.91 |
|  | *Hirstionyssus bregetovae* | **0.79** | **1.00** |
|  | *Hirstionyssus criceti* | 0.05 | 0.99 |
|  | *Hirstionyssus eusoricis* | **0.00** | **0.35** |
|  | *Hirstionyssus eversmanni* | 0.12 | 0.98 |
|  | *Hirstionyssus isabellinus* | **0.00** | **0.17** |
|  | *Hirstionyssus sciurinus* | **0.77** | **1.00** |
|  | *Hyperlaelaps arvalis* | 0.01 | 0.81 |
|  | *Laelaps agilis* | **0.00** | **0.41** |
|  | *Laelaps clethrionomydis* | **0.00** | **0.12** |
|  | *Laelaps lemmi* | 0.06 | 0.94 |
|  | *Laelaps micromydis* | 0.53 | 1.00 |
|  | *Laelaps muris* | 0.01 | 0.91 |
|  | *Laelaps nuttalli* | 0.00 | 0.60 |
|  | *Laelaps pavlovskyi* | **0.00** | **0.40** |
|  | *Myonyssus dubinini* | 0.02 | 0.83 |

**Supplementary Table S20**. Percentiles (2.5% and 97.5%) of posterior distributions of *dda_host_* for host species estimated by the species-host unified models for mite compound metacommunities. *dda_host_* values significantly (based on a 95% credible interval) larger than 0.5 indicate an absence tendency (bold blue font), whereas *dda_host_* values significantly (based on a 95% credible interval) smaller than 0.5 indicate a presence tendency (bold red font).

| Region | Host species | Percentile | |
| --- | --- | --- | --- |
|  |  | 2.5% | 97.5% |
| Chulym River | *Apodemus peninsulae* | 0.00 | 0.93 |
|  | *Arvicola amphibius* | 0.00 | 0.99 |
|  | *Craseomys rufocanus* | 0.00 | 0.92 |
|  | *Micromys minutus* | 0.00 | 1.00 |
|  | *Microtus arvalis* | 0.00 | 0.82 |
|  | *Microtus gregalis* | 0.00 | 1.00 |
|  | *Microtus oeconomus* | 0.00 | 0.48 |
|  | *Myodes glareolus* | 0.00 | 0.82 |
|  | *Myodes rutilus* | 0.00 | 0.30 |
|  | *Sorex araneus* | 0.05 | 1.00 |
|  | *Talpa altaica* | **0.92** | **1.00** |
|  | *Tamias sibiricus* | 0.39 | 1.00 |
| Krasnodar | *Apodemus agrarius* | 0.00 | 0.72 |
|  | *Apodemus flavicollis* | 0.00 | 0.72 |
|  | *Apodemus sylvaticus* | **0.00** | **0.32** |
|  | *Arvicola amphibius* | **0.00** | **0.05** |
|  | *Cricetulus migratorius* | **0.00** | **0.03** |
|  | *Cricetus cricetus* | 0.01 | 1.00 |
|  | *Crocidura suaveolens* | 0.00 | 0.99 |
|  | *Mesocricetus brandti* | 0.03 | 1.00 |
|  | *Micromys minutus* | 0.00 | 0.99 |
|  | *Microtus arvalis* | **0.00** | **0.02** |
|  | *Microtus majori* | 0.00 | 0.95 |
|  | *Sicista subtilis* | **0.98** | **1.00** |
|  | *Spermophilus pygmaeus* | 0.01 | 1.00 |
|  | *Talpa europaea* | **0.00** | **0.15** |
| Northern Russian Far East | *Alticola macrotis* | 0.04 | 1.00 |
|  | *Craseomys rufocanus* | 0.00 | 0.97 |
|  | *Dicrostonyx torquatus* | 0.00 | 1.00 |
|  | *Lemmus sibiricus* | 0.00 | 1.00 |
|  | *Microtus oeconomus* | 0.00 | 0.45 |
|  | *Myodes rutilus* | 0.00 | 0.04 |
|  | *Ochotona hyperborea* | 0.15 | 1.00 |
|  | *Sorex caecutiens* | **0.92** | **1.00** |
|  | *Sorex daphaenodon* | 0.10 | 1.00 |
|  | *Tamias sibiricus* | 0.00 | 0.93 |
|  | *Urocitellus parryi* | 0.01 | 1.00 |
| Novosibirsk | *Apodemus agrarius* | 0.00 | 0.70 |
|  | *Apodemus peninsulae* | 0.04 | 1.00 |
|  | *Arvicola amphibius* | 0.00 | 0.89 |
|  | *Craseomys rufocanus* | 0.29 | 1.00 |
|  | *Cricetus cricetus* | 0.33 | 1.00 |
|  | *Micromys minutus* | 0.03 | 1.00 |
|  | *Microtus agrestis* | **0.00** | **0.50** |
|  | *Microtus arvalis* | **0.00** | **0.10** |
|  | *Microtus gregalis* | **0.00** | **0.24** |
|  | *Microtus oeconomus* | 0.00 | 0.82 |
|  | *Myodes glareolus* | 0.03 | 0.99 |
|  | *Myodes rutilus* | 0.01 | 0.96 |
|  | *Neomys fodiens* | 0.27 | 1.00 |
|  | *Sicista betulina* | 0.07 | 1.00 |
|  | *Sorex araneus* | 0.01 | 0.94 |
|  | *Sorex caecutiens* | 0.50 | 1.00 |
|  | *Sorex isodon* | **0.60** | **1.00** |
|  | *Sorex minutus* | 0.02 | 0.98 |
|  | *Sorex tundrensis* | 0.26 | 1.00 |
|  | *Talpa altaica* | **0.66** | **1.00** |
|  | *Tamias sibiricus* | **0.62** | **1.00** |
| Omsk Forest-Steppe Zone | *Apodemus agrarius* | 0.00 | 0.66 |
|  | *Apodemus sylvaticus* | 0.06 | 1.00 |
|  | *Arvicola amphibius* | 0.00 | 0.92 |
|  | *Craseomys rufocanus* | 0.24 | 1.00 |
|  | *Cricetus cricetus* | 0.02 | 0.99 |
|  | *Micromys minutus* | 0.13 | 1.00 |
|  | *Microtus agrestis* | 0.05 | 0.99 |
|  | *Microtus arvalis* | 0.00 | 0.91 |
|  | *Microtus gregalis* | **0.00** | **0.04** |
|  | *Microtus oeconomus* | 0.01 | 0.94 |
|  | *Myodes glareolus* | 0.16 | 1.00 |
|  | *Myodes rutilus* | 0.00 | 0.10 |
|  | *Sicista betulina* | 0.40 | 1.00 |
|  | *Sorex araneus* | 0.03 | 0.98 |
|  | *Sorex arcticus* | 0.20 | 1.00 |
|  | *Sorex caecutiens* | 0.22 | 1.00 |
|  | *Sorex daphaenodon* | 0.33 | 1.00 |
|  | *Sorex minutus* | **0.75** | **1.00** |
| Southern Russian Far East | *Apodemus agrarius* | **0.00** | **0.35** |
|  | *Apodemus peninsulae* | 0.00 | 0.78 |
|  | *Craseomys rufocanus* | **0.00** | **0.09** |
|  | *Cricetulus barabensis* | 0.01 | 0.98 |
|  | *Micromys minutus* | 0.03 | 0.96 |
|  | *Microtus fortis* | 0.03 | 0.96 |
|  | *Microtus maximowiczii* | **0.72** | **1.00** |
|  | *Myodes rutilus* | 0.46 | 1.00 |
|  | *Myopus schisticolor* | 0.39 | 1.00 |
|  | *Ochotona alpina* | **0.85** | **1.00** |
|  | *Sciurus vulgaris* | **0.75** | **1.00** |
|  | *Sorex araneus* | 0.01 | 0.89 |
|  | *Tamias sibiricus* | 0.05 | 0.97 |
|  | *Urocitellus undulatus* | 0.33 | 1.00 |

**Supplementary Table S21**. Estimated parameters of posterior distributions of logistic regression coefficients (percentiles 2.5% and 97.5%) for the relationships between the dark diversity affinity of parasite species (*dda_sp_*) and their traits from the species-region unified models for flea and mite component metacommunities (i.e., within a host species across regions). Multiple class-specific intercepts were estimated for parameters of categorical variables with more than one density distribution. Traits are as follows. Ab: characteristic abundance; HN: number of host species across geographic range; PD: phylogenetic diversity of these hosts; BS: body size; SD: sexual size dimorphism; MHpref: microhabitat preference (for fleas) (1: host’s hair; 2: no clear preference; 3: host’s nest); Combs: possession and number of sclerotized combs (for fleas) (1: no combs, 2: one comb; 3: two combs); Feed: feeding mode (for mites) (1: facultative haematophage, 2: obligatory exclusive haematophage, 3: obligatory non-exclusive haematophage). Bold font: significantly positive or significantly negative coefficients.

| Parasite | Host species | Trait | Percentile | |
| --- | --- | --- | --- | --- |
|  |  |  | 2.5% | 97.% |
| Fleas | *Apodemus agrarius* | Ab | -3.14 | 0.18 |
|  |  | HN | **-6.97** | **-2.67** |
|  |  | PD | -0.22 | 2.90 |
|  |  | BS | -2.60 | 1.07 |
|  |  | SD | -1.79 | 1.42 |
|  |  | MHpref1 | -1.11 | 1.27 |
|  |  | MHpref2 | -0.78 | 2.91 |
|  |  | MHpref3 | -2.68 | 0.55 |
|  |  | Combs1 | - | - |
|  |  | Combs2 | -2.60 | -0.04 |
|  |  | Combs3 | **0.04** | **2.60** |
|  | *Apodemus uralensis* | Ab | -2.37 | 0.42 |
|  |  | HN | **-6.73** | **-3.32** |
|  |  | PD | -1.06 | 1.31 |
|  |  | BS | -0.75 | 2.31 |
|  |  | SD | -1.25 | 1.24 |
|  |  | MHpref1 | -1.07 | 0.62 |
|  |  | MHpref2 | -1.24 | 1.17 |
|  |  | MHpref3 | -0.82 | 1.45 |
|  |  | Combs1 | -0.55 | 1.10 |
|  |  | Combs2 | -1.10 | 0.55 |
|  |  | Combs3 | -2.37 | 0.42 |
|  | *Cricetulus migratorius* | Ab | **-4.21** | **-0.33** |
|  |  | HN | -4.32 | 0.13 |
|  |  | PD | -1.13 | 2.17 |
|  |  | BS | -2.71 | 1.96 |
|  |  | SD | **-3.76** | **-0.16** |
|  |  | MHpref1 | **0.22** | **3.02** |
|  |  | MHpref2 | -3.47 | 0.23 |
|  |  | MHpref3 | -1.68 | 1.56 |
|  |  | Combs1 | -0.55 | 4.67 |
|  |  | Combs2 | -3.10 | 0.37 |
|  |  | Combs3 | -2.43 | 0.84 |
|  | *Microtus arvalis* | Ab | **-3.80** | **-0.60** |
|  |  | HN | **-8.44** | **-4.70** |
|  |  | PD | -1.16 | 1.28 |
|  |  | BS | **-5.89** | **-0.63** |
|  |  | SD | **-2.83** | **-0.10** |
|  |  | MHpref1 | -1.06 | 0.91 |
|  |  | MHpref2 | -2.20 | 0.87 |
|  |  | MHpref3 | -0.77 | 2.23 |
|  |  | Combs1 | - | - |
|  |  | Combs2 | -0.51 | 1.49 |
|  |  | Combs3 | -1.49 | 0.51 |
|  | *Microtus oeconomus* | Ab | -3.26 | 0.84 |
|  |  | HN | **-6.18** | **-0.43** |
|  |  | PD | -2.17 | 1.96 |
|  |  | BS | -2.62 | 1.30 |
|  |  | SD | -0.46 | 3.64 |
|  |  | MHpref1 | -3.56 | 0.43 |
|  |  | MHpref2 | -3.46 | 2.73 |
|  |  | MHpref3 | -0.37 | 4.16 |
|  |  | Combs1 | -3.91 | 0.92 |
|  |  | Combs2 | -0.92 | 3.91 |
|  |  | Combs3 | - | - |
|  | *Myodes rutilus* | Ab | **0.70** | **3.44** |
|  |  | HN | **-10.45** | **-4.83** |
|  |  | PD | **-2.70** | **-0.01** |
|  |  | BS | -1.68 | 1.25 |
|  |  | SD | -0.51 | 2.26 |
|  |  | MHpref1 | -0.70 | 1.29 |
|  |  | MHpref2 | **-2.82** | **-0.20** |
|  |  | MHpref3 | -0.46 | 2.97 |
|  |  | Combs1 | - | - |
|  |  | Combs2 | **0.06** | **2.25** |
|  |  | Combs3 | **-2.25** | **-0.06** |
|  | *Sorex araneus* | Ab | **-3.24** | **-0.43** |
|  |  | HN | **-6.45** | **-3.56** |
|  |  | PD | -2.51 | 0.17 |
|  |  | BS | -0.85 | 1.43 |
|  |  | SD | -1.18 | 1.38 |
|  |  | MHpref1 | -2.22 | -0.46 |
|  |  | MHpref2 | -0.93 | 1.86 |
|  |  | MHpref3 | -0.31 | 2.15 |
|  |  | Combs1 | - | - |
|  |  | Combs2 | -0.55 | 1.40 |
|  |  | Combs3 | -1.40 | 0.55 |
| Mites | *Craseomys rufocanus* | Ab | -1.71 | 2.70 |
|  |  | HN | **-11.13** | **-4.67** |
|  |  | PD | **0.10** | **6.44** |
|  |  | BS | -1.87 | 3.06 |
|  |  | SD | -5.14 | 0.39 |
|  |  | Feed1 | -1.60 | 2.21 |
|  |  | Feed2 | -1.92 | 1.77 |
|  |  | Feed3 | -1.80 | 1.26 |
|  | *Microtus oeconomus* | Ab | -0.30 | 3.45 |
|  |  | HN | **-9.14** | **-4.96** |
|  |  | PD | **1.51** | **5.54** |
|  |  | BS | -1.53 | 2.75 |
|  |  | SD | -2.52 | 2.16 |
|  |  | Feed1 | -3.30 | 0.19 |
|  |  | Feed2 | **1.52** | **5.16** |
|  |  | Feed3 | **-3.25** | **-0.26** |
|  | *Myodes glareolus* | Ab | **-15.78** | **-0.35** |
|  |  | HN | **-8.42** | **-0.91** |
|  |  | PD | -1.33 | 3.98 |
|  |  | BS | -2.76 | 2.60 |
|  |  | SD | -4.60 | 0.03 |
|  |  | Feed1 | -2.26 | 1.47 |
|  |  | Feed2 | -1.91 | 1.01 |
|  |  | Feed3 | -0.94 | 2.62 |
|  | *Myodes rutilus* | Ab | -3.66 | 0.07 |
|  |  | HN | **-8.70** | **-4.95** |
|  |  | PD | -1.97 | 1.55 |
|  |  | BS | -1.45 | 2.03 |
|  |  | SD | -1.50 | 2.13 |
|  |  | Feed1 | -2.50 | 0.20 |
|  |  | Feed2 | **0.39** | **2.92** |
|  |  | Feed3 | -1.65 | 0.69 |
|  | *Sorex araneus* | Ab | -2.49 | 0.88 |
|  |  | HN | **-7.82** | **-4.13** |
|  |  | PD | **1.73** | **5.90** |
|  |  | BS | -2.74 | 1.37 |
|  |  | SD | -2.51 | 0.75 |
|  |  | Feed1 | -1.53 | 1.03 |
|  |  | Feed2 | -1.50 | 0.79 |
|  |  | Feed3 | -0.57 | 1.81 |

**Supplementary Table S22**. Estimated parameters of posterior distributions of logistic regression coefficients (percentiles 2.5% and 97.5%) for the relationships between the dark diversity affinity of regions (*dda_region_)* and their environmental variables from the species-region unified models for flea and mite component metacommunities (i.e., within a host species across regions). Environmental variables are as follows. Alt: mean altitude; T: air temperature (the first principal component; see text for explanation); P: precipitation (the first principal component; see text for explanation); NDVI: normalized difference vegetation index (the first principal component; see text for explanation); Area: area of a region; HSR: number of available host species. Bold font: significantly positive or significantly negative coefficients.

| Parasite | Host species | Environmental variable | Percentile | |
| --- | --- | --- | --- | --- |
|  |  |  | 2.5% | 95.% |
| Fleas | *Apodemus agrarius* | Alt | -4.98 | 0.41 |
|  |  | T | -0.29 | 5.07 |
|  |  | P | -3.39 | 1.17 |
|  |  | NDVI | **1.85** | **8.78** |
|  |  | Area | -3.94 | 0.53 |
|  |  | HSR | -0.33 | 4.30 |
|  | *Apodemus uralensis* | Alt | **-5.05** | **-1.14** |
|  |  | T | **0.73** | **4.72** |
|  |  | P | **-4.92** | **-0.82** |
|  |  | NDVI | -3.66 | 0.53 |
|  |  | Area | -1.97 | 1.18 |
|  |  | HSR | -2.06 | 1.26 |
|  | *Cricetulus migratorius* | Alt | **-11.79** | **-3.96** |
|  |  | T | **-8.30** | **-1.10** |
|  |  | P | -1.37 | 4.48 |
|  |  | NDVI | -4.81 | 0.56 |
|  |  | Area | -4.62 | 1.37 |
|  |  | HSR | **-6.62** | **-1.26** |
|  | *Microtus arvalis* | Alt | -2.79 | 0.78 |
|  |  | T | **-6.19** | **-0.83** |
|  |  | P | **-6.75** | **-0.14** |
|  |  | NDVI | **-8.31** | **-2.41** |
|  |  | Area | -1.24 | 2.18 |
|  |  | HSR | **-4.31** | **-0.06** |
|  | *Microtus oeconomus* | Alt | -1.60 | 3.32 |
|  |  | T | -0.46 | 4.91 |
|  |  | P | -2.81 | 1.56 |
|  |  | NDVI | -0.51 | 5.18 |
|  |  | Area | -4.78 | 0.71 |
|  |  | HSR | -4.70 | 2.06 |
|  | *Myodes rutilus* | Alt | **-4.41** | **-0.39** |
|  |  | T | -0.35 | 3.19 |
|  |  | P | **0.35** | **4.05** |
|  |  | NDVI | **0.64** | **3.64** |
|  |  | Area | -0.93 | 3.69 |
|  |  | HSR | -4.04 | 0.45 |
|  | *Sorex araneus* | Alt | -0.75 | 1.85 |
|  |  | T | -1.61 | 2.54 |
|  |  | P | -3.39 | 0.34 |
|  |  | NDVI | -1.29 | 2.66 |
|  |  | Area | -2.16 | 1.39 |
|  |  | HSR | **-5.55** | **-1.71** |
| Mites | *Craseomys rufocanus* | Alt | -3.76 | 1.40 |
|  |  | T | -0.51 | 7.79 |
|  |  | P | -2.30 | 3.34 |
|  |  | NDVI | -7.09 | 0.00 |
|  |  | Area | **-5.25** | **-0.11** |
|  |  | HSR | **-6.76** | **-0.83** |
|  | *Microtus oeconomus* | Alt | -2.82 | 1.06 |
|  |  | T | -2.00 | 4.08 |
|  |  | P | -0.55 | 3.53 |
|  |  | NDVI | -0.71 | 4.32 |
|  |  | Area | -0.86 | 3.08 |
|  |  | HSR | **-4.51** | **-0.30** |
|  | *Myodes glareolus* | Alt | -4.55 | 2.98 |
|  |  | T | -3.95 | 1.85 |
|  |  | P | -9.66 | 0.37 |
|  |  | NDVI | -2.62 | 3.72 |
|  |  | Area | -0.37 | 4.27 |
|  |  | HSR | -2.72 | 2.49 |
|  | *Myodes rutilus* | Alt | -1.31 | 1.97 |
|  |  | T | -2.88 | 2.32 |
|  |  | P | -0.40 | 3.18 |
|  |  | NDVI | -0.65 | 3.63 |
|  |  | Area | -2.94 | 0.97 |
|  |  | HSR | **-5.53** | **-1.24** |
|  | *Sorex araneus* | Alt | **-4.85** | **-0.36** |
|  |  | T | -3.07 | 2.44 |
|  |  | P | **-10.31** | **-1.23** |
|  |  | NDVI | **0.78** | **6.88** |
|  |  | Area | -1.37 | 1.91 |
|  |  | HSR | -3.75 | 0.08 |

**Supplementary Table S23**. Estimated parameters of posterior distributions of logistic regression coefficients (percentiles 2.5% and 97.5%) for the relationships between the dark diversity affinity of parasite species (*dda_sp_*) and their traits from the species-host unified models for flea and mite compound metacommunities. Multiple class-specific intercepts were estimated for parameters of categorical variables with more than one density distribution. Traits are as follows. Ab: characteristic abundance; HN: number of host species across geographic range; PD: phylogenetic diversity of these hosts; BS: body size; SD: sexual size dimorphism; MHpref: microhabitat preference (for fleas) (1: host’s hair; 2: no clear preference; 3: host’s nest); Combs: possession and number of sclerotized combs (for fleas) (1: no combs, 2: one comb; 3: two combs); Feed: feeding mode (for mites) (1: facultative haematophage, 2: obligatory exclusive haematophage, 3 – obligatory non-exclusive haematophage). Bold font: significantly positive or significantly negative coefficients.

| Parasite | Region | Trait | Percentile | |
| --- | --- | --- | --- | --- |
|  |  |  | 2.5% | 97.% |
| Fleas | Altai Mountains | Ab | -39.58 | 3.16 |
|  |  | HN | -4.95 | 6.81 |
|  |  | PD | -10.01 | 6.32 |
|  |  | BS | -7.70 | 5.75 |
|  |  | SD | -2.47 | 36.29 |
|  |  | MHpref1 | -2.58 | 5.77 |
|  |  | MHpref2 | -5.06 | 6.16 |
|  |  | MHpref3 | -9.82 | 5.08 |
|  |  | Combs1 | - | - |
|  |  | Combs2 | -2.11 | 9.32 |
|  |  | Combs3 | -9.32 | 2.11 |
|  | Armenia | Ab | -3.96 | 0.15 |
|  |  | HN | -2.43 | 1.17 |
|  |  | PD | -3.08 | 0.85 |
|  |  | BS | -0.11 | 4.94 |
|  |  | SD | -2.30 | 1.61 |
|  |  | MHpref1 | -1.16 | 2.25 |
|  |  | MHpref2 | -0.68 | 3.35 |
|  |  | MHpref3 | -4.22 | 0.35 |
|  |  | Combs1 | -0.89 | 4.68 |
|  |  | Combs2 | **-4.63** | **-0.41** |
|  |  | Combs3 | -1.40 | 2.78 |
|  | Dzungarian Alatau | Ab | **-15.07** | **-3.66** |
|  |  | HN | -5.43 | 1.39 |
|  |  | PD | **-11.72** | **-1.68** |
|  |  | BS | -9.08 | -0.68 |
|  |  | SD | -0.26 | 5.08 |
|  |  | MHpref1 | -1.74 | 1.53 |
|  |  | MHpref2 | -1.85 | 3.17 |
|  |  | MHpref3 | -3.14 | 2.06 |
|  |  | Combs1 | - | - |
|  |  | Combs2 | -2.55 | 1.09 |
|  |  | Combs3 | -1.09 | 2.55 |
|  | Kurgan | Ab | **-19.20** | **-4.70** |
|  |  | HN | **-11.81** | **-0.40** |
|  |  | PD | -5.25 | 1.90 |
|  |  | BS | -14.38 | 2.37 |
|  |  | SD | -4.66 | 3.67 |
|  |  | MHpref1 | **-5.47** | **-0.13** |
|  |  | MHpref2 | -2.69 | 6.55 |
|  |  | MHpref3 | -2.71 | 4.67 |
|  |  | Combs1 | - | - |
|  |  | Combs2 | **-9.09** | **-1.28** |
|  |  | Combs3 | 1.28 | 9.09 |
|  | Poland | Ab | -3.29 | 0.95 |
|  |  | HN | **-6.89** | **-2.14** |
|  |  | PD | -1.18 | 2.09 |
|  |  | BS | -0.65 | 2.26 |
|  |  | SD | -0.79 | 2.21 |
|  |  | MHpref1 | -1.93 | 0.77 |
|  |  | MHpref2 | -0.81 | 3.38 |
|  |  | MHpref3 | -2.32 | 0.80 |
|  |  | Combs1 | - | - |
|  |  | Combs2 | -0.81 | 1.49 |
|  |  | Combs3 | -1.49 | 0.81 |
|  | Tomsk-Tyumen | Ab | **-10.51** | **-2.75** |
|  |  | HN | -3.29 | 1.11 |
|  |  | PD | **-7.33** | **-1.54** |
|  |  | BS | **-4.75** | **-0.33** |
|  |  | SD | -1.46 | 3.36 |
|  |  | MHpref1 | **-4.35** | **-1.28** |
|  |  | MHpref2 | -3.49 | 0.84 |
|  |  | MHpref3 | **1.49** | **7.00** |
|  |  | Combs1 | - | - |
|  |  | Combs2 | **1.16** | **4.67** |
|  |  | Combs3 | **-4.67** | **-1.16** |
|  | Turkmenistan | Ab | -4.40 | -0.19 |
|  |  | HN | -4.36 | 0.47 |
|  |  | PD | -0.53 | 3.51 |
|  |  | BS | -2.93 | 1.09 |
|  |  | SD | -0.33 | 3.50 |
|  |  | MHpref1 | -2.85 | 0.55 |
|  |  | MHpref2 | -2.05 | 1.52 |
|  |  | MHpref3 | -0.68 | 3.33 |
|  |  | Combs1 | -3.17 | 0.06 |
|  |  | Combs2 | 0.58 | 4.24 |
|  |  | Combs3 | -2.66 | 0.96 |
|  | Tatarstan | Ab | -2.15 | 1.34 |
|  |  | HN | **-9.54** | **-4.92** |
|  |  | PD | **-3.58** | **-0.21** |
|  |  | BS | -1.28 | 2.04 |
|  |  | SD | -1.78 | 1.37 |
|  |  | MHpref1 | -1.14 | 1.11 |
|  |  | MHpref2 | -0.81 | 2.60 |
|  |  | MHpref3 | -2.46 | 0.61 |
|  |  | Combs1 | - | - |
|  |  | Combs2 | -0.98 | 1.02 |
|  |  | Combs3 | -1.02 | 0.98 |
| Mites | Chulym River | Ab | -6.94 | 3.13 |
|  |  | HN | **-22.80** | **-0.55** |
|  |  | PD | -3.64 | 5.05 |
|  |  | BS | **-20.49** | **-0.44** |
|  |  | SD | -10.72 | 2.19 |
|  |  | Feed1 | -0.50 | 13.63 |
|  |  | Feed2 | -10.82 | 1.57 |
|  |  | Feed3 | -6.20 | 2.39 |
|  | Krasnodar | Ab | **-24.00** | -1.43 |
|  |  | HN | -4.95 | 3.19 |
|  |  | PD | -0.77 | 7.68 |
|  |  | BS | -7.91 | 1.21 |
|  |  | SD | -17.03 | 1.12 |
|  |  | Feed1 | **-16.05** | **-1.38** |
|  |  | Feed2 | -2.41 | 5.49 |
|  |  | Feed3 | 1.70 | 12.26 |
|  | Northern Russian Far East | Ab | -9.99 | 1.83 |
|  |  | HN | **-14.86** | **-3.32** |
|  |  | PD | -1.82 | 6.86 |
|  |  | BS | -4.36 | 2.86 |
|  |  | SD | -3.39 | 4.92 |
|  |  | Feed1 | -1.75 | 6.09 |
|  |  | Feed2 | -6.52 | 0.03 |
|  |  | Feed3 | -1.76 | 3.13 |
|  | Novosibirsk | Ab | **-5.33** | **-0.81** |
|  |  | HN | **-16.88** | **-9.30** |
|  |  | PD | -3.34 | 1.47 |
|  |  | BS | **-7.11** | **-1.15** |
|  |  | SD | -6.63 | 0.30 |
|  |  | Feed1 | 1.78 | 6.13 |
|  |  | Feed2 | **-4.20** | **-0.80** |
|  |  | Feed3 | -3.25 | 0.04 |
|  | Omsk Forest-Steppe Zone | Ab | **-5.36** | **-1.08** |
|  |  | HN | **-13.64** | **-6.59** |
|  |  | PD | -0.93 | 4.43 |
|  |  | BS | 0.32 | 6.94 |
|  |  | SD | -4.74 | 0.29 |
|  |  | Feed1 | -1.17 | 2.89 |
|  |  | Feed2 | -1.38 | 1.81 |
|  |  | Feed3 | -2.16 | 0.19 |
|  | Southern Russian Far East | Ab | -3.05 | 0.97 |
|  |  | HN | **-7.95** | **-3.07** |
|  |  | PD | -2.48 | 1.31 |
|  |  | BS | -2.80 | 1.29 |
|  |  | SD | 0.14 | 3.44 |
|  |  | Feed1 | -1.96 | 0.82 |
|  |  | Feed2 | -2.64 | 0.05 |
|  |  | Feed3 | - | - |

**Supplementary Table S24**. Estimated parameters of posterior distributions of logistic regression coefficients (percentiles 2.5% and 97.5%) for the relationships between the dark diversity affinity of host species (*dda_host_*) and their traits from the species-host unified models of flea compound metacommunities in the Altai Mountains and Armenia. Host traits are as follows. BS: body size, BR: relative brain size; DISP: dispersal range; GR: geographic range size; HB: habitat breadth (categorical, 1 to 6); NEST: location of nest (categorical, 1: above ground, 2: on ground, 3: below ground, 4: below or on ground); LF: life form (categorical, 1: ground-dwelling, 2: arboreal, 3: fossorial, 4: aquatic); DIET: feeding habits (categorical, 1: granivorous, 2: omnivorous, 3: folivorous, 4: insectivorous, 5: grani-folivorous, 6: grani-insectivorous); ACT: diel activity (categorical, 1: nocturnal, 2: cathemeral, 3: nocturnal); HYB: hibernation (categorical, 1: yes, 2: no); SOC: sociality (categorical, 1: solitary, 2: social); SDEP: shelter depth (categorical, 1: shallow, 2: intermediate, 3: deep), SCOMP: shelter complexity (categorical, 1: simple, 2: intermediate, 3: complex). Bold font: significantly positive or significantly negative coefficients.

| Region | Host trait | Percentile | |
| --- | --- | --- | --- |
|  |  | 2.5% | 95.% |
| Altai Mountains | BS | -7.29 | 8.74 |
|  | BR | -5.48 | 16.10 |
|  | DISP | **1.07** | **30.19** |
|  | GR | -6.46 | 8.88 |
|  | HB1 | - | - |
|  | HB2 | -6.68 | 6.89 |
|  | HB3 | -1.75 | 9.80 |
|  | HB4 | -30.30 | 1.05 |
|  | HB5 | -4.26 | 14.66 |
|  | HB6 | -4.06 | 9.36 |
|  | NEST1 | - | - |
|  | NEST2 | -0.89 | 23.13 |
|  | NEST3 | -11.18 | 2.05 |
|  | NEST4 | -13.25 | 1.48 |
|  | LF1 | -5.43 | 10.06 |
|  | LF2 | - | - |
|  | LF3 | - | - |
|  | LF4 | -10.06 | 5.43 |
|  | DIET1 | -11.12 | 13.27 |
|  | DIET2 | -15.77 | 13.35 |
|  | DIET3 | -13.35 | 8.82 |
|  | DIET4 | -9.22 | 14.47 |
|  | DIET5 | - | - |
|  | DIET6 | -13.55 | 9.96 |
|  | ACT1 | -24.83 | 4.52 |
|  | ACT2 | -3.24 | 16.46 |
|  | ACT3 | -6.34 | 12.31 |
|  | HYB1 | -23.81 | 4.67 |
|  | HYB2 | -4.67 | 23.81 |
|  | SOC1 | -13.51 | 3.91 |
|  | SOC2 | -3.91 | 13.51 |
|  | SDEP1 | -8.75 | 12.90 |
|  | SDEP2 | -11.29 | 5.65 |
|  | SDEP3 | -6.93 | 9.93 |
|  | SCOMP1 |  |  |
|  | SCOPM2 | -19.37 | 2.75 |
|  | SCOMP3 | -2.75 | 19.37 |
| Armenia | BS | -7.95 | 4.91 |
|  | BR | -12.87 | 3.95 |
|  | DISP | -7.91 | 4.18 |
|  | GR | -9.19 | 2.23 |
|  | HB1 | -1.01 | 8.70 |
|  | HB2 | -2.05 | 9.66 |
|  | HB3 | **-28.43** | **-0.31** |
|  | HB4 | -8.72 | 6.77 |
|  | HB5 | -2.64 | 11.33 |
|  | HB6 | - | - |
|  | NEST1 | -6.08 | 11.82 |
|  | NEST2 | -11.51 | 1.23 |
|  | NEST3 | -3.67 | 9.37 |
|  | NEST4 | - | - |
|  | LF1 | -2.83 | 6.51 |
|  | LF2 | - | - |
|  | LF3 | -5.16 | 10.98 |
|  | LF4 | -10.76 | 2.61 |
|  | DIET1 | -1.43 | 13.99 |
|  | DIET2 | -10.39 | 2.51 |
|  | DIET3 | -13.06 | 2.18 |
|  | DIET4 | -2.95 | 9.83 |
|  | DIET5 | -7.67 | 5.37 |
|  | DIET6 | - | - |
|  | ACT1 | -7.77 | 2.91 |
|  | ACT2 | -1.33 | 10.68 |
|  | ACT3 | -6.94 | 3.00 |
|  | HYB1 | -9.45 | 1.70 |
|  | HYB2 | -1.70 | 9.45 |
|  | SOC1 | -2.32 | 4.68 |
|  | SOC2 | -4.68 | 2.32 |
|  | SDEP1 | -4.00 | 10.61 |
|  | SDEP2 | -4.78 | 4.63 |
|  | SDEP3 | -7.89 | 2.95 |
|  | SCOMP1 | -4.15 | 10.91 |
|  | SCOPM2 | -7.21 | 2.11 |
|  | SCOMP3 | -4.70 | 4.04 |

**Supplementary Table S25**. Estimated parameters of posterior distributions of logistic regression coefficients (percentiles 2.5% and 97.5%) for the relationships between the dark diversity affinity of host species (*dda_host_*) and their traits from the species-host unified models for flea compound metacommunities in Dzungarian Alatau and Kurgan (Southern Ural Mountains). Host traits are as follows. BS: body size, BR: relative brain size; DISP: dispersal range; GR: geographic range size; HB: habitat breadth (categorical, 1 to 6); NEST: location of nest (categorical, 1: above ground, 2: on ground, 3: below ground, 4: below or on ground); LF: life form (categorical, 1: ground-dwelling, 2: arboreal, 3: fossorial, 4: aquatic); DIET: feeding habits (categorical, 1: granivorous, 2: omnivorous, 3: folivorous, 4: insectivorous, 5: grani-folivorous, 6: grani-insectivorous); ACT: diel activity (categorical, 1: nocturnal, 2: cathemeral, 3: nocturnal); HYB: hibernation (categorical, 1: yes, 2: no); SOC: sociality (categorical, 1: solitary, 2: social); SDEP: shelter depth (categorical, 1: shallow, 2: intermediate, 3: deep), SCOMP: shelter complexity (categorical, 1: simple, 2: intermediate, 3: complex). Bold font: significantly positive or significantly negative coefficients.

| Region | Host trait | Percentile | |
| --- | --- | --- | --- |
|  |  | 2.5% | 95.% |
| Dzungarian Alatau | BS | -3.47 | 14.13 |
|  | BR | -4.65 | 5.23 |
|  | DISP | -6.59 | 6.50 |
|  | GR | -2.74 | 14.05 |
|  | HB1 | -1.41 | 9.62 |
|  | HB2 | -8.37 | 0.68 |
|  | HB3 | -3.62 | 4.88 |
|  | HB4 | -7.72 | 3.97 |
|  | HB5 | -4.61 | 8.28 |
|  | HB6 |  |  |
|  | NEST1 | -6.88 | 7.71 |
|  | NEST2 | -2.60 | 9.59 |
|  | NEST3 | -4.38 | 5.39 |
|  | NEST4 | -12.90 | 2.42 |
|  | LF1 | -2.87 | 4.10 |
|  | LF2 |  |  |
|  | LF3 | -5.73 | 4.99 |
|  | LF4 |  |  |
|  | DIET1 | -5.46 | 4.80 |
|  | DIET2 | -3.03 | 6.97 |
|  | DIET3 | -5.73 | 7.21 |
|  | DIET4 | -11.19 | 1.46 |
|  | DIET5 | -3.96 | 8.54 |
|  | DIET6 |  |  |
|  | ACT1 | -6.30 | 2.52 |
|  | ACT2 | -2.81 | 8.78 |
|  | ACT3 | -5.39 | 4.00 |
|  | HYB1 | -6.20 | 2.80 |
|  | HYB2 | -2.80 | 6.20 |
|  | SOC1 | -2.49 | 3.75 |
|  | SOC2 | -3.75 | 2.49 |
|  | SDEP1 | -8.37 | 5.82 |
|  | SDEP2 | -13.84 | 2.16 |
|  | SDEP3 | -5.23 | 6.79 |
|  | SCOMP1 | -5.36 | 6.63 |
|  | SCOPM2 | -4.46 | 5.52 |
|  | SCOMP3 | -7.19 | 5.07 |
| Kurgan | BS | -8.24 | 6.25 |
|  | BR | -11.93 | 4.53 |
|  | DISP | -3.78 | 3.65 |
|  | GR | -1.58 | 15.16 |
|  | HB1 | **-32.20** | **-0.59** |
|  | HB2 | **-** | **-** |
|  | HB3 | -1.44 | 7.18 |
|  | HB4 | -3.76 | 9.13 |
|  | HB5 | -2.72 | 13.12 |
|  | HB6 | -2.37 | 7.86 |
|  | NEST1 | -7.29 | 9.83 |
|  | NEST2 | -4.74 | 5.92 |
|  | NEST3 | -3.43 | 6.86 |
|  | NEST4 | -9.40 | 2.16 |
|  | LF1 | -5.53 | 3.43 |
|  | LF2 | -4.48 | 7.02 |
|  | LF3 | - | - |
|  | LF4 | -4.74 | 4.76 |
|  | DIET1 | -3.42 | 8.72 |
|  | DIET2 | -9.25 | 8.98 |
|  | DIET3 | -13.62 | 4.37 |
|  | DIET4 | -6.23 | 7.26 |
|  | DIET5 | - | - |
|  | DIET6 | -9.54 | 9.53 |
|  | ACT1 | -6.48 | 3.79 |
|  | ACT2 | -2.58 | 6.44 |
|  | ACT3 | -5.57 | 3.86 |
|  | HYB1 | -9.59 | 3.41 |
|  | HYB2 | -3.41 | 9.59 |
|  | SOC1 | -5.57 | 4.34 |
|  | SOC2 | -4.34 | 5.57 |
|  | SDEP1 | -5.29 | 6.33 |
|  | SDEP2 | -6.33 | 5.29 |
|  | SDEP3 | - | - |
|  | SCOMP1 | -4.85 | 12.78 |
|  | SCOPM2 | -6.36 | 4.78 |
|  | SCOMP3 | -10.02 | 4.88 |

**Supplementary Table S26**. Estimated parameters of posterior distributions of logistic regression coefficients (percentiles 2.5% and 97.5%) for the relationships between dark diversity affinity of host species (*dda_host_*) and their traits from the species-host unified models for flea compound metacommunities in Poland and Tomsk-Tyumen (Southeastern Siberia). Host traits are as follows. BS: body size, BR: relative brain size; DISP: dispersal range; GR: geographic range size; HB: habitat breadth (categorical, 1 to 6); NEST: location of nest (categorical, 1 – above ground, 2 – on ground, 3 – below ground, 4 – below or on ground); LF: life form (categorical, 1 – ground-dwelling, 2 – arboreal, 3 – fossorial, 4 – aquatic); DIET: feeding habits (categorical, 1 – granivorous, 2 – omnivorous, 3 – folivorous, 4 – insectivorous, 5 - grani-folivorous, 6 – grani-insectivorous); ACT: diel activity (categorical, 1 – nocturnal, 2 – cathemeral, 3 – nocturnal); HYB: hibernation (categorical, 1 – yes, 2 – no); SOC: sociality (categorical, 1 – solitary, 2 – social); SDEP: shelter depth (categorical, 1 – shallow, 2 – intermediate, 3 – deep), SCOMP: shelter complexity (categorical, 1 – simple, 2 – intermediate, 3 – complex). Bold font: significantly positive or significantly negative coefficients.

| Region | Host trait | Percentile | |
| --- | --- | --- | --- |
|  |  | 2.5% | 95.% |
| Poland | BS | -22.56 | 0.50 |
|  | BR | -3.30 | 5.90 |
|  | DISP | -4.17 | 7.24 |
|  | GR | -3.79 | 3.53 |
|  | HB1 | -1.60 | 6.55 |
|  | HB2 | -8.46 | 1.66 |
|  | HB3 | -3.87 | 3.38 |
|  | HB4 | -5.53 | 3.76 |
|  | HB5 | -3.86 | 3.96 |
|  | HB6 | -2.48 | 5.46 |
|  | NEST1 | -6.22 | 9.66 |
|  | NEST2 | -6.16 | 4.79 |
|  | NEST3 | -4.50 | 4.83 |
|  | NEST4 | -6.69 | 3.89 |
|  | LF1 | -4.52 | 3.80 |
|  | LF2 | -3.33 | 8.27 |
|  | LF3 | -8.30 | 4.38 |
|  | LF4 | -6.44 | 5.40 |
|  | DIET1 | -2.20 | 6.27 |
|  | DIET2 | -5.81 | 4.24 |
|  | DIET3 | -8.82 | 3.43 |
|  | DIET4 | -4.41 | 9.03 |
|  | DIET5 | - | - |
|  | DIET6 | -10.39 | 6.50 |
|  | ACT1 | -3.02 | 5.06 |
|  | ACT2 | -6.13 | 1.55 |
|  | ACT3 | -3.78 | 6.45 |
|  | HYB1 | 0.00 | 0.00 |
|  | HYB2 | -5.82 | 4.92 |
|  | SOC1 | -4.92 | 5.82 |
|  | SOC2 | -5.43 | 8.83 |
|  | SDEP1 | -4.37 | 6.54 |
|  | SDEP2 | -10.64 | 4.35 |
|  | SDEP3 | - | - |
|  | SCOMP1 | -5.57 | 10.10 |
|  | SCOPM2 | -8.39 | 3.99 |
|  | SCOMP3 | -7.12 | 7.63 |
| Tomsk-Tyumen | BS | -2.64 | 7.22 |
|  | BR | -3.61 | 5.88 |
|  | DISP | -1.82 | 5.24 |
|  | GR | -5.60 | 0.78 |
|  | HB1 | -2.35 | 3.27 |
|  | HB2 | -4.54 | 0.39 |
|  | HB3 | -4.39 | 1.59 |
|  | HB4 | -8.21 | 3.53 |
|  | HB5 | -1.39 | 4.95 |
|  | HB6 | -0.86 | 6.11 |
|  | NEST1 | -9.08 | 6.41 |
|  | NEST2 | -4.01 | 5.66 |
|  | NEST3 | -4.13 | 2.86 |
|  | NEST4 | -2.83 | 4.45 |
|  | LF1 | -5.04 | 0.53 |
|  | LF2 | - | - |
|  | LF3 | - | - |
|  | LF4 | -0.53 | 5.03 |
|  | DIET1 | -7.26 | 2.47 |
|  | DIET2 | -2.35 | 12.60 |
|  | DIET3 | -5.00 | 1.85 |
|  | DIET4 | -4.62 | 6.85 |
|  | DIET5 | - | - |
|  | DIET6 | -4.67 | 2.99 |
|  | ACT1 | -4.03 | 2.35 |
|  | ACT2 | -2.05 | 4.35 |
|  | ACT3 | -3.86 | 3.37 |
|  | HYB1 | -4.85 | 1.75 |
|  | HYB2 | -1.75 | 4.85 |
|  | SOC1 | -1.53 | 3.35 |
|  | SOC2 | -3.35 | 1.53 |
|  | SDEP1 | -2.77 | 6.68 |
|  | SDEP2 | -2.24 | 3.88 |
|  | SDEP3 | -6.06 | 0.86 |
|  | SCOMP1 | -5.02 | 7.04 |
|  | SCOPM2 | -4.00 | 3.74 |
|  | SCOMP3 | -5.78 | 4.24 |

**Supplementary Table S27**. Estimated parameters of posterior distributions of logistic regression coefficients (percentiles 2.5% and 97.5%) for the relationships between the dark diversity affinity of host species (*dda_host_*) and their traits from the species-host unified models for flea compound metacommunities in Turkmenistan and Tatarstan. Host traits are as follows. BS: body size, BR: relative brain size; DISP: dispersal range; GR: geographic range size; HB: habitat breadth (categorical, 1 to 6); NEST: location of nest (categorical, 1: above ground, 2: on ground, 3: below ground, 4: below or on ground); LF: life form (categorical, 1: ground-dwelling, 2: arboreal, 3: fossorial, 4: aquatic); DIET: feeding habits (categorical, 1: granivorous, 2: omnivorous, 3: folivorous, 4: insectivorous, 5: grani-folivorous, 6: grani-insectivorous); ACT: diel activity (categorical, 1: nocturnal, 2: cathemeral, 3: nocturnal); HYB: hibernation (categorical, 1: yes, 2: no); SOC: sociality (categorical, 1: solitary, 2: social); SDEP: shelter depth (categorical, 1: shallow, 2: intermediate, 3: deep), SCOMP: shelter complexity (categorical, 1: simple, 2: intermediate, 3: complex). Bold font: significantly positive or significantly negative coefficients.

| Region | Host trait | Percentile | |
| --- | --- | --- | --- |
|  |  | 2.5% | 95.% |
| Turkmenistan | BS | -4.99 | 6.35 |
|  | BR | -3.97 | 6.89 |
|  | DISP | -7.20 | 4.19 |
|  | GR | -8.11 | 2.38 |
|  | HB1 | -0.69 | 13.33 |
|  | HB2 | -6.42 | 3.38 |
|  | HB3 | -5.92 | 7.19 |
|  | HB4 | -22.46 | 3.19 |
|  | HB5 | -4.18 | 9.72 |
|  | HB6 | - | - |
|  | NEST1 | - | - |
|  | NEST2 | -7.84 | 5.50 |
|  | NEST3 | -7.22 | 3.73 |
|  | NEST4 | -4.34 | 11.23 |
|  | LF1 | -10.15 | 1.93 |
|  | LF2 | -4.62 | 9.50 |
|  | LF3 | -4.90 | 7.99 |
|  | LF4 | - | - |
|  | DIET1 | -4.54 | 5.19 |
|  | DIET2 | -2.82 | 5.48 |
|  | DIET3 | -1.81 | 5.12 |
|  | DIET4 | -4.18 | 4.42 |
|  | DIET5 | -11.92 | 2.27 |
|  | DIET6 | - | - |
|  | ACT1 | -2.39 | 6.42 |
|  | ACT2 | -5.22 | 4.46 |
|  | ACT3 | -7.37 | 3.44 |
|  | HYB1 | -5.11 | 1.69 |
|  | HYB2 | -1.69 | 5.11 |
|  | SOC1 | -4.35 | 3.96 |
|  | SOC2 | -1.96 | 4.42 |
|  | SDEP1 | -4.46 | 6.09 |
|  | SDEP2 | -5.19 | 5.20 |
|  | SDEP3 | -7.61 | 5.05 |
|  | SCOMP1 | -4.79 | 3.07 |
|  | SCOPM2 | -4.96 | 3.56 |
|  | SCOMP3 | -3.70 | 7.65 |
| Tatarstan | BS | -10.78 | 6.50 |
|  | BR | -6.55 | 4.66 |
|  | DISP | -3.25 | 8.86 |
|  | GR | -1.14 | 20.46 |
|  | HB1 | -10.48 | 8.26 |
|  | HB2 | -7.11 | 1.23 |
|  | HB3 | -6.76 | 3.53 |
|  | HB4 | -3.95 | 4.51 |
|  | HB5 | -2.40 | 9.01 |
|  | HB6 | -2.09 | 6.41 |
|  | NEST1 | -4.72 | 15.73 |
|  | NEST2 | -7.33 | 2.77 |
|  | NEST3 | -4.82 | 4.16 |
|  | NEST4 | -6.77 | 2.55 |
|  | LF1 | -4.31 | 4.75 |
|  | LF2 | -9.29 | 4.03 |
|  | LF3 | -8.48 | 5.82 |
|  | LF4 | -1.62 | 8.90 |
|  | DIET1 | -5.05 | 3.81 |
|  | DIET2 | -6.31 | 10.81 |
|  | DIET3 | -5.66 | 4.15 |
|  | DIET4 | -7.48 | 2.99 |
|  | DIET5 | - | - |
|  | DIET6 | -2.73 | 7.99 |
|  | ACT1 | -3.11 | 4.77 |
|  | ACT2 | -3.94 | 3.46 |
|  | ACT3 | -4.70 | 3.19 |
|  | HYB1 | -8.37 | 3.40 |
|  | HYB2 | -3.40 | 8.37 |
|  | SOC1 | -2.13 | 4.10 |
|  | SOC2 | -4.10 | 2.13 |
|  | SDEP1 | -4.53 | 11.21 |
|  | SDEP2 | -2.66 | 7.22 |
|  | SDEP3 | -11.08 | 0.18 |
|  | SCOMP1 | -4.88 | 17.56 |
|  | SCOPM2 | -6.91 | 4.49 |
|  | SCOMP3 | -8.89 | 4.03 |

**Supplementary Table S28**. Estimated parameters of posterior distributions of logistic regression coefficients (percentiles 2.5% and 97.5%) for the relationships between the dark diversity affinity of host species (*dda_host_*) and their traits from the species-host unified models for mite compound metacommunities in the Chulym River (Khakassia) and Krasnodar (Southwestern Russia). Host traits are as follows. BS: body size, BR: relative brain size; DISP: dispersal range; GR: geographic range size; HB: habitat breadth (categorical, 1 to 6); NEST: location of nest (categorical, 1: above ground, 2: on ground, 3: below ground, 4: below or on ground); LF: life form (categorical, 1: ground-dwelling, 2: arboreal, 3: fossorial, 4: aquatic); DIET: feeding habits (categorical, 1: granivorous, 2: omnivorous, 3: folivorous, 4: insectivorous, 5: grani-folivorous, 6: grani-insectivorous); ACT: diel activity (categorical, 1: nocturnal, 2: cathemeral, 3: nocturnal); HYB: hibernation (categorical, 1: yes, 2: no); SOC: sociality (categorical, 1: solitary, 2: social); SDEP: shelter depth (categorical, 1: shallow, 2: intermediate, 3: deep), SCOMP: shelter complexity (categorical, 1: simple, 2: intermediate, 3: complex). Bold font: significantly positive or significantly negative coefficients.

| Region | Host trait | Percentile | |
| --- | --- | --- | --- |
|  |  | 2.5% | 97.5% |
| Chulym River | BS | -7.05 | 9.31 |
|  | BR | -7.79 | 9.28 |
|  | DISP | -9.39 | 10.34 |
|  | GR | -10.63 | 4.80 |
|  | HB1 | -10.76 | 12.93 |
|  | HB2 | -8.28 | 6.05 |
|  | HB3 | -2.34 | 16.63 |
|  | HB4 | -19.25 | 2.88 |
|  | HB5 | -8.13 | 5.69 |
|  | HB6 | -8.65 | 12.04 |
|  | NEST1 | -8.61 | 7.14 |
|  | NEST2 | - | - |
|  | NEST3 | -2.79 | 8.91 |
|  | NEST4 | -10.05 | 4.21 |
|  | LF1 | -6.74 | 6.35 |
|  | LF2 | -13.88 | 6.54 |
|  | LF3 | -9.37 | 13.09 |
|  | LF4 | -8.27 | 9.64 |
|  | DIET1 | -12.07 | 7.56 |
|  | DIET2 | -6.72 | 9.78 |
|  | DIET3 | -13.30 | 3.82 |
|  | DIET4 | -3.60 | 12.41 |
|  | DIET5 | - | - |
|  | DIET6 | - | - |
|  | ACT1 | -8.80 | 4.78 |
|  | ACT2 | -4.69 | 6.09 |
|  | ACT3 | -4.89 | 7.03 |
|  | HYB1 | -7.82 | 3.72 |
|  | HYB2 | -3.73 | 7.82 |
|  | SOC1 | -4.70 | 6.93 |
|  | SOC2 | -6.93 | 4.70 |
|  | SDEP1 | -5.63 | 10.16 |
|  | SDEP2 | -6.09 | 6.40 |
|  | SDEP3 | -10.01 | 4.78 |
|  | SCOMP1 | -6.99 | 9.93 |
|  | SCOPM2 | -7.21 | 6.66 |
|  | SCOMP3 | -11.40 | 4.75 |
| Krasnodar | BS | -5.23 | 4.91 |
|  | BR | -5.60 | 6.38 |
|  | DISP | -13.62 | 4.12 |
|  | GR | -5.22 | 4.73 |
|  | HB1 | -4.98 | 9.52 |
|  | HB2 | -6.69 | 5.66 |
|  | HB3 | -6.09 | 9.27 |
|  | HB4 | -9.92 | 4.03 |
|  | HB5 | -5.39 | 5.79 |
|  | HB6 | -10.39 | 8.11 |
|  | NEST1 | -4.99 | 6.61 |
|  | NEST2 | - | - |
|  | NEST3 | -8.94 | 2.75 |
|  | NEST4 | -2.94 | 8.58 |
|  | LF1 | -4.80 | 5.48 |
|  | LF2 | -7.67 | 5.35 |
|  | LF3 | -10.07 | 7.75 |
|  | LF4 | -6.52 | 10.61 |
|  | DIET1 | -6.13 | 4.83 |
|  | DIET2 | -9.62 | 2.22 |
|  | DIET3 | -7.93 | 5.36 |
|  | DIET4 | -2.38 | 14.62 |
|  | DIET5 | - | - |
|  | DIET6 | - | - |
|  | ACT1 | -3.51 | 6.58 |
|  | ACT2 | -9.22 | 5.15 |
|  | ACT3 | -5.10 | 5.76 |
|  | HYB1 | -23.24 | 0.34 |
|  | HYB2 | -0.34 | 23.24 |
|  | SOC1 | -5.13 | 4.24 |
|  | SOC2 | -4.24 | 5.13 |
|  | SDEP1 | -4.70 | 12.17 |
|  | SDEP2 | -6.42 | 10.73 |
|  | SDEP3 | -16.80 | 2.08 |
|  | SCOMP1 | -7.85 | 9.61 |
|  | SCOPM2 | -3.46 | 8.80 |
|  | SCOMP3 | -11.66 | 5.44 |

**Supplementary Table S29**. Estimated parameters of posterior distributions of logistic regression coefficients (percentiles 2.5% and 97.5%) for the relationships between the dark diversity affinity of host species (*dda_host_*) and their traits from the species-host unified models of mite compound metacommunities in the Northern Russian Far East and Novosibirsk (Southwestern Siberia). Host traits are as follows. BS: body size, BR: relative brain size; DISP: dispersal range; GR: geographic range size; HB: habitat breadth (categorical, 1 to 6); NEST: location of nest (categorical, 1: above ground, 2: on ground, 3: below ground, 4: below or on ground); LF: life form (categorical, 1: ground-dwelling, 2: arboreal, 3: fossorial, 4: aquatic); DIET: feeding habits (categorical, 1: granivorous, 2: omnivorous, 3: folivorous, 4: insectivorous, 5: grani-folivorous, 6: grani-insectivorous); ACT: diel activity (categorical, 1: nocturnal, 2: cathemeral, 3: nocturnal); HYB: hibernation (categorical, 1: yes, 2: no); SOC: sociality (categorical, 1: solitary, 2: social); SDEP: shelter depth (categorical, 1: shallow, 2: intermediate, 3: deep), SCOMP: shelter complexity (categorical, 1: simple, 2: intermediate, 3: complex). Bold font: significantly positive or significantly negative coefficients.

| Region | Host trait | Percentile | |
| --- | --- | --- | --- |
|  |  | 2.5% | 97.5% |
| Northern Russian Far East | BS | -6.27 | 11.86 |
|  | BR | -3.94 | 6.19 |
|  | DISP | -2.47 | 18.72 |
|  | GR | -7.13 | 4.78 |
|  | HB1 | -4.83 | 11.07 |
|  | HB2 | -11.46 | 0.38 |
|  | HB3 | -3.45 | 5.72 |
|  | HB4 | - | - |
|  | HB5 | -4.62 | 8.69 |
|  | HB6 | - | - |
|  | NEST1 | - | - |
|  | NEST2 | - | - |
|  | NEST3 | -2.72 | 8.73 |
|  | NEST4 | -8.73 | 2.72 |
|  | LF1 | **0.00** | **0.02** |
|  | LF2 | - | - |
|  | LF3 | - | - |
|  | LF4 | - | - |
|  | DIET1 | - | - |
|  | DIET2 | -9.17 | 1.87 |
|  | DIET3 | -5.26 | 3.34 |
|  | DIET4 | -2.24 | 10.99 |
|  | DIET5 | - | - |
|  | DIET6 | - | - |
|  | ACT1 | -4.56 | 8.19 |
|  | ACT2 | -7.26 | 3.38 |
|  | ACT3 | -5.38 | 5.82 |
|  | HYB1 | -4.77 | 4.03 |
|  | HYB2 | -4.03 | 4.77 |
|  | SOC1 | -4.48 | 3.62 |
|  | SOC2 | -3.62 | 4.48 |
|  | SDEP1 | -3.93 | 6.49 |
|  | SDEP2 | -3.59 | 6.18 |
|  | SDEP3 | -8.28 | 3.00 |
|  | SCOMP1 | -2.68 | 6.57 |
|  | SCOPM2 | -2.90 | 4.99 |
|  | SCOMP3 | -9.64 | 3.00 |
| Novosibirsk | BS | -4.14 | 4.41 |
|  | BR | -5.08 | 4.77 |
|  | DISP | -2.17 | 7.35 |
|  | GR | -1.27 | 7.57 |
|  | HB1 | -0.96 | 6.29 |
|  | HB2 | -4.10 | 1.03 |
|  | HB3 | -0.74 | 3.62 |
|  | HB4 | -6.48 | 3.32 |
|  | HB5 | -3.42 | 2.80 |
|  | HB6 | -3.84 | 1.83 |
|  | NEST1 | -6.68 | 2.34 |
|  | NEST2 | - | - |
|  | NEST3 | -2.29 | 3.95 |
|  | NEST4 | -2.28 | 4.80 |
|  | LF1 | -4.11 | 1.95 |
|  | LF2 | -2.68 | 7.92 |
|  | LF3 | -6.05 | 8.17 |
|  | LF4 | -5.76 | 2.12 |
|  | DIET1 | -6.50 | 4.02 |
|  | DIET2 | -2.49 | 7.74 |
|  | DIET3 | -3.88 | 4.40 |
|  | DIET4 | -6.32 | 3.31 |
|  | DIET5 | - | - |
|  | DIET6 | - | - |
|  | ACT1 | -2.40 | 4.59 |
|  | ACT2 | -2.49 | 3.29 |
|  | ACT3 | -4.50 | 1.57 |
|  | HYB1 | -4.98 | 1.69 |
|  | HYB2 | -1.69 | 4.98 |
|  | SOC1 | -0.95 | 5.38 |
|  | SOC2 | -5.38 | 0.95 |
|  | SDEP1 | -2.23 | 4.23 |
|  | SDEP2 | -5.85 | 0.40 |
|  | SDEP3 | -7.51 | 5.11 |
|  | SCOMP1 | -3.14 | 4.18 |
|  | SCOPM2 | -6.84 | 3.90 |
|  | SCOMP3 | -5.31 | 9.31 |

**Supplementary Table S30**. Estimated parameters of posterior distributions of logistic regression coefficients (percentiles 2.5% and 97.5%) for the relationships between the dark diversity affinity of host species (*dda_host_*) and their traits from the species-host unified models for mite compound metacommunities in the Omsk Forest-Steppe Zone (Southwestern Siberia) and Southern Russian Far East. Host traits are as follows. BS: body size, BR: relative brain size; DISP: dispersal range; GR: geographic range size; HB: habitat breadth (categorical, 1 to 6); NEST: location of nest (categorical, 1: above ground, 2: on ground, 3: below ground, 4: below or on ground); LF: life form (categorical, 1: ground-dwelling, 2: arboreal, 3: fossorial, 4: aquatic); DIET: feeding habits (categorical, 1: granivorous, 2: omnivorous, 3: folivorous, 4: insectivorous, 5: grani-folivorous, 6: grani-insectivorous); ACT: diel activity (categorical, 1: nocturnal, 2: cathemeral, 3: nocturnal); HYB: hibernation (categorical, 1: yes, 2: no); SOC: sociality (categorical, 1: solitary, 2: social); SDEP: shelter depth (categorical, 1: shallow, 2: intermediate, 3: deep), SCOMP: shelter complexity (categorical, 1: simple, 2: intermediate, 3: complex). Bold font: significantly positive or significantly negative coefficients.

| Region | Host trait | Percentile | |
| --- | --- | --- | --- |
|  |  | 2.5% | 97.5% |
| Omsk Forest-Steppe Zone | BS | -4.18 | 6.86 |
|  | BR | -3.30 | 6.23 |
|  | DISP | -0.98 | 4.84 |
|  | GR | -2.50 | 4.58 |
|  | HB1 | - | - |
|  | HB2 | -5.21 | 1.10 |
|  | HB3 | -4.60 | 0.64 |
|  | HB4 | -0.26 | 15.46 |
|  | HB5 | -6.88 | 1.24 |
|  | HB6 | -2.59 | 3.78 |
|  | NEST1 | -5.88 | 2.81 |
|  | NEST2 | - | - |
|  | NEST3 | -0.86 | 4.90 |
|  | NEST4 | -3.80 | 2.31 |
|  | LF1 | -6.03 | 0.79 |
|  | LF2 | -6.89 | 4.46 |
|  | LF3 | - | - |
|  | LF4 | -2.27 | 11.03 |
|  | DIET1 | -5.27 | 4.86 |
|  | DIET2 | -4.85 | 5.11 |
|  | DIET3 | -2.39 | 5.95 |
|  | DIET4 | -7.16 | 3.32 |
|  | DIET5 | - | - |
|  | DIET6 | - | - |
|  | ACT1 | -0.37 | 11.10 |
|  | ACT2 | -1.19 | 7.71 |
|  | ACT3 | **-16.41** | **-1.61** |
|  | HYB1 | -7.40 | 1.35 |
|  | HYB2 | -1.35 | 7.40 |
|  | SOC1 | -2.38 | 4.75 |
|  | SOC2 | -4.75 | 2.38 |
|  | SDEP1 | -3.78 | 8.34 |
|  | SDEP2 | **0.67** | **9.93** |
|  | SDEP3 | -13.45 | -1.93 |
|  | SCOMP1 | -4.59 | 6.63 |
|  | SCOPM2 | -5.16 | 3.67 |
|  | SCOMP3 | -5.89 | 4.84 |
| Southern Russian Far East | BS | -5.01 | 6.27 |
|  | BR | -4.61 | 6.18 |
|  | DISP | -6.21 | 6.04 |
|  | GR | -8.22 | 5.22 |
|  | HB1 | -4.15 | 5.45 |
|  | HB2 | **0.21** | **7.06** |
|  | HB3 | -6.16 | 5.17 |
|  | HB4 | -8.99 | 5.12 |
|  | HB5 | -10.53 | 1.75 |
|  | HB6 | -4.86 | 7.25 |
|  | NEST1 | -4.48 | 5.21 |
|  | NEST2 | - | - |
|  | NEST3 | -1.91 | 4.97 |
|  | NEST4 | -6.29 | 2.09 |
|  | LF1 | -5.77 | 0.85 |
|  | LF2 | -0.85 | 5.77 |
|  | LF3 | - | - |
|  | LF4 | - | - |
|  | DIET1 | -5.66 | 4.06 |
|  | DIET2 | -5.86 | 4.00 |
|  | DIET3 | -2.31 | 5.78 |
|  | DIET4 | -4.63 | 3.48 |
|  | DIET5 | - | - |
|  | DIET6 | - | - |
|  | ACT1 | -1.79 | 4.80 |
|  | ACT2 | -4.30 | 1.73 |
|  | ACT3 | -3.04 | 2.70 |
|  | HYB1 | -2.27 | 3.39 |
|  | HYB2 | -3.39 | 2.27 |
|  | SOC1 | -3.92 | 2.80 |
|  | SOC2 | -2.80 | 3.92 |
|  | SDEP1 | -4.27 | 4.84 |
|  | SDEP2 | -5.72 | 1.47 |
|  | SDEP3 | -1.68 | 5.09 |
|  | SCOMP1 | -4.48 | 4.97 |
|  | SCOPM2 | -4.78 | 3.01 |
|  | SCOMP3 | -3.88 | 5.38 |

**Supplementary Texts**

**Supplementary Text S1.** Ectoparasite quantitative traits: Explanations and details of some calculations.

The rationale and details of the calculations of the mean abundance and the degree of host specificity have been previously described (Poulin et al. 2011, Krasnov et al. 2013, 2015; Surkova et al. 2018). These variables were controlled for unequal sampling effort (see Krasnov et al., 2019). The phylogenetic diversity of a host spectrum was calculated as Faith’s (Faith, 1992) phylogenetic diversity using standardized values, independent of host species richness, and calculated using the “ses.pd” function of the “picante” package (Kembel et al., 2010), implemented in the R Statistical Environment (R Core Team 2024). Topologies and branch lengths of host phylogenies were taken as 1000-random trees subsets from the 10000 species-level birth-death tip-dated completed trees for the 5911 mammal species of Upham et al. (2019). Then, a consensus tree was constructed using the “consensus.edge” function of the “phytools” package (Revell, 2012), implemented in R and ultrametrized using the “force.ultrametric” function of “phytools”. The body size of a flea or a mite species was estimated via either maximal body length or the midline length of the dorsal shield, respectively, and taken as the median of the average male and average female body size (see details in Krasnov et al., 2013; Surkova et al. 2018). The degree of sexual dimorphism was calculated as the logarithmic female-to-male size ratio (Smith 1999).

**References**

Faith, D.P. (1992). Conservation evaluation and phylogenetic diversity. Biological Conservation, 61, 1–10. Doi:10.1016/0006-3207(92)91201-3

Kembel, S.W., Cowan, P.D., Helmus, M.R., Cornwell, W.K., Morlon, H., Ackerly, D.D., Blomberg, S.P., & Webb, C.O. (2010). Picante: R tools for integrating phylogenies and ecology. Bioinformatics, 26, 1463-1464. Doi:10.1093/bioinformatics/btq166

Krasnov BR, Vinarski MV, Korallo-Vinarskaya NP and Khokhlova IS (2013) Ecological correlates of body size in gamasid mites parasitic on small mammals: abundance and niche breadth. Ecography 36, 1042–1050. Doi:10.1111/j.1600-0587.2012.00140.x

Krasnov BR, Shenbrot GI, Khokhlova IS, Stanko M, Morand S, Mouillot D (2015) Assembly rules of ectoparasite communities across scales: combining patterns of abiotic factors, host composition, geographic space, phylogeny and traits. Ecography 38:184-197. Doi:10.1111/ecog.00915

Krasnov, B.R., Shenbrot, G.I., Korallo-Vinarskaya, N.P., Vinarski, M.V., Warburton, E.M. and Khokhlova, I.S. 2019. The effects of environment, hosts and space on compositional, phylogenetic and functional beta-diversity in two taxa of arthropod ectoparasites. Parasitol Res 118, 2107–2120. Doi: 10.1007/s00436-019-06371-1

Poulin R, Krasnov BR, Mouillot D (2011) Host specificity in phylogenetic and geographic space. Trends Parasitol 27:355-361. Doi:10.1016/j.pt.2011.05.003

R Core Team, 2024. R: A language and environment for statistical computing. R Foundation for Statistical Computing, Vienna. https://www.R-project.org)

Revell, L.J., 2012. phytools: An R package for phylogenetic comparative biology (and other things). Methods Ecol. Evol. 3, 217-223. Doi:10.1111/j.2041-210X.2011.00169.x

Smith, R.J., 1999. Statistics of sexual size dimorphism. J. Human Evol. 36, 423–459. Doi:10.1006/jhev.1998.0281

Surkova EN, Warburton EM, van der Mescht L, Khokhlova IS, Krasnov BR. 2018. Body size and ecological traits in fleas parasitic on small mammals in the Palearctic: larger species attain higher abundance. Oecologia 188:559-569. Doi: 10.1007/s00442-018-4235-y

Upham, N.S., Esselstyn, J.A., Jetz, W., 2019. Inferring the mammal tree: Species-level sets of phylogenies for questions in ecology, evolution, and conservation. PLoS Biology 17, e3000494. Doi:10.1371/journal.pbio.3000494

**Supplementary Text S2. Host traits: rationale, explanations, and sources of information.**

From a parasite perspective, host body mass may influence a parasite’s abundance (due to the obvious reasons) and host specificity. Host body mass is associated with the persistence of a host individual in time merely because a larger host species lives longer and, thus, represents a more predictable resource for a parasite (Peters 1983). As a result, parasite species with higher host specificity usually exploit larger hosts, whereas small-bodied hosts are exploited mainly by generalist parasites (Krasnov et al. 2006). Investment in “expensive” tissue such as brain may compromise a host’s immune ability and, thus, facilitate parasite infection (Bordes et al., 2011). Hosts with larger geographic ranges would presumably encounter more parasite species. As a result, broadly distributed hosts are exploited by more parasite species than hosts with a narrow distribution (Krasnov et al. 2004). Living in groups or having a short dispersal range may facilitate parasite transmission, thus leading to their increased abundance (Stanko et al. 2002); however, these factors may also decrease this abundance via a “dilution effect” (Mooring and Hart 1992) or sociality-related anti-parasite defences (e.g., allogrooming; Moore 2002). Host sociality may also be associated with a parasite’s characteristic strategy for host search and location (Krasnov et al. 2002a). This suggests that sociality could be an important factor affecting the suitability of a given host for a given parasite. Hibernation substantially changes many morphological and physiological characteristics of a mammalian host. These changes could strongly affect the ability of a haematophagous parasite to exploit a host during hibernation due to, for example, lower body temperature, periodical restriction of peripheral blood flow, and thicker subcutaneous fat layers. Host shelter is of utmost importance for nidicolous parasites. It creates a favourable environment (e.g., temperature and relative humidity) for the development of pre-imaginal stages. In addition, it provides flea larvae with food resources and flea pupae with material for camouflaging their cocoons (Krasnov 2008). Consequently, shelter depth and complexity (number of horizons, exits, and nest chambers, ventilation, etc.) could influence an ectoparasite’s host selection. We described shelter depth and complexity as two categorical variables with three levels each (shelter depth: shallow: aboveground shelter or shelter in a rock crevice; intermediate: burrow with depth up to 1 m; deep: burrow with depth more than 1 m; shelter complexity: simple: simple shelter or burrow with 1–2 exits; intermediate: burrow with a single horizon and up to 10 exits; complex: burrow with multiple horizons and more than 10 exits). Data on host traits were taken from the PANTHERIA database (Jones et al. 2009), Krasnov et al. (2016, 2023), and the COMBINE database (Soria et al. 2021).

**References**

Bordes F, Blumstein DT, Morand S. 2007. Rodent sociality and parasite diversity. Biol. Lett. 3: 692-694. Doi: 10.1098/rsbl.2007.0393

Jones, K.E., Bielby, J., Cardillo, M., Fritz, S.A., O'Dell, J., Orme, C.D.L. et al. (2009). PanTHERIA: a species-level database of life history, ecology, and geography of extant and recently extinct mammals. Ecology, 90, 2648-2648. Doi:10.1890/08-1494.1

Krasnov, B.R. 2008. Functional and evolutionary ecology of fleas. A model for ecological parasitology. - Cambridge Univ. Press.

Krasnov, B.R., Khokhlova, I.S., Oguzoglu, I., Burdelova, N.V. 2002. Host discrimination by two desert fleas using an odour cue. Anim. Behav. 64: 33-40. Doi:10.1006/anbe.2002.3030

Krasnov, B.R., Shenrot, G.I., Khokhlova, I.S., Degen, A.A. 2004. Flea species richness and parameters of host body, host geography and host ‘milieu’. J. Anim. Ecol. 73: 1121-1128. Doi:10.1111/j.0021-8790.2004.00883.x

Krasnov BR, Morand S, Mouillot D, Shenbrot GI, Khokhlova IS, Poulin R. 2006. Resource predictability and host specificity in fleas: the effect of host body mass. Parasitology 133: 81-88. Doi: 10.1017/S0031182006000059

Krasnov BR, Shenbrot GI, Khokhlova IS and Degen AA (2016) Trait-based and phylogenetic associations between parasites and their hosts: a case study with small mammals and fleas in the Palearctic. Oikos 125, 29-38. Doi:10.1111/oik.02178

Krasnov BR, Grabovsky VI, Khokhlova IS, López Berrizbeitia MF, Matthee S, Roll U, Sanchez JP, Shenbrot GI and van der Mescht L (2023) Latitudinal distributions of the species richness, functional diversity, and phylogenetic diversity of fleas and their small mammalian hosts in four geographic quadrants. Ecography 2024:e07129. Doi:10.1111/ecog.07129

Moore, J. 2002. Parasites and the behavior of animals. Oxford Univ. Press.

Mooring, M.S. and Hart, B.L. 1992. Animal grouping for protection from parasites: selfish herd and encounter-dilution effects. Behaviour 123: 173-193. Doi:10.1163/156853992X00011

Peters, R.H. 1983. The ecological implications of body size. Cambridge Univ. Press.

Soria, C.D., Pacifici, M., Di Marco, M., Stephen, S.M. & Rondinini, C. (2021). COMBINE: a coalesced mammal database of intrinsic and extrinsic traits. Ecology, 102, e03344. Doi:10.1002/ecy.3344

Stanko M, Miklisová D, Goüy de Bellocq J, Morand S.. 2002. Mammal density and patterns of ectoparasite species richness and abundance. Oecologia 131: 289-295. Doi: 10.1007/s00442-002-0889-5
